# Shaping immune landscape of colorectal cancer by cholesterol metabolites

**DOI:** 10.1038/s44321-023-00015-9

**Published:** 2024-01-02

**Authors:** Yibing Bai, Tongzhou Li, Qinshu Wang, Weiqiang You, Haochen Yang, Xintian Xu, Ziyi Li, Yu Zhang, Chengsong Yan, Lei Yang, Jiaqian Qiu, Yuanhua Liu, Shiyang Chen, Dongfang Wang, Binlu Huang, Kexin Liu, Bao- Liang Song, Zhuozhong Wang, Kang Li, Xin Liu, Guangchuan Wang, Weiwei Yang, Jianfeng Chen, Pei Hao, Zemin Zhang, Zhigang Wang, Zheng-Jiang Zhu, Chenqi Xu

**Affiliations:** 1 CAS Center for Excellence in Molecular Cell Science, Shanghai Institute of Biochemistry and Cell Biology, University of Chinese Academy of Sciences, Chinese Academy of Sciences, Shanghai, China; 2 Interdisciplinary Research Center on Biology and Chemistry, Shanghai Institute of Organic Chemistry, Chinese Academy of Sciences, University of Chinese Academy of Sciences, Shanghai, China; 3 School of Life Science and Technology, ShanghaiTech University, Shanghai, China; 4 Department of General Surgery, Shanghai Jiao Tong University Affiliated Sixth People’s Hospital, Shanghai, China; 5 CAS Key Laboratory of Molecular Virology and Immunology, Institut Pasteur of Shanghai, Center for Biosafety Mega-Science, Chinese Academy of Sciences, Shanghai, China; 6 Beijing Advanced Innovation Center for Genomics, BIOPIC and School of Life Sciences, Peking University, Beijing, China; 7 Hubei Key Laboratory of Cell Homeostasis, College of Life Sciences, Wuhan University, Wuhan, China; 8 The Second Affiliated Hospital of Harbin Medical University, Harbin, China; 9 Department of Epidemiology and Biostatistics, School of Public Health, Harbin Medical University, Harbin, China

**Keywords:** Colorectal Cancer with Microsatellite Stability, Asynchronous Cholesterol Biosynthesis, Distal Cholesterol Precursors, Th17, Cyp51 Targeted Therapy, Cancer, Digestive System, Metabolism

## Abstract

Cancer immunotherapies have achieved unprecedented success in clinic, but they remain largely ineffective in some major types of cancer, such as colorectal cancer with microsatellite stability (MSS CRC). It is therefore important to study tumor microenvironment of resistant cancers for developing new intervention strategies. In this study, we identify a metabolic cue that determines the unique immune landscape of MSS CRC. Through secretion of distal cholesterol precursors, which directly activate RORγt, MSS CRC cells can polarize T cells toward Th17 cells that have well-characterized pro-tumor functions in colorectal cancer. Analysis of large human cancer cohorts revealed an asynchronous pattern of the cholesterol biosynthesis in MSS CRC, which is responsible for the abnormal accumulation of distal cholesterol precursors. Inhibiting the cholesterol biosynthesis enzyme Cyp51, by pharmacological or genetic interventions, reduced the levels of intratumoral distal cholesterol precursors and suppressed tumor progression through a Th17-modulation mechanism in preclinical MSS CRC models. Our study therefore reveals a novel mechanism of cancer–immune interaction and an intervention strategy for the difficult-to-treat MSS CRC.

## Synopsis

A metabolic cue shaping the unique immune landscape of colorectal cancer with microsatellite stability (MSS CRC) is identified. Tumor-secreted distal cholesterol precursors (DCPs) polarize type 17 T cells, thereby inducing an immunosuppressive microenvironment to favor tumor progression.

- MSS CRC cells secreted DCPs to polarize Th17 cells.
- The accumulation of DCPs was induced by an asynchronous pattern of the cholesterol biosynthesis in MSS CRC cells.
- Inhibiting the cholesterol biosynthesis enzyme Cyp51 reshaped tumor immune microenvironment and reduced MSS CRC progression.

## Abstract (web summary)

A metabolic cue shaping the unique immune landscape of colorectal cancer with microsatellite stability (MSS CRC) is identified. Tumor-secreted distal cholesterol precursors (DCPs) polarize type 17 T cells, thereby inducing an immunosuppressive microenvironment to favor tumor progression.

## Graphical abstract

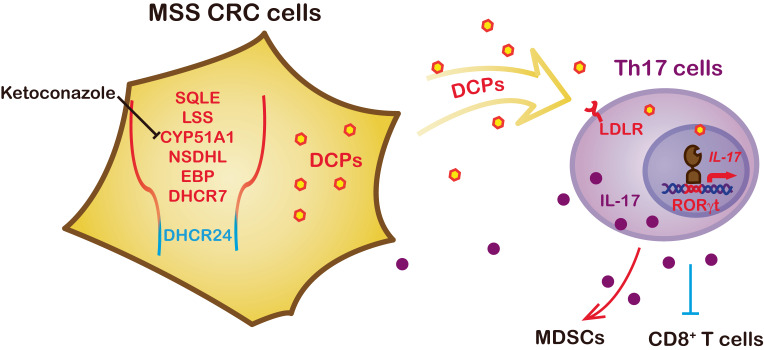

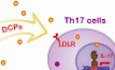

The paper explained **Problem**

Colorectal cancer (CRC) is one of the mostly diagnosed cancer types with high mortality rate. CRC with microsatellite stability (MSS CRC), the major subtype of CRC (~85% of patients), has a unique enrichment of a T cell subset Th17 that has well-characterized roles in tumor progression and immunotherapy resistance. It is thus of high interest to understand the molecular determinant of Th17 enrichment in MSS CRC.

**Results**

We reported a metabolic cue responsible for the Th17 enrichment in MSS CRC by studying large human cohorts and animal models. Through secretion of distal cholesterol precursors (DCPs), MSS CRC cells can polarize T cells toward Th17 lineage. An asynchronous upregulation pattern of the cholesterol biosynthesis is responsible for the abnormal accumulation of DCPs. Inhibiting the cholesterol biosynthesis enzyme Cyp51, by pharmacological or genetic interventions, reduced the levels of intratumoral distal cholesterol precursors and suppressed tumor progression through a Th17-modulation mechanism.

**Impact**

Our study reveals a novel mechanism of cancer–immune interaction and an intervention strategy for the difficult-to-treat MSS CRC.

## Introduction

Colorectal cancer (CRC) is one of the mostly diagnosed cancer types with high mortality rate (Dekker et al, 2019). CRC can be categorized into subtypes with microsatellite stability (MSS; ~85% of patients) and microsatellite instability (MSI; ~15% of patients). MSI CRC is one of the cancer types with best response rate to anti-PD-1 immunotherapy (Lumish and Cercek, 2021). MSS CRC, however, is insensitive to anti-PD-1 and/or anti-CTLA4 treatment, although MSS CRC has a comparable level of tumor mutational burden as anti-PD-1 responsive cancers (Picard et al, 2020; Yarchoan et al, 2017). The poor performance of immunotherapies in MSS CRC reflects the urgent need of studying immune landscape of CRC subtypes.

Analyzing CRC immune landscape by various technologies reveals enrichment of a pro-inflammatory CD4^+^ T cell subset Th17 in MSS CRC. In MSI CRC, another CD4^+^ T cell subset Th1 and cytotoxic CD8^+^ T cells are enriched (Le Gouvello et al, 2008; Lee et al, 2020; Mlecnik et al, 2016; Pelka et al, 2021; Zhang et al, 2018). The anti-tumor functions of Th1 and CD8^+^ T cells have been well demonstrated, but the roles of Th17 in different types of cancer are context-dependent (Borst et al, 2018; Jiao et al, 2019; Palucka and Coussens, 2016; Zhao et al, 2020; Zou and Restifo, 2010). Interleukin 17A (IL-17A), the major cytokine secreted by Th17, is known to promote CRC progression via both immune and cancer modulation mechanisms (Hurtado et al, 2018; Razi et al, 2019; Tosolini et al, 2011). High IL-17A level is associated with poor prognosis in CRC, and ablation of IL-17A can prevent CRC metastasis (Tseng et al, 2014). Moreover, IL-17 mediates PD-1 resistance in multiple cancer types by recruiting neutrophils (Akbay et al, 2017; Llosa et al, 2019; Zhang et al, 2020b). Therefore, it is of high clinical interest to understand molecular determinants of Th17 enrichment in MSS CRC.

## Results

### MSS CRC cells polarize Th17 cells via a lipid cue

**Figure 1:**
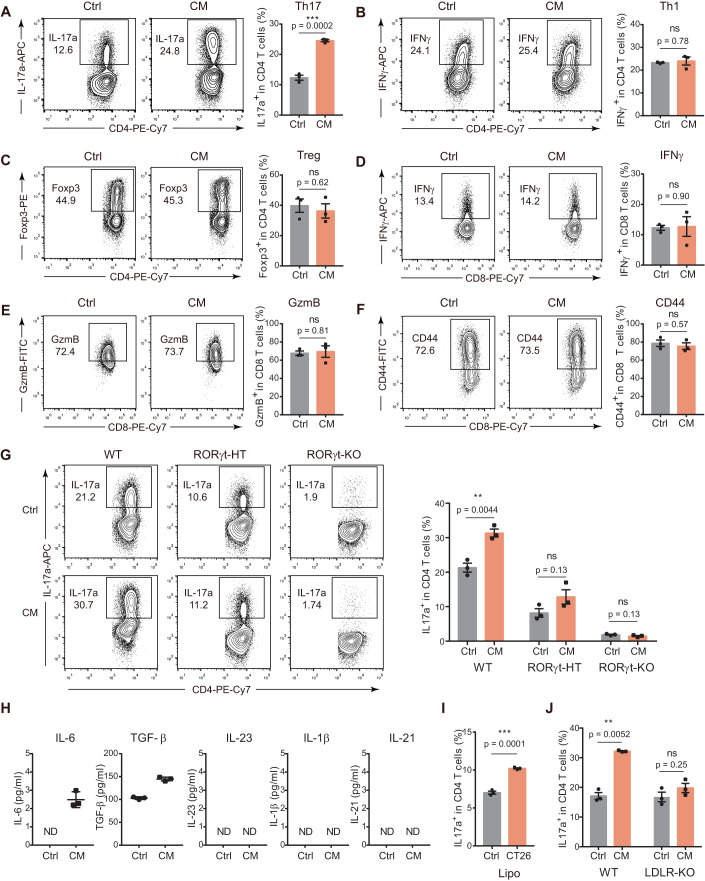
Specific polarization of Th17 cells by secreted lipophilic factors of MSS CRC cells. (**A**–**C**) Th17 (**A**), Th1 (**B**), and Treg (**C**) induction in the presence of the conditioned medium of CT26 cells. Naive CD4^+^ T cells were stimulated with plate-bound anti-CD3/CD28 and cytokines to induce Th17, Th1 or Treg differentiation. Concentrated conditioned medium (CM) or control blank medium (Ctrl) (2.5% v/v) was added on Day 2. Th17 (IL-17a^+^ gated on CD4^+^), Th1 (IFNγ^+^ gated on CD4^+^), and Treg (Foxp3^+^ gated on CD4^+^) percentages were analyzed on Day 4 (*n* = 3). (**D**, **E**) CD8^+^ T cell effector functions in the presence of the conditioned medium of CT26 cells. Naive CD8^+^ T cells were stimulated with plate-bound anti-CD3/CD28. Concentrated conditioned medium (CM) or control blank medium (Ctrl) (2.5% v/v) was added on Day 1. CD8^+^ T cell effector functions (**D**) IFNγ^+^ gated on CD8^+^; (**E**) GzmB^+^ gated on CD8^+^) was analyzed on Day 2 (*n* = 3). (**F**) CD8^+^ T cell activation in the presence of the conditioned medium of CT26 cells. Naive CD8^+^ T cells were stimulated with plate-bound anti-CD3/CD28, in the presence of concentrated conditioned medium (CM) or control blank medium (Ctrl) (2.5% v/v). CD8^+^ T cell activation (CD44^+^ gated on CD8^+^) was analyzed on Day 1 (*n* = 3). (**G**) Th17 induction of *Rorc*^*-/-*^(RORγt-KO), *Rorc*^*+/-*^(RORγt-HT) or *Rorc*^*+/+*^ (WT) naive CD4^+^ T cells in the presence of the conditioned medium of CT26 cells (*n* = 3). (**H**) Levels of indicated cytokines in the conditioned medium of CT26 cells. ND not detected (*n* = 3). (**I**) Th17 induction in the presence of the lipoproteins isolated from CT26 CM or lipoproteins isolated from control blank medium (*n* = 3). (**J**) Th17 induction of *Ldlr*^*-/-*^ (LDLR-KO) or *Ldlr*^*+/+*^(WT) naive CD4^+^ T cells in the presence of the conditioned medium of CT26 cells. (*n* = 3). Data information: in (**A**–**G**), representative flow cytometry plots are shown in the left and corresponding quantified data in the right. In (**A**–**J**), data are presented as mean ± SEM. In (**A**–**G**, **I**, **J**), two-tailed unpaired Student’s *t* test was used when variances were similar, whereas a two-tailed unpaired *t* test with Welch’s correction was used when variances were different. Data are representative of five (**A**) or two (**B**–**J**) independent experiments. *P* levels < 0.01**, < 0.001***. Source data are available online for this figure.

**Figure EV1:**
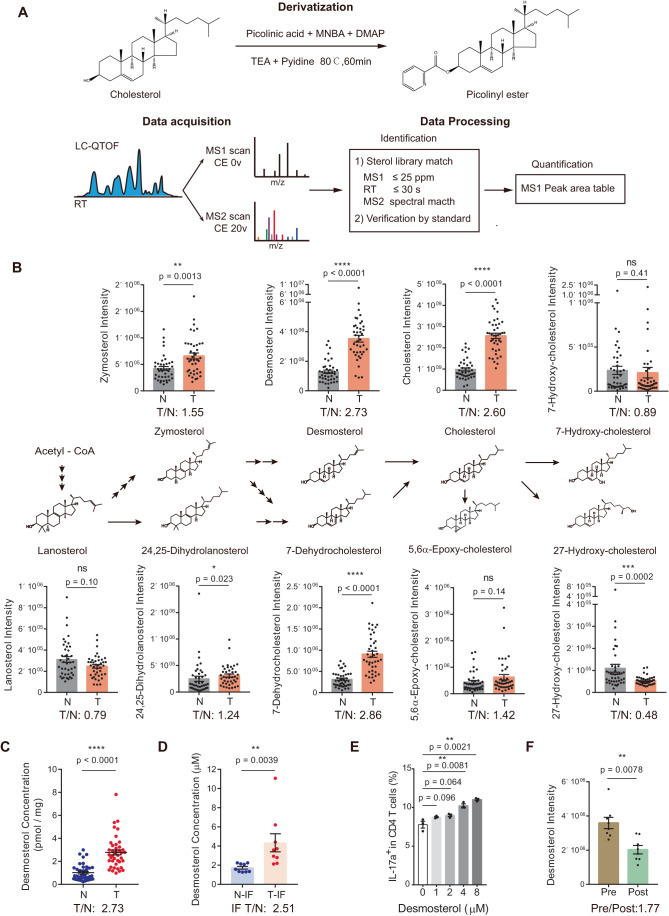
Caco2 cells polarized Th17 cells without affecting other T cell subtypes. (**A**–**C**) Th17 (**A**), Th1 (**B**), and Treg (**C**) induction in the presence of the conditioned medium of Caco2 cells. Naive CD4^+^ T cells were stimulated with plate-bound anti-CD3/CD28 and cytokines to induce Th17, Th1 or Treg differentiation. Concentrated conditioned medium (CM) or control blank medium (Ctrl) (2.5% v/v) was added in on Day 2. Th17 (IL-17a^+^ gated on CD4^+^), Th1 (IFNγ^+^ gated on CD4^+^), and Treg (Foxp3^+^ gated on CD4^+^) percentages were analyzed on Day 4 (*n* = 4). (**D**, **E**) CD8^+^ T cell effector functions in the presence of the conditioned medium of Caco2 cells. Naive CD8^+^ T cells were stimulated with plate-bound anti-CD3/CD28. Concentrated conditioned medium (CM) or control blank medium (Ctrl) (2.5% v/v) was added in on Day 1. CD8^+^ T cell function (**D**) IFNγ^+^ gated on CD8^+^; (**E**) GzmB^+^ gated on CD8^+^ was analyzed on Day 2 (*n* = 4). (**F**) CD8^+^ T cell activation in the presence of the conditioned medium of Caco2 cells. Naive CD8^+^ T cells were stimulated with plate-bound anti-CD3/CD28, in the presence of concentrated conditioned medium (CM) or control blank medium (Ctrl) (2.5% v/v). CD8^+^ T cell activation (CD44^+^ gated on CD8^+^) was analyzed on Day 1. (*n* = 4). Data information: in (**A**–**F**), data are presented as mean ± SEM. Representative flow cytometry plots are shown in the left and corresponding quantified data in the right; two-tailed unpaired Student’s *t* test. Data are representative of two independent experiments. *P* level < 0.01**. Source data are available online for this figure.

We collected conditioned media (CM) of two commonly used MSS CRC cell lines: the mouse CT26 cells and the human Caco2 cells. Supplement of the CRC media to cell culture promoted polarization of naive CD4^+^ T cells to the Th17 lineage, but not the Th1 or Treg lineage (Figs. 1A–C and EV1A–C). We also added MSS CRC CM to CD8^+^ T cell culture and observed no effect on cell activation and effector function, indicated by the CD44 surface level and cytokine productions, respectively (Figs. 1D–F and EV1D–F). Thus, MSS CRC CM appeared to have specific polarization effect on Th17 cells. Retinoic acid receptor–related orphan receptor γ (RORγt) is the master transcription factor of Th17 polarization (Ivanov et al, 2006), and its deficiency, even partially, abolished the Th17 polarization effect of MSS CRC CM (Fig. 1G).

Th17 can be induced by both cytokine and metabolic cues. The Th17-inducing cytokines in the CT26 CM were at trace-amount levels that are far below the cytokine concentrations commonly used to induce Th17 (Fig. 1H). On the other hand, the isolated lipoproteins from CT26 CM could polarize Th17, indicating lipophilic molecules could mediate Th17 polarization (Fig. 1I). In addition, the depletion of low-density lipoprotein receptor (LDLR) in T cells abolished the Th17 polarization by CT26 CM (Fig. 1J), further demonstrating the dependence of lipophilic molecules in MSS CRC promoted Th17 polarization.

### Accumulation of distal cholesterol precursors in human MSS CRC microenvironment

**Figure 2:**
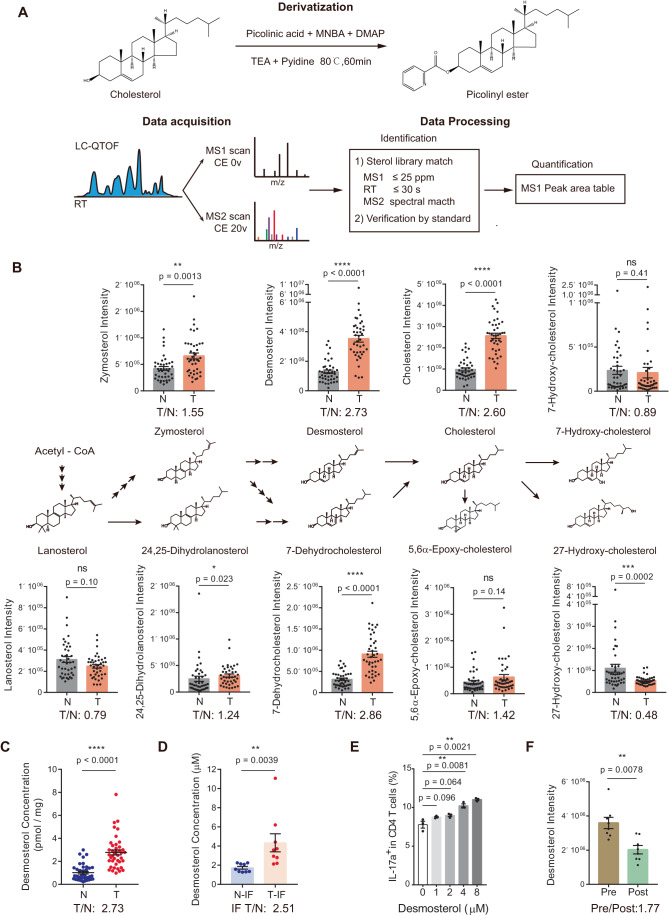
Human MSS CRC tumors had elevated levels of distal cholesterol precursors. (**A**) Experimental approach for the LC-MS based sterol analysis. (**B**) Intensity of the identified sterols in human MSS CRC tumors (T) and paired adjacent normal tissues (N). Fold change of the mean level of each sterol in tumor vs normal tissue (T/N) is labeled under each graph. Two-tailed Wilcoxon matched-pairs signed rank test (*n* = 41). (**C**) Absolute quantification of desmosterol in the human MSS CRC tumors and paired adjacent normal tissues. Fold change of the mean level of desmosterol in tumors vs normal tissues (T/N) is labeled under graph. Two-tailed Wilcoxon matched-pairs signed-rank test (*n* = 41). (**D**) Absolute quantification of desmosterol in the interstitial fluid of human MSS CRC tumors (T-IF) and paired adjacent normal tissues (N-IF). Fold change of the mean level of desmosterol in tumors interstitial fluid vs normal tissue interstitial fluid (IF T/N) is labeled under graph. Two-tailed Wilcoxon matched-pairs signed-rank test (*n* = 9). (**E**) Th17 induction in the presence of desmosterol at indicated concentrations. Naive CD4^+^ T cells were stimulated with plate-bound anti-CD3/CD28 and cytokines to induce Th17 differentiation. Two-tailed unpaired Student’s *t* test (*n* = 3). (**F**) Intensity of the desmosterol in the plasma samples of human MSS CRC patients before (Pre) and after (Post) surgical resection of tumors. Fold change of the mean level of each sterol in plasma before vs after surgical resection (Pre/Post) is labeled under each graph. Two-tailed Wilcoxon matched-pairs signed-rank test (*n* = 8). Data information: in (**B**–**F**), data are presented as mean ± SEM. *P* levels < 0.05*, < 0.01**, < 0.001***, < 0.0001****. Source data are available online for this figure.

Native RORγt agonists are produced by the cholesterol biosynthesis pathway. Distal cholesterol precursors (DCPs) after lanosterol and before cholesterol could bind to the RORγt ligand binding domain to promote coactivator recruitment and they were capable of polarizing Th17 when supplemented into culture medium (Hu et al, 2015; Kidani and Bensinger, 2017; Santori et al, 2015). We therefore hypothesized that MSS CRC cells might polarize Th17 cells by secreted cholesterol precursors. We firstly measured sterols in human MSS CRC primary tumors and paired adjacent normal tissues by a LC-MS based sterol profiling method (Fig. 2A, Table 1). The picolinic acid derivatization method was used to increase sterol ionization efficiency and detection sensitivity (Qiu et al, 2021). Nine sterols in the KEGG sterol pathways of mammalian cells were quantitatively measured (Fig. 2B). All sterol identifications were further validated using purchased chemical standards (Appendix Fig. S1). Cholesterol levels increased in the tumor samples, which could be contributed by either active de novo biosynthesis or enhanced uptake. Distal cholesterol precursors with RORγt agonistic function, such as desmosterol, 7-dehydrocholesterol and zymosterol, were enriched in the MSS CRC tumors, reflecting the upregulated cholesterol biosynthesis. Levels of oxysterols were not elevated in MSS CRC tumors though.

**Table 1:** Demographic and clinical characteristics of CRC patients in the cohort for whole tissue samples.

Characteristics	Paired sample
	Whole tissue
Total number	41
Age (mean ± sd, year)	60.4 ± 9.9
Gender (male/female)	25/16
BMI (mean ± sd)	22.9 ± 2.5
Blood type (A/B/AB/O)	12/15/6/8
Smoke (yes/no)	18/23
Drink (yes/no)	12/29
CEA (median, range, ng/ml)	5.39, 0.80–146.3
CA19-9 (median, range, U/ml)	11.18, 0.85–774.4
Tumor size (mean ± sd, cm)	4.8 ± 1.7
Stage (I/II/III/IV)	4/17/18/2
Microsatellite status (MSS/MSI)	41/0

*CEA* carcinoembryonic antigen, *CA19-9* carbohydrate antigen 199.

**Figure EV2:**
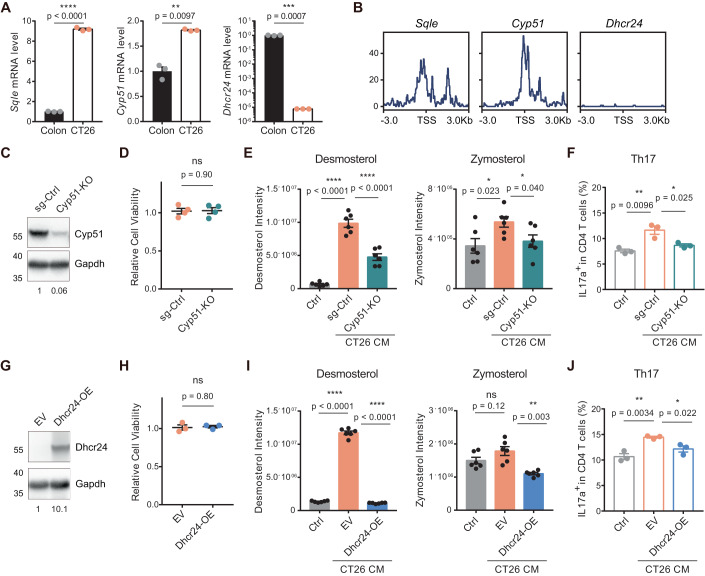
Accumulation of distal cholesterol precursors in the tumor microenvironment of MSS CRC. (**A**) Intensity of sterols in the interstitial fluid of human MSS CRC tumors (T-IF) and paired adjacent normal tissues (N-IF). Fold change of the mean level of each sterol in tumor interstitial fluid vs normal tissue interstitial fluid (T/N) is labeled under each graph (*n* = 36). (**B**) Th1 induction in the presence of desmosterol at different concentrations. Naive CD4^+^ T cells were stimulated with plate-bound anti-CD3/CD28 and cytokines to induce Th1 differentiation, in the presence of desmosterol or vehicle control. Th1 (IFNγ^+^ gated on CD4^+^) polarization were analyzed on Day 4 (*n* = 4). (**C**) Treg induction in the presence of desmosterol at different concentrations. Naive CD4^+^ T cells were stimulated with plate-bound anti-CD3/CD28 and cytokines to induce Treg differentiation, in the presence of desmosterol or vehicle control. Treg (Foxp3^+^ gated on CD4^+^) polarization were analyzed on Day 4 (*n* = 3). (**D**) Th17 induction in the presence of desmosterol at different concentrations. Naive CD4^+^ T cells from *Rorc*^*-/-*^ (RORγt-KO), *Rorc*^*+/-*^ (RORγt-HT) or *Rorc*^*+/+*^ (WT) mice were stimulated plate-bound anti-CD3/CD28 and cytokines to induce Th17, in the presence of desmosterol or vehicle control. Th17 (IL-17a^+^ gated on CD4^+^) percentage was analyzed on Day 4 (*n* = 4). (**E**, **F**) CD8^+^ T cell function in the presence of desmosterol at different concentrations. Naive CD8^+^ T cells were stimulated with plate-bound anti-CD3/CD28, in the presence of desmosterol or vehicle control. CD8^+^ T cell function (**E**) IFNγ^+^ gated on CD8^+^; (**F**) GzmB^+^ gated on CD8^+^ were analyzed on Day 2 (*n* = 4). (**G**) CD8^+^ T cell activation in the presence of desmosterol at different concentrations. Naive CD8^+^ T cells were stimulated with plate-bound anti-CD3/CD28, in the presence of desmosterol or vehicle control. CD8^+^ T cell activation (CD44^+^ gated on CD8^+^) were analyzed on Day 1 (*n* = 4). (**H**) Absolute quantification of zymosterol and 7-dehydrocholesterol in the interstitial fluid of human MSS CRC tumors (T-IF) and paired adjacent normal tissues (N-IF). Fold change of the mean level of indicated sterol in tumors interstitial fluid vs normal tissue interstitial fluid (IF T/N) is labeled under graph (*n* = 9). (**I**, **J**) Intensity of indicated distal cholesterol precursors in the control blank medium (Ctrl) and the conditioned media of mouse MSS CRC cell CT26 (*n* = 10), mouse MSI CRC cell MC38 (*n* = 10), human MSS CRC cell Caco2 (*n* = 6), and human MSI CRC cell HCT116 (*n* = 6). Two-tailed unpaired *t* test was used when variances were similar, whereas a two-tailed unpaired *t* test with Welch’s correction was used when variances were different. (**K**) Intensities of the ^13^C-labeled sterols in CT26 and MC38 cells (*n* = 6). Two-tailed unpaired *t* test was used when variances were similar, whereas a two-tailed unpaired *t* test with Welch’s correction was used when variances were different. (**L**) Th17 induction in the presence of the conditioned medium of MC38 cells (*n* = 3). (**M**) Intensity of the sterols in the plasma samples of human MSS CRC patients before (Pre) and after (Post) surgical resection of tumors. Fold change of the mean level of each sterol in plasma before vs after surgical resection (Pre/Post) is labeled under each graph (*n* = 8). Data information: in (**A**–**M**), data are presented as mean ± SEM. In (**B**–**G**, **L**) two-tailed unpaired Student’s *t* test; data are representative of two independent experiments. In (**A**, **H**, **M**), two-tailed Wilcoxon matched-pairs signed-rank test. *P* levels < 0.05*, < 0.01**, < 0.001***, < 0.0001****. Source data are available online for this figure.

We further measured sterol levels in the interstitial fluid of MSS CRC primary tumors and paired adjacent normal tissues (Figure EV2A, Table 2). Enrichment of the distal cholesterol precursors in the tumor microenvironment was evident. Absolute quantification of desmosterol, the most potent DCP RORγt agonist reported (Hu et al, 2015; Santori et al, 2015), were performed. Absolute quantifications show that desmosterol levels were ~2.7 folds higher in tumor tissues than in normal tissues (Fig. 2C). The desmosterol concentration in the tumor interstitial fluid of MSS CRC was around 4 μM, 2.5-fold higher than in the normal colons (Fig. 2D). At this level, desmosterol was able to polarize the Th17 subtype and had no evident effect on other T cell subtypes (Figs. 2E and EV2B-G). Absolute quantifications of zymosterol and 7-dehydrocholesterol in interstitial fluid samples further confirmed the 2-4-fold enrichment of distal cholesterol precursors in MSS CRC (Figure EV2H).

**Table 2:** Demographic and clinical characteristics of CRC patients in the cohort for interstitial fluid samples.

Characteristics	Paired sample
	Interstitial fluid
Total number	36
Age (mean ± sd, year)	66.0 ± 11.1
Gender (male/female)	22/14
BMI (mean ± sd)	22.9 ± 2.7
Blood type (A/B/AB/O)	11/13/2/10
Smoke (yes/no)	12/24
Drink (yes/no)	4/32
CEA (median, range, ng/ml)	5.07, 1.06–138.40
CA19-9 (median, range, U/ml)	15.02, 0.90–218.00
Tumor size (mean ± sd, cm)	4.9 ± 1.5
Stage (I/II/III/IV)	0/20/15/1
Microsatellite status (MSS/MSI)	36/0

In line with the human samples, the conditioned media of MSS-CRC CT26 cells (mouse) and Caco2 cells (human) also had aberrantly high levels of distal cholesterol precursors. As a comparison, the conditioned media of MSI-CRC MC38 cells (mouse) and HCT116 cells (human) had no clear accumulation of these metabolites (Figure EV2I,J). Flux analysis confirmed the aberrant enrichment of distal cholesterol precursors in the MSS CRC cells over the MSI CRC cells (Figure EV2K). Indeed, MC38 CM did not have the Th17 polarization effect as CT26 CM (Figure EV2L). Altogether, distal cholesterol precursors with RORγt agonistic function were enriched in MSS CRC microenvironment, which could promote Th17 polarization at physiological concentration.

We also measured the levels of sterols in peripheral plasma samples obtained from MSS CRC patients before and after surgical removal of tumors (Figs. 2F and EV2M, Table 3). Desmosterol levels in blood were significantly reduced by 1.77 folds after surgery, which agrees with the secretion of distal cholesterol precursors by MSS CRC cells.

**Table 3:** Demographic and clinical characteristics of CRC patients in the cohort for plasma samples.

Characteristics	Paired sample
	Plasma
Total number	8
Age (mean ± sd, year)	58.6 ± 10.1
Gender (male/female)	4/4
BMI (mean ± sd)	23.4 ± 2.9
Blood type (A/B/AB/O)	5/2/1/0
Smoke (yes/no)	3/5
Drink (yes/no)	2/6
CEA (median, range, ng/ml)	2.42, 0.80–6.42
CA19-9 (median, range, U/ml)	8.65, 1.24–14.21
Tumor size (mean ± sd, cm)	4.3 ± 1.5
Stage (I/II/III/IV)	2/4/2/0
Microsatellite status (MSS/MSI)	8/0

### Human MSS CRC tumors have an asynchronous pattern of cholesterol biosynthesis pathway

**Figure 3:**
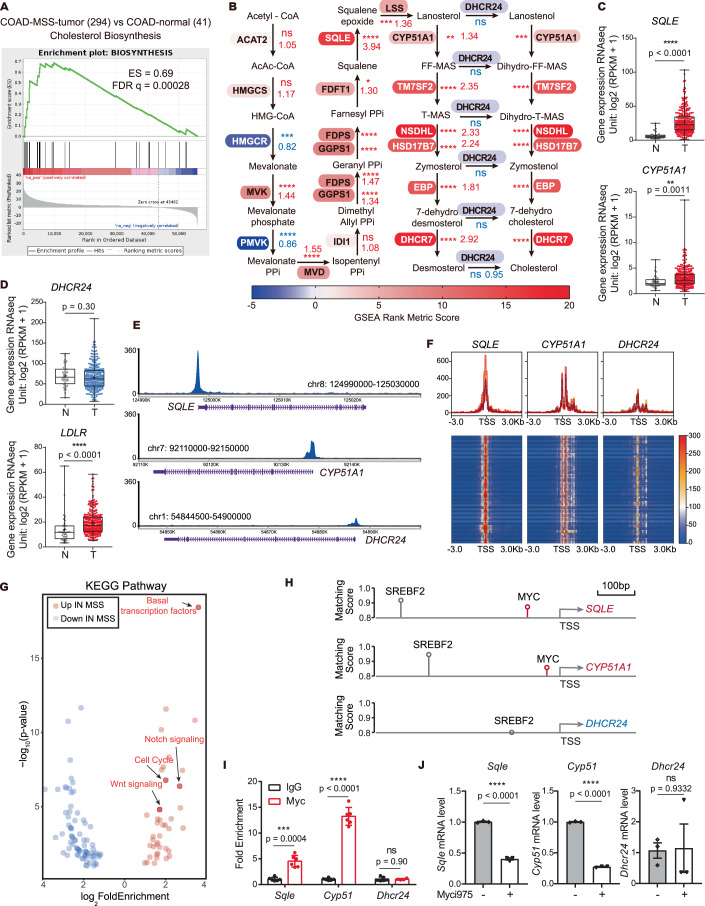
An asynchronous upregulation pattern of the cholesterol biosynthesis pathway in human CRC. (**A**) Gene set enrichment analysis (GSEA) of the cholesterol biosynthesis pathway in MSS CRC tumor tissues (*n* = 294) and normal tissues (*n* = 41) in the TCGA colon adenocarcinoma (COAD) database. (**B**) GSEA rank metric scores and transcriptional levels of enzymes in the cholesterol biosynthesis pathway. Blue and red represents downregulation and upregulation in tumors. *P* value of gene expression between MSS CRC tumors (*n* = 294) and normal tissues (*n* = 41) (two tailed Mann-Whitney test) is labeled by the side of each gene. (ns *P* > 0.05, *P ≤ 0.05, **P < 0.01, ***P < 0.001, ****P < 0.0001). (**C**, **D**) Transcriptional levels of indicated genes in MSS CRC tumors (*n* = 294) and normal tissues (*n* = 41) from COAD. Whiskers denote minimum to maximum. Box indicates the interquartile range (25–75%); center line indicates the median. “+” indicates mean. Two tailed Mann–Whitney test. (**E**) Representative ATAC-seq sequencing tracks of the *SQLE*, *CYP51A1* and *DHCR24* gene locus in tumor sample in TCGA-COAD. Regions shown represent: *SQLE* chr8: 124990000 to 125030000, *CYP51A1* chr7:92110000 to 92150000, *DHCR24* chr1:54844500 to 54900000. (**F**) Top panel, ATAC-seq sequencing tracks of the 6000-bp regions centered at the transcription start site (TSS) of indicated genes from 81 CRC tumor samples in TCGA-COAD (*n* = 81). Bottom panel, heatmap representation of the ATAC-seq chromatin accessibility of TSS regions of indicated genes. Each row represents one CRC tumor sample (*n* = 81). (**G**) Scatterplots of differentially enriched KEGG signaling pathways in MSS CRC relative to normal colon tissues (Fisher’s Exact test, *P* ≤ 0.01 and fold-change ≥2). Representative enriched pathways are indicated by arrowheads. (**H**) Detailed view of *SQLE*, *CYP51A1* and *DHCR24* promoters for enriched TF motifs. The lollipops plots depict the location and matching score of TF motifs found in gene promoters. (**I**) Analysis of direct Myc binding in *Sqle*, *Cyp51* or *Dhcr24* promoters by ChIP-qPCR in CT26 cells. The Myc ChIP signals were normalized to the IgG control and the fold enrichment is shown. For *Sqle* and *Cyp51* (*n* = 6), two-tailed unpaired Student’s *t* test with Welch’s correction (*n* = 6); for *Dhcr24*, two-tailed unpaired Student’s *t* test (Myc, *n* = 4; IgG, *n* = 6). Data are representative of three independent experiments. (**J**) Transcriptional levels of *Sqle*, *Cyp51* and *Dhcr24* in CT26 cells treated with Myci975 (2 μM) or vehicle for 24 h (*n* = 3). Data are representative of two independent experiments. Data information: in (**I**–**J**), data are presented as mean ± SEM. *P* levels < 0.05*, < 0.01**, < 0.001***, < 0.0001****. Source data are available online for this figure.

To understand the reason accounting for the aberrant accumulation of distal cholesterol precursors, we firstly analyzed the single cell RNA sequencing results of human MSS CRC tumors (Zhang et al, 2020a). Among all immune and non-immune cell populations, tumor cells displayed the highest levels of cholesterol biosynthesis, suggesting they could be the primary source of distal cholesterol precursors (Appendix Fig. S2). Since the single cell RNA-seq data has insufficient coverage of the whole cholesterol biosynthesis pathway, we analyzed the bulk RNA-sequencing data from the human Colon Adenocarcinoma (COAD) database in The Cancer Genome Atlas (TCGA) to understand the precise pattern of cholesterol biosynthesis. In total, 294 MSS CRC primary tumor samples and 41 normal samples isolated from CRC patients were available. Human MSS CRC tumors were highly enriched for genes in cholesterol biosynthesis pathway (Fig. 3A). However, the enzymes along the pathway in the MSS CRC tumors had asynchronous alternations when compared with the normal tissues (Fig. 3B). The enzymes of the proximal branch (from ACAT2 to PMVK) showed inconsistent alterations in tumors, meanwhile most distal branch enzymes were significantly upregulated in tumors, including the rate-limiting enzyme SQLE with 3.94-fold increase (Fig. 3C). High expression of SQLE was associated with poor survival of CRC patients in a previous study (Kim et al, 2019). DHCR24, the last enzyme in the distal branch that converts desmosterol to cholesterol (Zerenturk et al, 2013), was largely unchanged or even slightly downregulated (Fig. 3D). This asynchronous upregulation pattern of the distal branch could theoretically lead to accumulation of distal cholesterol precursors in the MSS CRC tissues. Besides the de novo biosynthesis, cells can also obtain cholesterol through LDLR-mediated cholesterol uptake from extracellular environment (Jeon and Blacklow, 2005; Luo et al, 2020). The gene expression levels of LDLR were significantly higher in human MSS CRC tumors compared with normal tissues (Fig. 3D), indicating more vigorous cholesterol uptake, which might explain, at least partially, why cholesterol is also enriched in the MSS CRC tumors when cholesterol production from the biosynthesis pathway is not fully efficient because of the asynchronous pattern.

**Figure EV3:**
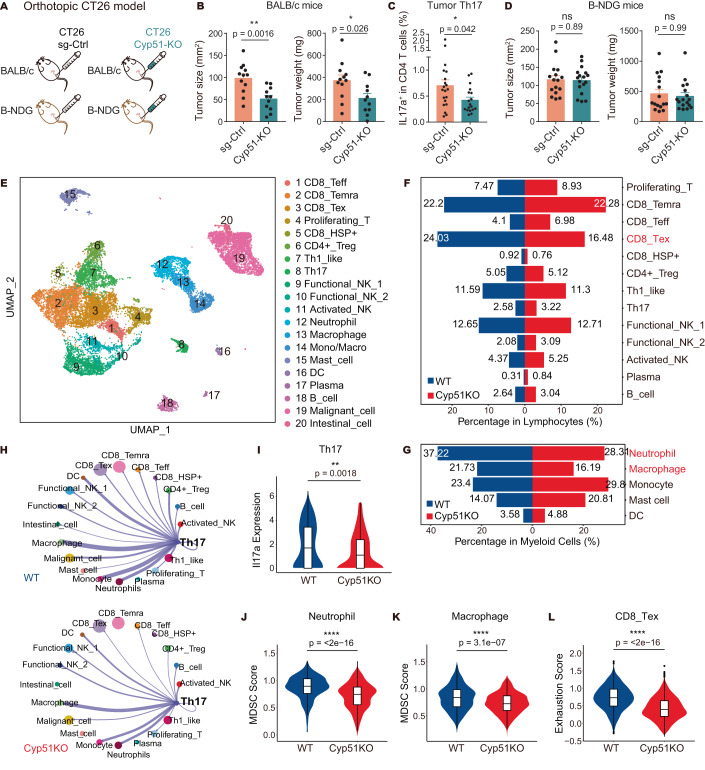
MSS CRC and MSI CRC had different programs of cholesterol metabolism. (**A**) Left: GSEA of cholesterol biosynthesis pathway in MSI-H CRC tumors (*n* = 89) and MSS CRC tumors (*n* = 294) in COAD. Right: GSEA rank metric score and transcriptional levels of enzymes in the cholesterol biosynthesis pathway. Blue and red represents downregulation and upregulation in tumor. *P* value of gene expression between tumor and normal tissue (two tailed Mann–Whitney test) is labeled by the side of each gene. (ns *P* > 0.05, *P ≤ 0.05, **P < 0.01, ***P < 0.001, ****P < 0.0001). (**B**) Transcriptional levels of *LDLR* in MSI tumors (MSI-H-T, *n* = 89) and MSS tumors (MSS-T, *n* = 294) from the COAD database. Whiskers denote minimum to maximum. Box indicates the interquartile range (25–75%); center line indicates the median. “+” indicates mean. Two tailed Mann–Whitney test. Source data are available online for this figure.

We further stratified the human MSS CRC samples by clinical stages and sex of patients. The asynchronous pattern of cholesterol biosynthesis was common (Appendix Figs. S3 and S4A,B). Analysis of paired MSS tumor and adjacent normal tissue from the same CRC patient in COAD (Appendix Fig. S4C), or analysis including normal colon tissues of non-CRC-patient people from Genotype-Tissue Expression (GTEx) database (Appendix Fig. S4D) led to the same conclusion. Of note, when compared with the human MSS CRC tumors, the human MSI-H CRC tumors show less active cholesterol biosynthesis (Figure EV3A) while more active cholesterol uptake (Figure EV3B). These data echoed the lower DCP levels in MSI CRC (Figure EV2I–K).

To gain insight into the asynchronous expression of cholesterol biosynthesis enzymes in MSS CRC tumors, we analyzed chromatin accessibility at proximal promoter regions of the distal branch enzymes by the ATAC-seq data from the COAD (Corces et al, 2018). Consistent with elevated gene expression, the promoters of *SQLE* and *CYP51A1* (the immediate upstream enzymes of distal cholesterol precursors with RORγt agonistic activities) showed higher chromatin accessibility compared to *DHCR24* promoter across different CRC samples (Fig. 3E,F).

We further examined the major signaling pathways in the human MSS CRC tumors based on the transcriptomic profiling from COAD and found that several pathways were upregulated significantly in MSS CRC relative to normal colon tissue, including the Wnt/β-catenin pathway (Fig. 3G). Candidate transcription factor (TF) binding sites or motifs within the proximal promoter regions of the major enzymes along the cholesterol biosynthesis pathway were surveyed and TF binding motifs that are differentially enriched in these gene promoters were identified (Appendix Fig. S5A). Among them, the binding motif of MYC, a major downstream target of the Wnt pathway (Vallee et al, 2021), was enriched only in the gene promoters of cholesterol biosynthesis enzymes upregulated in MSS CRC. Meanwhile, the binding sites of the master regulator, SREBP2, were shared by all the genes (Fig. 3H; Appendix Fig. S5B). Compared with MSI CRC, MSS CRC had clearly upregulated MYC activity (Appendix Fig. S5C). To further validate whether MYC differentially regulate cholesterol biosynthesis enzymes in MSS CRC, we performed ChIP-qPCR assay targeting Myc in CT26 cells. Indeed, Myc preferentially bound to the promoters of *Sqle* and *Cyp51* (mouse ortholog of human *CYP51A1*) but not *Dhcr24* (Fig. 3I). On the other hand, treatment of CT26 cells with a MYC inhibitor Myci975 (Han et al, 2019) reduced the transcriptional levels of *Sqle* and *Cyp51* but not *Dhcr24* (Fig. 3J). These analyses suggested that the ubiquitous SREBP2 and gene-specific MYC cooperate to modulate the expression of cholesterol biosynthesis enzymes in MSS CRC. Altogether, the MSS CRC tumors had an asynchronous upregulation pattern of cholesterol biosynthesis pathway, with higher expression and chromatin accessibility of most distal branch enzymes meanwhile largely unchanged last enzyme, which was orchestrated by the transcription factor SREBP2 and MYC.

### The asynchronous pattern of the cholesterol biosynthesis pathway leads to accumulation of distal cholesterol precursors

**Figure 4:**
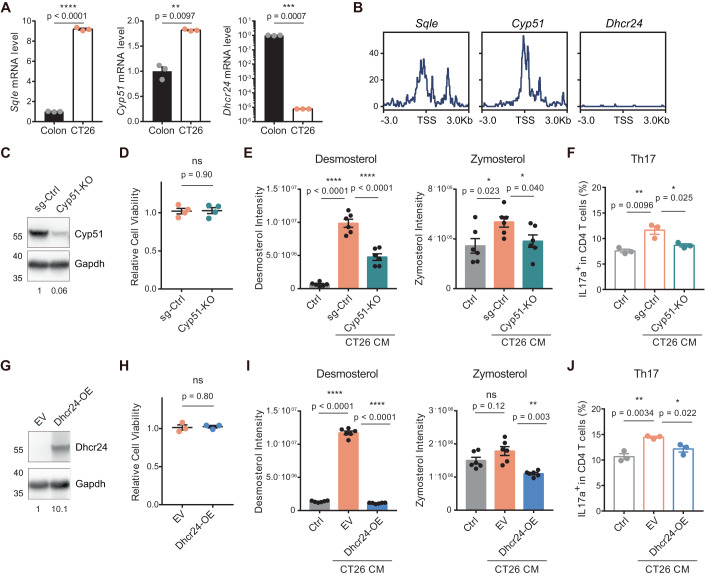
The asynchronous cholesterol biosynthesis pathway led to accumulation of distal cholesterol precursors. (**A**) Transcriptional levels of *Sqle*, *Cyp51* and *Dhcr24* in CT26 cells and normal colon of BALB/c mice (*n* = 3). (**B**) ATAC-seq sequencing tracks of the 6000-bp regions centered at the transcription start site (TSS) of indicated genes from CT26 cells. (**C**) Knockout of *Cyp51* in CT26 cells by sgRNA. Cyp51 protein level was first normalized to Gapdh level, and then normalized to sg-Ctrl. (**D**) Cell viability of Cyp51-KO CT26 cells (Cyp51-KO) and sg-control CT26 cells (sg-Ctrl). Two-tailed unpaired Student’s *t* test (*n* = 4). (**E**) Intensity of indicated distal cholesterol precursors in the control blank medium (Ctrl) and the conditioned media of Cyp51-KO CT26 cells (Cyp51-KO) and sg-control CT26 cells (sg-Ctrl) (*n* = 6). (**F**) IL-17a production of CD4^+^ T cells in the presence of control blank medium, Cyp51-KO CT26 CM, or sg-control CT26 CM. Two-tailed unpaired Student’s *t* test (*n* = 3). (**G**) Ectopic expression of *Dhcr24* in CT26 cells. Dhcr24 protein level was first normalized to Gapdh level, and then normalized to EV. EV empty vector; Dhcr24-OE: *Dhcr24*-overexpression vector. (**H**) Cell viability of Dhcr24-OE CT26 cells and EV-control CT26 cells. Two-tailed unpaired Student’s *t* test (*n* = 3). (**I**) Intensity of indicated distal cholesterol precursors in control blank medium (Ctrl), EV CT26 CM and Dhcr24-OE CT26 CM. (*n* = 6). (**J**) IL-17a production of CD4^+^ T cells in the presence of control blank medium (Ctrl), EV CT26 CM, or Dhcr24-OE CT26 CM. Two-tailed unpaired Student’s *t* test (*n* = 3). Data information: in (**A**, **D**–**F**, **H**–**J**), data are presented as mean ± SEM. In (**A**, **E**, **I**), two-tailed unpaired *t* test was used when variances were similar, whereas a two-tailed unpaired *t* test with Welch’s correction was used when variances were different. In (**C**, **F**, **H**, **J**), data are representative of two independent experiments. *P* levels < 0.05*, < 0.01**, < 0.001***, < 0.0001****. Source data are available online for this figure.

Agreeing with the human MSS CRC tumors, the MSS-CRC CT26 cells had an asynchronous upregulation pattern of cholesterol biosynthesis, as evidenced by the higher transcriptional levels of the upstream enzymes *Sqle* and *Cyp51* and lower transcriptional level of the last enzyme *Dhcr24* than normal mouse colon (Fig. 4A). Chromatin accessibility of *Dhcr24* promoter in CT26 was also markedly lower than that of *Sqle* and *Cyp51* promoters (Fig. 4B), similar to the human samples. Disruption of the asynchronous upregulation pattern of cholesterol biosynthesis by knocking out *Cyp51* in CT26 significantly reduced the accumulation of distal cholesterol precursors in the conditioned medium and the Th17 polarization effect (Fig. 4C–F). On the other hand, overexpressing Dhcr24 to make it less asynchronous with the upstream enzymes showed the similar effect (Fig. 4G–J). Cell survival was not affected by either *Cyp51* knockout or *Dhcr24* overexpression (Fig. 4D,H).

**Figure EV4:**
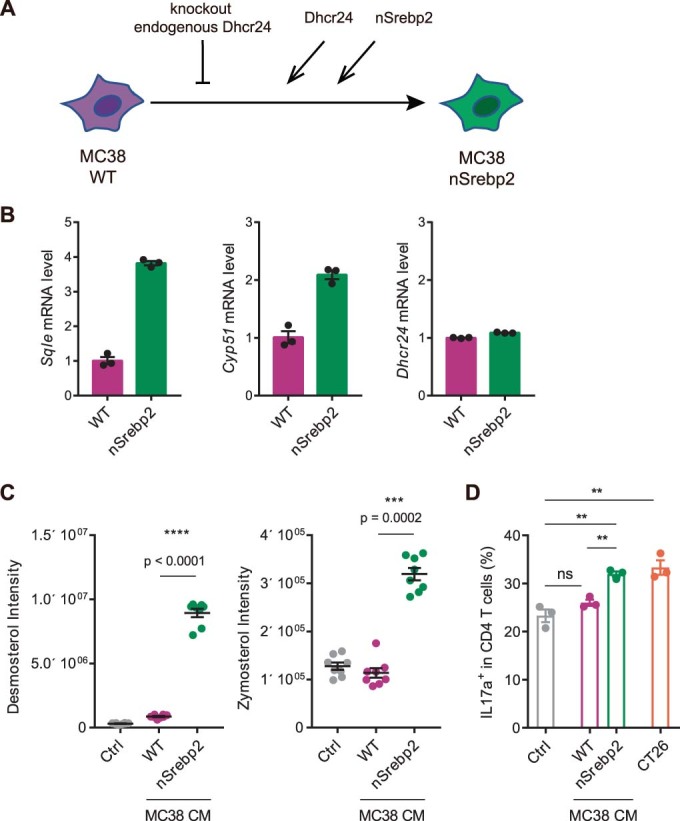
Recapitulation of the asynchronous upregulation pattern of the cholesterol biosynthesis pathway in MSI CRC MC38 cells. (**A**) Schematic illustration of the experimental procedure. (**B**) Transcriptional levels of *Sqle*, *Cyp51* and *Dhcr24* in wildtype (WT) and the genetic manipulated MC38 cells as described in panel A (nSrebp2), *n* = 3. (**C**) Intensity of indicated distal cholesterol precursors in the control blank medium (Ctrl), and the conditioned media of MC38 cells, *n* = 8. Two-tailed unpaired Student’s *t* test if data fitted a normal distribution, Mann–Whitney test if data did not fit a normal distribution. (**D**) Th17 induction with CM of above-mentioned cells or CT26-CM as positive control, *n* = 3, two-tailed unpaired Student’s *t* test. Data information: in (**B**–**D**), data are presented as mean ± SEM. *P* levels < 0.01**, < 0.001***, < 0.0001****. Source data are available online for this figure.

We further recapitulated the asynchronous upregulation pattern of cholesterol biosynthesis in MSI CRC cells. Endogenous *Dhcr24* of MC38 cells was firstly knocked out, followed by stable transfection of *Dhcr24* with a stable PGK promoter to decouple its transcriptional regulation by Srebp2. Next, a truncated version of Srebp2 containing the NH2-terminal DNA-binding domain that could directly enter the nucleus to exert transcriptional activity (Brown et al, 2018) was transduced into the cells to upregulate cholesterol biosynthesis enzymes except Dhcr24 (Figure EV4A). With careful titration, the modified MC38 cell line had normal *Dhcr24* level while 2-4-fold increased expression of upstream genes (Figure EV4B), which mimicked the pattern of MSS CRC tumor. The conditioned media of the modified MC38 cells showed enrichment of distal cholesterol precursors and could induce Th17 polarization (Figure EV4C,D). Altogether, these data showed that the asynchronous upregulation pattern of cholesterol biosynthesis pathway led to the accumulation of distal cholesterol precursors in MSS CRC.

### Intervention of cholesterol biosynthesis reshapes MSS CRC immune landscape and suppresses tumor growth

**Figure 5:**
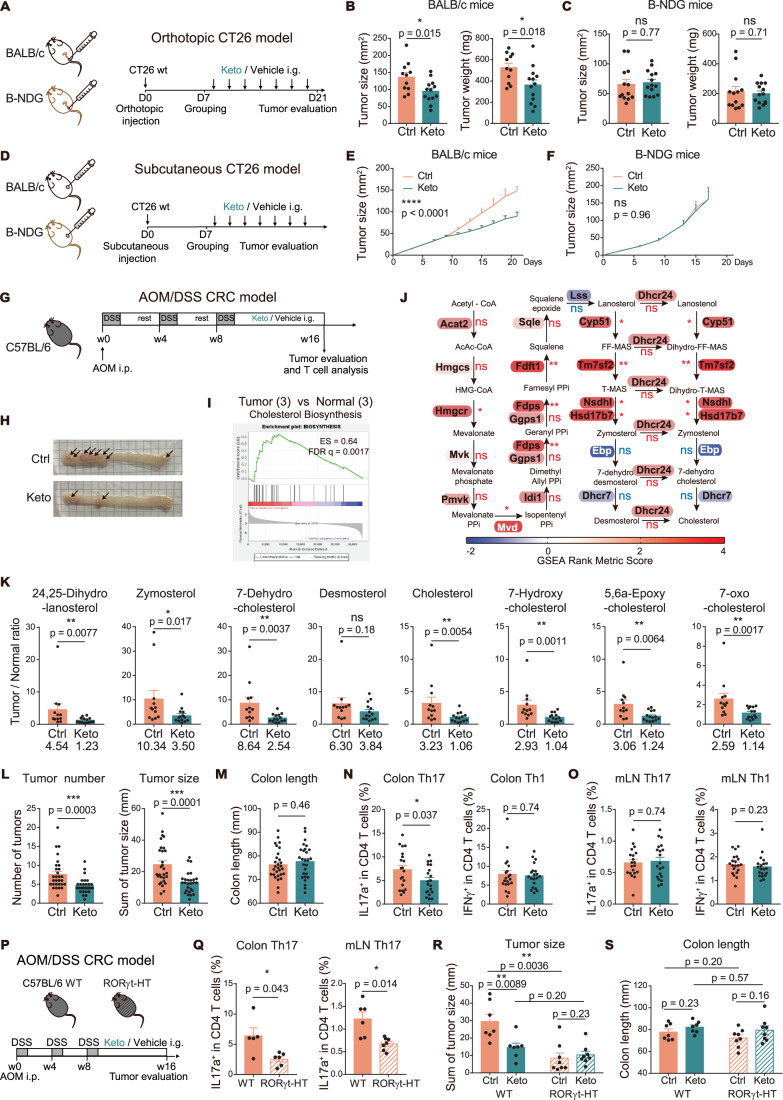
Cyp51 inhibition by ketoconazole suppressed CRC progression through a Th17-dependent mechanism. (**A**–**C**) Orthotopic CT26 transplant model. (**A**) Schematic illustration. (**B**) Tumor burden of orthotopic CT26 transplant MSS CRC in immune-competent BALB/c mice treated with ketoconazole (Keto) or vehicle control (Ctrl) (Ctrl, *n* = 11; Keto, *n* = 13; Data pooled from two independent experiments). Two-tailed unpaired Student’s *t* test. (**C**) Tumor burden of orthotopic CT26 transplant MSS-CRC in lymphocyte-deficient B-NDG mice treated with ketoconazole (Keto) or vehicle control (Ctrl) (Ctrl, *n* = 13; Keto, *n* = 14). Two-tailed unpaired Student’s *t* test. (**D**–**F**) Subcutaneous CT26 model. (**D**) Schematic illustration. (**E**) Tumor progression of subcutaneous CT26 transplant MSS CRC in immune-competent BALB/c mice treated with ketoconazole (Keto) or vehicle control (Ctrl) (*n* = 15). Two-way ANOVA. (**F**) Tumor progression of subcutaneous CT26 transplant MSS CRC in lymphocyte-deficient B-NDG mice treated with ketoconazole (Keto) or vehicle control (Ctrl) (*n* = 5). Data are representative of two independent experiments. Two-way ANOVA. (**G**–**O**) AOM/DSS induced CRC model of wildtype C57BL/6 mice. (**G**) Schematic illustration. (**H**) Representative images of mouse colon with tumors (arrows). (**I**, **J**) Cholesterol biosynthesis pathway of mouse AOM/DSS CRC tumor. (**I**) Gene set enrichment analysis (GSEA) of the cholesterol biosynthesis pathway in tumor tissues (*n* = 3) and adjacent normal tissues (*n* = 3). (**J**) GSEA rank metric scores and transcriptional levels of the cholesterol biosynthesis enzymes. Blue and red represents downregulation and upregulation in tumor. Two-tailed unpaired *t* test *P* value of gene expression between CRC tumors (*n* = 3) and normal tissues (*n* = 3) is labeled by the side of each gene. (**K**) Sterol profiles of mouse AOM/DSS CRC tumors and adjacent normal tissues. Treatment of ketoconazole (Keto) or vehicle control (Ctrl) was performed as shown in (**G**). The ratio of sterol intensity in tumor vs paired adjacent normal tissue (Tumor/Normal ratio) reflected accumulation of sterols in tumors. Mean value of each ratio is labeled under each graph. Two-tailed Mann–Whitney test (Ctrl, *n* = 12; Keto, *n* = 14). (**L**) Tumor burden of mice treated with ketoconazole (Keto, *n* = 27) or vehicle control (Ctrl, *n* = 29). Left panel, total number of tumors on colon. Two tailed Mann-Whitney test. Right panel, total tumor size indicated by sum of the diameter of each tumor. Two-tailed unpaired Student’s *t* test with Welch’s correction. (**M**) Colon length of mice treated with ketoconazole (Keto, *n* = 27) or vehicle control (Ctrl, *n* = 29). Two-tailed unpaired Student’s *t* test. (**N**) Th17 and Th1 infiltration in the colon of mice treated with ketoconazole (Keto) or vehicle control (Ctrl) (*n* = 20). Two-tailed unpaired Student’s *t*-test if data fitted a normal distribution, Mann–Whitney test if data did not fit a normal distribution. (**O**) Th17 and Th1 levels in the mesenteric lymph nodes of mice treated with ketoconazole (Keto) or vehicle control (Ctrl) (*n* = 20). Two-tailed unpaired Student’s *t* test if data fitted a normal distribution, Mann–Whitney test if data did not fit a normal distribution. Data pooled from three independent experiments (**L**–**O**). (**P**–**S**) AOM/DSS induced CRC model of *Rorc*^*+/-*^ C57BL/6 mice. (**P**) Schematic illustration. (**Q**) Left panel, Th17 infiltration in the colon of CRC bearing *Rorc*^*+/-*^ mice (RORγt-HT, *n* = 6) and wildtype mice (WT, *n* = 5). Right panel, Th17 infiltration in the mesenteric lymph nodes of CRC-bearing *Rorc*^*+/-*^ mice (RORγt-HT, *n* = 7) and wildtype mice (WT, *n* = 6). Two-tailed unpaired Student’s *t* test with Welch’s correction. (**R**) Tumor burden of *Rorc*^*+/-*^ or WT mice treated with ketoconazole (Keto) or vehicle control (Ctrl). Total tumor size was indicated by sum of the diameter of each tumor (WT-Ctrl, *n* = 7; WT-Keto, *n* = 7; RORγt-HT-Ctrl, *n* = 8; RORγt-HT-Keto, *n* = 8). Two-tailed unpaired Student’s *t* test if data fitted a normal distribution, Mann–Whitney test if data did not fit a normal distribution. (**S**) Colon length of *Rorc*^*+/-*^ or WT mice treated with ketoconazole (Keto) or vehicle control (Ctrl) (WT-Ctrl, *n* = 7; WT-Keto, *n* = 7; RORγt-HT-Ctrl, *n* = 8; RORγt-HT-Keto, *n* = 8). Two-tailed unpaired Student’s *t* test. Data information: in (**B**, **C**, **E**, **F**, **K**–**O**, **Q**–**S**), data are presented as mean ± SEM. *P* levels < 0.05*, < 0.01**, < 0.001***, < 0.0001****. Source data are available online for this figure.

We hypothesized that Cyp51, the immediately upstream enzyme of the enriched cholesterol precursors, might be an intervention target to inhibit production of the distal cholesterol precursors meanwhile preserve the proximal precursors that have other biological functions (Mullen et al, 2016). Cyp51 high expression is associated with poor prognosis of CRC (Kumarakulasingham et al, 2005), further supporting the choice of targeting this enzyme. Ketoconazole, a Cyp51 inhibitor clinically used for treating fungi infection (Strushkevich et al, 2010), reduced secretion of distal cholesterol precursors of the MSS-CRC CT26 and Caco2 cells without affecting cell survival in vitro (Appendix Fig. S6). In vivo, ketoconazole suppressed orthotopic CT26 tumor growth in the immunocompetent BALB/c mice, but this effect was minimal in the lymphocyte-deficient B-NDG mice (Fig. 5A–C). Similar results were observed in the subcutaneous CT26 tumor models (Fig. 5D–F). Thus, the anti-CRC effect of ketoconazole was not through the inhibition of tumor cells directly but rather dependent on the modulation of lymphocytes.

We next studied the in vivo effect of ketoconazole in an AOM/DSS induced CRC model using the C57BL/6 mice (Fig. 5G,H). Similar to the human MSS CRC tumors, the mouse AOM/DSS CRC tumors displayed an asynchronous upregulation pattern of cholesterol biosynthesis (Fig. 5I,J) and had accumulation of distal cholesterol precursors (Fig. 5K). Ketoconazole was admitted to mice after the third-round of DSS treatment. Tumor burden was evidently reduced in ketoconazole-treated mice while colon length was not significantly affected (Fig. 5L,M). Th17 cell population in colon was reduced upon ketoconazole treatment (Fig. 5N), along with reduced accumulation of intratumoral cholesterol precursors (Fig. 5K). Th1 cells were nearly unaffected (Fig. 5N). Meanwhile in the draining mesenteric lymph nodes, Th17 population was not affected (Fig. 5O), suggesting that ketoconazole treatment at this dosage did not have Th17-intrinsic inhibition but rather inhibited the tumor microenvironment to affect tumor-infiltrating Th17. To study whether the anti-CRC effect of ketoconazole is dependent on Th17 modulation, we applied the RORγt partial knockout mice that had systemic reduction of Th17 populations (Fig. 5P,Q). The antitumor effect of ketoconazole was abolished in such a model (Fig. 5R,S), suggesting Th17 reduction could be the major mechanism of ketoconazole treatment.

**Figure 6:**
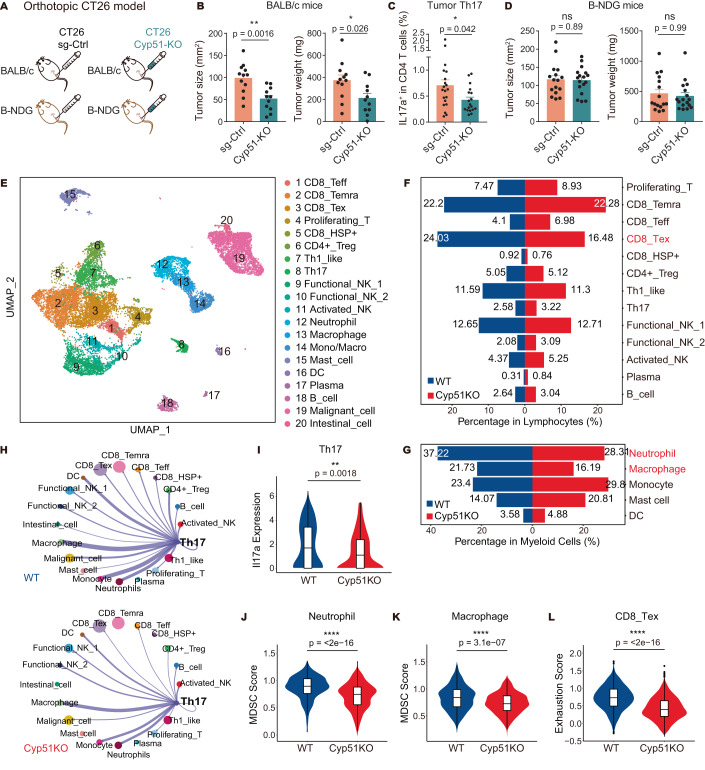
Blocking DCP production of tumor cells suppressed MSS CRC progression and reshaped tumor immune microenvironment. (**A**) Schematic illustration of the orthotopic CT26 model with Cyp51 knockout. (**B**) Tumor burden of orthotopic CT26 MSS CRC in BALB/c mice transplanted with Cyp51-KO CT26 cells (Cyp51-KO, *n* = 11) or sg-control CT26 cells (sg-Ctrl, *n* = 12). Two-tailed unpaired Student’s *t* test. Data are representative of two independent experiments. (**C**) Th17 population in the tumors of BALB/c mice orthotopically transplanted with Cyp51-KO CT26 cells (Cyp51-KO, *n* = 18) or sg-control CT26 cells (sg-Ctrl, *n* = 20). Data pooled from two independent experiments. Mann–Whitney test. (**D**) Tumor burden of orthotopic CT26 MSS CRC in B-NDG mice transplanted with Cyp51-KO CT26 cells (Cyp51-KO, *n* = 17) or sg-control CT26 cells (sg-Ctrl, *n* = 16). Data pooled from two independent experiments. Two-tailed unpaired Student’s *t* test (tumor size), Mann–Whitney test (tumor weight). (**E**–**L**) scRNA-Seq analysis of the orthotopic CT26 tumors in BALB/c mice transplanted with Cyp51-KO CT26 cells (labeled as Cyp51KO) or sg-control CT26 cells (labeled as WT). (**E**) Uniform manifold approximation and projection (UMAP) plot of 17,674 cells from Cyp51KO and WT tumors, showing the formation of 20 main clusters. Each dot corresponds to a single cell and color represent different cell populations. NK natural killer cells, Teff effector T cells, Temra recently activated effector memory or effector T cells, Tex exhausted T cells, Treg regulatory T cells, DC dendritic cells. (**F**) Effects of Cyp51-KO on the tumor-infiltrating lymphocyte compartments. (**G**) Effects of Cyp51-KO on the tumor-infiltrating myeloid cell compartments. (**H**) Circos plots showing cell-cell interaction networks which were sourced from Th17 cluster, line width indicates the number of interacting cytokine-receptor pairs between linked cell clusters. (**I**) Violin plot showing the expression level of *Il17a* in Th17 cells (WT, *n* = 134; Cyp51KO, *n* = 192 cells). (**J**) Violin plot showing the MDSC score in neutrophiles (WT, *n* = 447; Cyp51KO, *n* = 551 cells). (**K**) Violin plot showing the MDSC score in macrophages (WT, *n* = 261; Cyp51KO, *n* = 315 cells). (**L**) Violin plot showing the exhaustion score in exhausted CD8^+^ T cells (WT, *n* = 1248; Cyp51KO, *n* = 982 cells). Data information: in (**B**–**D**), data are presented as mean ± SEM. In (**I**–**L**), box indicates the interquartile range (25–75%); center line indicates the median; Wilcoxon rank-sum test. *P* levels < 0.05*, < 0.01**, < 0.0001****. Source data are available online for this figure.

To explicitly show the effect of tumor-specific Cyp51 inhibition on MSS CRC progression, we studied the in vivo growth of the abovementioned Cyp51-KO CT26 cells that had reduced secretion of distal cholesterol precursors and impaired induction of Th17 polarization (Fig. 4C–F). Like ketoconazole treatment, Cyp51 knockout significantly suppressed CT26 tumor progression in the immunocompetent BALB/c mice (Fig. 6A,B), along with reduction of tumor-infiltrating Th17 population (Fig. 6C). On the other hand, the growth difference between Cyp51-KO and wildtype CT26 tumors was minimal in the immunocompromised B-NDG mice that have lymphocyte-deficiency (Fig. 6D). Thus, the anti-CRC effect of Cyp51 intervention was not through the direct inhibition of tumor cells but rather through the modulation of lymphocytes.

**Figure EV5:**
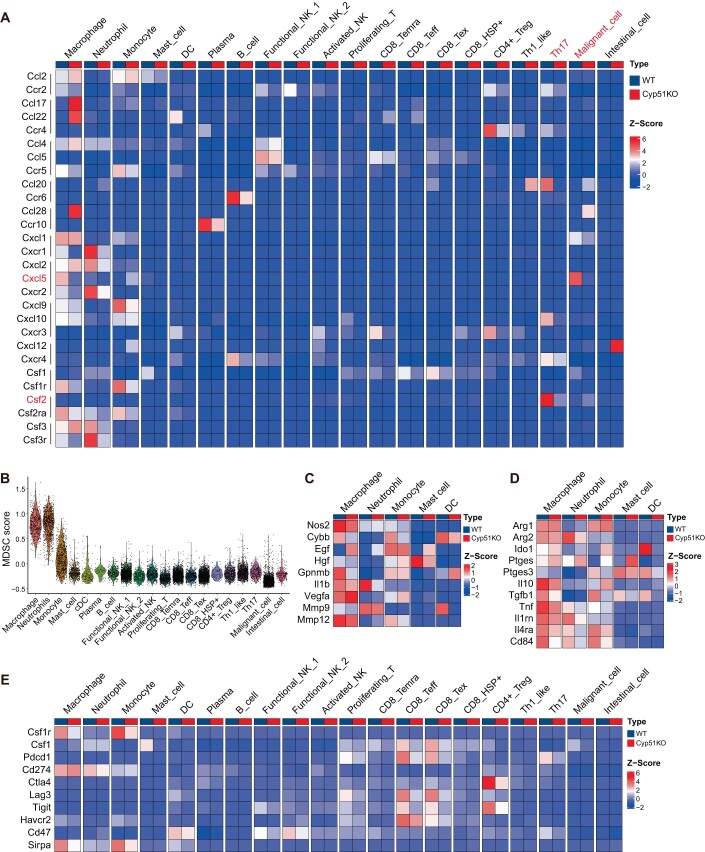
Single-cell analysis of CT26 tumors (WT or Cyp51KO) in BALB/c mice as described in Fig. 6. (**A**) Heatmap showing the gene expression levels of chemokines/growth factors and related receptors across all cell clusters. *Z*-score was calculated by mean expression level and indicated by color. (**B**) Violin plot showing the MDSC score in all cell clusters. The neutrophils and macrophages are major contributor of the suppressive function of myeloid derived suppressive cells (MDSC) (Dysthe and Parihar, 2020) in tumor. (**C**) Heatmap showing the expression levels of tumor growth/metastasis related genes across myeloid cell populations. *Z*-score was calculated by mean expression level and indicated by color. (**D**) Heatmap showing the expression levels of immunosuppression related genes across myeloid cell populations. *Z*-score was calculated by mean expression level and indicated by color. (**E**) Heatmap showing the expression levels of immune checkpoint genes across all cell populations. *Z*-score was calculated by mean expression level and indicated by color.

Single-cell RNA sequencing was performed to better understand how Cyp51 knockout in tumor cells impacted tumor immune microenvironment. Unsupervised graph-based clustering was used to identify 13 clusters of lymphocytes, 5 clusters of myeloid cells, and 2 clusters of epithelial cells (Fig. 6E–G, Appendix Fig. S7, Dataset EV1). Th17 cells had reduced *IL17a* expression and weakened interactions with other cell populations in Cyp51-KO tumors (Fig. 6H,I), including the axis of Th17- neutrophils/macrophages and Th17-tumor cells. GM-CSF (*Csf2*), a growth factor affecting recruitment and maturation of neutrophils/macrophages, driven by RORγt in Th17 (Codarri et al, 2011), was reduced in Th17 cells of Cyp51-KO tumors (Figure EV5A). Another neutrophil-recruiting chemokine Cxcl5, which is a downstream molecule of IL-17 signaling (Liu et al, 2011), dropped in tumor cells after Cyp51 knockout (Figure EV5A). In line with these findings, neutrophils and macrophages had decreased cell proportions (Fig. 6G) and attenuated protumor and immunosuppression gene signatures (Dysthe and Parihar, 2020) in Cyp51-KO tumors (Figs. 6J,K and  EV5B–D, Dataset EV2). Consistently, CD8^+^ T cells had less exhaustion (Fig. 6F,L), and immune checkpoint expressions were generally downregulated in different immune compartments (Figure EV5E). Altogether, the intervention of tumor cholesterol biosynthesis reshaped the MSS CRC immune landscape to suppress tumor growth.

## Discussion

As fast proliferating cells, cancer cells usually upregulate the cholesterol biosynthesis pathway to produce cholesterol for membrane biogenesis. Intervention of cholesterol biosynthesis has been tested in various cancer types (Huang et al, 2020; Silvente-Poirot and Poirot, 2014). The roles of cholesterol precursors in tumor are less understood. In anaplastic large cell lymphoma cells, loss of SQLE expression causes accumulation of squalene that can protect cells against ferroptosis by maintaining the adequate composition of membrane polyunsaturated fatty acids (Garcia-Bermudez et al, 2019). However, whether cancer cells could secret cholesterol precursors to modulate immune landscape has not been reported before. Here we observed MSS CRC cells have an asynchronous pathway of cholesterol biosynthesis featured with significant upregulation of most distal enzymes but nearly no change of the last enzyme DHCR24, thus favoring accumulation of distal cholesterol precursors to polarize Th17. It has been well recognized that cholesterol biosynthesis is controlled by SREBP2 (Luo et al, 2020). However, to achieve proper transcriptional regulation, other transcription factors are also required to fine tune the transcriptions of SREBP2-sensitive genes (Villagra et al, 2007). Here we find that MYC is upregulated in MSS CRC and has differential regulations on cholesterol biosynthesis enzymes, which could provide a transcriptional basis for the asynchronous pattern of cholesterol biosynthesis.

The aberrant cholesterol metabolism of MSS CRC cells caused metabolic changes not only in tumor microenvironment but seemly also in blood. The reduction of desmosterol levels in plasma of post-surgery patients suggests that desmosterol might serve as a blood biomarker of MSS CRC. In fact, desmosterol has been proposed as a plasma biomarker for Alzheimer’s disease (downregulation) (Sato et al, 2012) and non-alcoholic steatohepatitis (upregulation) (Simonen et al, 2013), reflecting the importance of distal cholesterol precursors in human health and disease.

Unlike MSS CRC with Th17 enrichment, the MSI CRC do not have significant enrichment of Th17 (Pelka et al, 2021; Zhang et al, 2018). Here in our analysis of COAD data, the MSI-H CRC tumors showed less active cholesterol biosynthesis when compared with the MSS CRC tumors. At cell line level, the significant enrichment of distal cholesterol precursors in conditioned media of MSS-CRC cells but not MSI-CRC cells echoed with the COAD data, indicating the different cholesterol metabolic pattern of the different CRC subtypes, which provided a metabolic explanation for the different immune landscapes. In this study, we did not collect enough MSI CRC clinical samples for reliable sterol profiling analysis due to the low prevalence of MSI CRC. In further studies, more detailed analysis of MSI CRC and also distant metastasis CRC tumors are needed to have a comprehensive understanding of CRC metabolic feature and tumor immune phenotype.

Based on the fast-increasing body of clinical data, the roles of proinflammatory microenvironment in cancer progression and drug resistance starts to be appreciated. Intervention strategies of reshaping immune landscape need to be developed based on the understanding of the characteristics of a specific cancer type. For MSS CRC, its unique cholesterol biosynthesis flux can be intervened to reduce Th17 infiltration and in turn inhibit tumor progression. This strategy might be also relevant for treating other cancers in which IL-17 plays tumor-promoting roles (Zhao et al, 2020).

## Methods

### Reagents and mice

In T cell activation and differentiation experiments, anti-mouse CD3ε (145-2C11) and anti-mouse CD28 (37.51), anti-mouse IFNγ (R4-6A2), anti-mouse IL-4 (11B11) were from Biolegend; IL-1β, IL-2, IL-6, IL-12, IL-21, IL-23, TGF-β were from PeproTech.

In flow cytometric analysis, anti-mouse CD4 (RM4-5), anti-mouse CD8 (53–6.7), anti-mouse CD45 (30-F11), anti-mouse IL-17a (eBio17B7), anti-mouse IFNγ (XMG1.2), anti-mouse Granzyme B (NGZB), anti-mouse CD44 (IM7), anti-mouse FoxP3 (FJK-16s) were from eBioscience, and 1:200 diluted for staining. LIVE/DEAD Fixable Dead Cell Stain Dye-Aqua was from Invitrogen.

For IP and immunoblotting, anti-DHCR24 (10471-1-AP), anti-CYP51A1 (13431-1-AP) were from Proteintech, and 1:1000 diluted for blotting; anti-c-Myc (E5Q6W), anti-rabbit IgG-HRP-linked antibody (7074) and normal rabbit IgG (2729) were from CST.

For ELISA quantification, IL-6, IL-23, TGF-β, IL-1 β and IL-21 ELISA Kit were from Invitrogen.

Desmosterol was from Avanti Polar Lipids. Ketoconazole and Myci975 were from Selleck.

CT26 (RRID: CVCL_7256), HCT116 (RRID: CVCL_0291) and Caco-2 (RRID: CVCL_0025) cell lines were from the Cell bank of the Chinese Academy of Sciences. MC38 was from Wei Yang, Southern Medical University. Cells were tested negative for mycoplasma contamination.

C57BL/6 and BALB/c mice were from SLAC (Shanghai, China). The Immune-deficient B-NDG mouse (NOD.CB17-*Prkdc*^*scid*^*Il2rg*^*tm*1^/Bcgen) were from Biocytogen. LDLR-KO (RRID: IMSR_JAX:002207) and wildtype control mice were from Changzhou Cavens Lab Animal Co., Ltd. RORγt-KO (RRID: IMSR_JAX:007572) mice were from Youchun Qian, Institute of Health Sciences, Chinese Academy of Sciences.

All mice were maintained in the pathogen-free facilities at the Institute of Biochemistry and Cell Biology. Animals were randomly allocated to experimental groups. All animal experiments used mice with matched age and sex. The animal experiments were performed in a double blinded manner. All animal experiments were approved by the Institutional Animal Care and Use Committee (IACUC) of Shanghai Institute of Biochemistry and Cell Biology, Chinese Academy of Sciences and complied with all relevant ethical regulations.

### Human sample collection

The eligible criteria were set as follows: (1) participants were not receiving any medical treatment before surgery; (2) everyone was not diagnosed with any metabolic diseases, such as kidney diseases, liver diseases or others; (3) patients were diagnosed with CRC by biopsy before surgery. All patients were staged according to the Union for International Cancer Control (UICC) pathologic tumor-node-metastasis (TNM) classification system (eighth edition, 2016). Consent was obtained from all human subjects and the experiments were conformed to the principles set out in the WMA Declaration of Helsinki and the Department of Health and Human Services Belmont Report.

Whole tissue samples of CRC tumor and paired adjacent normal tissues were from Harbin Medical University Cancer Hospital (Harbin, China). Tissues were immediately frozen in liquid nitrogen after surgery and then stored at −80 °C refrigerator. Detail information had been described in previous publication(Wang et al, 2019). Informed consent was obtained from each patient, and the study protocol has been approved by the Ethics Committee of Harbin Medical University Cancer Hospital.

Plasma samples of CRC patients before and after surgical resection of tumors were from Harbin Medical University Cancer Hospital (Harbin, China). Vacuum blood collection tube (5 ml) with anticoagulant Dipotassium EDTA was used for collecting overnight fasting blood samples (during 6:30–6:45 a.m. on the day before surgery for pre-operative plasma samples, and on the 7th day after surgery for post-operative plasma samples). The fresh blood was centrifuged at 1300 rpm for 10 min, and then supernatant was extracted and immediately frozen at −80 °C refrigerator. Informed consent was obtained from each patient, and the study protocol has been approved by the Ethics Committee of Harbin Medical University Cancer Hospital.

Interstitial fluid samples of CRC tumor and paired adjacent normal tissues were from Shanghai Jiao Tong University Affiliated Sixth People’s Hospital (Shanghai, China). Tumors and paired adjacent normal tissues were freshly isolated and interstitial fluid were isolated as described previously (Ho et al, 2015). Informed consent was obtained from each patient, and the study protocol has been approved by the Ethics Committee of Shanghai Jiao Tong University Affiliated Sixth People’s Hospital.

### Immunohistochemistry for the detection of MMR proteins

The immunohistochemistry for MMR proteins (MLH1, MSH2, MSH6, and PMS2) was assessed. Anti-hMLH1 (IR079, DAKO, Agilent, America), anti-hMSH2 (IR085, DAKO, Agilent, America), anti-hMSH6 (IR086, DAKO, Agilent, America) and anti-hPMS2 (IR087, DAKO, Agilent, America) were ready-to-use working fluids. Microsatellite stability was defined as positive expression of all four proteins, and the loss expression of any one protein is defined as microsatellite instability.

### Genetic manipulation of cholesterol biosynthesis genes

To generate knockout constructs, sgRNA was inserted into LentiCRISPRv2-TagBFP vector. TagBFP was used as sorting signal. The sequences of sgRNAs were (5′-3′):

Mouse *Cyp51* sgRNA: CAAGACCTTCACTTACCTTC;

Mouse *Dhcr24* sgRNA: GAGTCATCGTCCCACAAGTA;

Mouse sg control: TGCAACGATGGTTACGGTAC;

To overexpress *Dhcr24* in CT26 cells, mouse *Dhcr24* gene was amplified from mouse colon cDNA library and subsequently cloned into PHAGE expression vectors for ectopic expression.

To recapitulate the asynchronous upregulated cholesterol biosynthesis pattern of MSS-CRC in MC38 cells, a truncated version of NH2-terminal (1-457) DNA-binding domain of mouse Srebp2 that terminated prior to the membrane attachment domain and enter the nucleus directly bypassing the sterol-regulated cleavage step (*nSrebp2*) and mouse *Dhcr24* gene was amplified from mouse liver/colon cDNA library and subsequently cloned into PHAGE expression vectors with EF1α and PGK promoter respectively for ectopic expression. ZsGreen and mCherry were used as sorting signals respectively. Endogenous Dhcr24 of MC38 cells was knocked-out as described above, followed by a stable transfection of PHAGE-PGK-Dhcr24 plasmid so that its Dhcr24 expression is no longer activated by Srebp. These cells were also transfected with the above-mentioned nSrebp2 plasmid. With careful titration, this MC38 cell line got 2–4-fold increased expression of cholesterol synthesis genes without strongly affecting the expression of Dhcr24.

All constructs were confirmed by sequencing. Packaging plasmids for lentiviruses psPAX2 and PMD.2G were purchased from Addgene. 293FT cells were transfected with the packaging plasmids (psPAX2 and PMD.2G) and the lentiCRISPRv.2-TagBFP plasmid (knockout) or PHAGE plasmid (ectopic expression) by Lipofectamine 2000 (Invitrogen). Virus-containing supernatants were collected 48 h after transfection. Target cells were cultured with the virus supernatant for 48 h. TagBFP/ZsGreen/mCherry positive cells were sorted for subsequent validation.

### Quantitative reverse transcription PCR

Total RNA was extracted with Trizol (Life technology) from the indicated cells or tissues and subjected to quantitative reverse transcription PCR (qRT–PCR) using gene specific primers (5′–3′):

*Sqle* (forward, ATAAGAAATGCGGGGATGTCAC; reverse, ATATCCGAGAAGGCAGCGAAC)

*Cyp51* (forward, GACAGGAGGCAACTTGCTTTC; reverse, GTGGACTTTTCGCTCCAGC)

*Dhcr24* (forward, CTCTGGGTGCGAGTGAAGG; reverse, TTCCCGGACCTGTTTCTGGAT)

### ChIP-qPCR

CT26 cells with 80%–90% confluency were harvested and washed with PBS. Cells were crosslinked in 1% formaldehyde (Sigma) with rotation in room temperature for 10 min, then neutralized with 300 mM glycine. Cells were pelleted and washed twice with ice cold PBS. 1.2 × 10^7^ CT26 cells were resuspended in sonication buffer (0.1% SDS, 1% triton X-100, 10 mM pH 7.4 Tris-HCl, 1 mM pH 8.0 EDTA, 0.1% NaDOC, 0.25% Sarkosyl, 1 mM DTT) supplemented with protease inhibitor cocktail (Sigma, 1:200), and sonicated to shear the genomic DNA to 300–500 bp (on ice). DNA sizes were validated by gel electrophoresis. Lysate was centrifuged for 10 min at 13,000 g at 4 °C and supernatant was transferred to a new tube. NaCl was added to supernatant to 300 mM for immunoprecipitation. 10 μl (1/100 from total volume) of sheared chromatin was saved as input control, and the rest was split into two vials. The c-Myc antibody (CTS, 1:50) or rabbit IgG (CST, 1:50) was added to each vial and IP overnight with rotation at 4 °C. The next day, prewashed Dynabeads Protein G (Invitrogen, 10004D) (50 μl beads/10 μl antibody) was added to each IP sample and continued to rotate for 4 h at 4 °C to pull down the antibody/chromatin complex. Beads were harvest (on ice) with magnet and supernatant was discarded by pipetting. Beads were washed with the four buffers in order (each buffer for two times): 1 ml RIPA 0.3 buffer (0.1% SDS, 1% Triton X-100, 10 mM pH7.4 Tris-HCl, 1 mM pH 8.0 EDTA, 0.1% NaDOC, 0.3 M NaCl), 1 ml high salt buffer (0.1% NaDOC, 1% Triton X-100, 1 mM pH 8.0 EDTA, 50 mM pH 7.5 Tris-HCl, 500 mM NaCl), 1 ml LiCl buffer (250 mM LiCl, 0.5% NP-40, 0.5% NaDOC, 1 mM pH 8.0 EDTA, 10 mM pH 8.1 Tris-HCl), 1 ml TE buffer (10 mM pH 7.5 Tris-HCl, 1 mM pH 8.0 EDTA). After wash, 100 μl elution buffer (1% SDS, 10 mM pH 8.0 EDTA, 50 mM pH 8.1 Tris-HCl) was added to the beads and incubated at 65 °C overnight for reversing crosslinking. Then, beads were collected by magnet and supernatant was transferred to a new tube. 5 μl 10 mg/ml RNase A (Thermo) was added to the supernatant and incubated at 37 °C for 30 min, followed with the addition of 5 μl 20 mg/ml Protease K (Thermo) and incubation at 65 °C for 2 h. The preserved input fraction was also subjected to the treatment of elution buffer for reversing crosslinking, and the treatments of RNase A and Protease K for removing RNA and protein.

ChIP DNA was purified by TIANquick midi purification kit (TIANGEN) and eluted with 40 μl ddH2O. For each qPCR experiment, 1 μl eluent was used. ChIP-qPCR primers were designed according to the published c-Myc ChIP-seq data deposited at Cistrome DB (http://cistrome.org/db/#/) and their binding sites were predicted at Jaspar browser (https://jaspar.genereg.net/). All primers were searched for potential off-target by the NCBI primer BLAST, and tested by qPCR and electrophoresis for specificity. The selected ChIP primers are (5′–3′):

Mouse *Cyp51* forward: AGCTCTGCTGACGCCACATAGGC;

Mouse *Cyp51* reverse: GGATCTCAGGACCAGATTGGTGG;

Mouse *Sqle* forward: TCTGAAGGACGCTCATCCGAGACA;

Mouse *Sqle* reverse: TTGGAGATTCCTCCTCAAGCAA;

Mouse *Dhcr24* forward: AAGAATGCCATCAACGCCTCTGT;

Mouse *Dhcr24* reverse: TTAGCAGTTCTGGGCCGTGG

In the qPCR experiments, the Ct values of the c-Myc or IgG IP samples were first normalized to input. Then, the c-Myc samples were normalized to the IgG samples.

### Isolation, differentiation and function analysis of mouse T cells

Peripheral naive CD4^+^ and CD8^+^ cells were isolated from mouse spleens or lymph nodes by negative selection (STEM CELL Technologies). To isolate the colon/tumor-infiltrating T cells, colon/tumor tissues were mechanically disrupted and digested with collagenase I and IV (sigma) and DNase I(TIANGEN). Disruption was performed with 1× MACS program m_impTumor_03 using the gentleMACS Dissociator (Miltenyi Biotec) before and after incubation at 37 °C for 1 h. After disruption, cells were passed through a 70 mm filter and washed. Leukocytes were further enriched by 40–70% Percoll (GE) gradient centrifugation.

For Th1 differentiation, naive CD4^+^ T cells were stimulated with 5 μg ml^−1^ plate-bound anti-CD3 and anti-CD28 in the presence of 10 ng ml^−1^ IL-2, 10 ng ml^−1^ IL-12 and 10 μg ml^−1^ anti-IL-4 for 4 days. For Th17 differentiation, naive CD4^+^ T cells were stimulated with 5 μg ml^−1^ plate-bound anti-CD3 and anti-CD28 in the presence of 30 ng ml^−1^ IL-6, 20 ng ml^−1^ IL-23, 5 ng ml^−1^ TGFβ, 10 ng ml^−1^ IL-1 β, 50 ng ml^−1^ IL-21, 10 μg ml^−1^ anti-IFNγ and 10 μg ml^−1^ anti-IL-4 for 4 days. Ursolic acid (0–4 μM) was added on day 0 to lower RORγt basal activity, according to the Th17 polarization efficiency. For Treg differentiation, naive CD4^+^ T cells were stimulated with 5 μg ml^−1^ plate-bound anti-CD3 and anti-CD28 in the presence of 10 ng ml^−1^ IL-2 and 1 ng ml^−1^ TGF-β for 4 days. For intracellular staining, cells were stimulated with 1 μM ionomycin and 50 ng ml^−1^ phorbol 12-myristate 13-acetate (PMA) for 4 h in the presence of 5 μg ml^−1^ BFA before harvest. For colon/tumor-infiltrating T cell analysis, animals were excluded from the analysis when number of total isolated CD4^+^ T cells was less than 1000.

To measure cytokine production of CD8^+^ T cells, naive CD8 T cells were stimulated with 5 μg ml^−1^ plate-bound anti-CD3 and anti-CD28 for 2 days. 5 μg ml^−1^ BFA was added 4 h before harvest for intracellular staining. To measure CD44 level of CD8^+^ T cells, naive CD8^+^ T cells were stimulated with 5 μg ml^−1^ plate-bound anti-CD3 and anti-CD28 for 1 day before surface staining.

### Conditioned media

For conditioned media generation, 1 × 10^6^ cells were cultured for 2–3 days till grown to 100% confluence. For parallel experiment, same number of cells were seeded. Blank control media were cultured for the same time with no cells seeded in. The conditioned media were then collected and prepared for mass spectrometry measurement. For T cell experiments, conditioned media were concentrated with 3k MWCO Protein Concentrator (Thermo Scientific) for five folds (v/v) before added into T cell culture system. Lipoproteins were isolated from conditioned media as described (Loregger et al, 2017), and concentrated for five folds (v/v) before added into T cell culture system.

### Measurement of cell viability

Cells (2 × 10^3^) in 100 μl media were cultured for 72 h. 20 μl MTS reagent (CellTiter 96® AQueous One Solution Cell Proliferation Assay, Promega) was added into each well. After 2–3-h incubation, the absorbance at 490 nm was measured. The effect of Ketoconazole on cell viability was obtained by normalizing the absorbance of drug-treated cells with that of the vehicle-treated cells.

### Mouse CRC model

For AOM/DSS CRC model, C57BL/6 wide type or *Rorc*^*+/−*^ male mice aged at 8 to 10 weeks were injected intraperitoneally with 10 mg kg^−1^ body weight azoxymethane (AOM, Sigma) in sterile isotonic saline. The mice were then fed with 2.5% (wt/vol) inflammatory agent dextran sodium sulfate (DSS, Sigma) in drinking water for 1 week and followed with normal water for 3 weeks. The DSS-normal water cycles were repeated 3 times in total. For ketoconazole treatment experiment, after the third round of DSS administration, mice were randomly divided in different groups and ketoconazole or vehicle control was delivered every other day at the dose of 20 mg kg^−1^ by intragastric administration. Mice were sacrificed on week 16 for tumor development analysis and T lymphocytes analysis. Tumor loads were identified by count tumor numbers and measure tumor size (diameter) with sliding calliper.

For CT26 transplant CRC model, BALB/c male mice or B-NDG female mice aged at 8 to 10 weeks were injected orthotopically or subcutaneously with 3 × 10^5^ CT26 cells as described (de Sousa e Melo et al, 2017). For ketoconazole treatment experiment, mice were randomly divided in different groups on day 7. Ketoconazole or vehicle control was delivered every other day at the dose of 20 mg kg^−1^ by intragastric administration since day 8. For subcutaneous tumor model, the tumor size was measured every two or three days, and calculated as length × width. Mice with subcutaneous tumor size larger than 15 mm at the longest axis were euthanized for ethical consideration. For orthotopic tumor model, mice were sacrificed on day 21 for tumor development analysis and T lymphocytes analysis.

### LC-MS-based sterol analysis

The sterol analysis followed the methods reported previously (Qiu et al, 2021).

#### Sterol extraction

Human CRC tumor or adjacent normal tissues were weighted and homogenized in H_2_O (~200 μl H_2_O for ~10 mg tissue) using the homogenizer (Precellys 24, Bertin Technologies, France). The homogenizer was equipped with a cold trap filled with liquid nitrogen to maintain low temperature during homogenization. Then, 50 μl of homogenized solution was taken and diluted to 100 μl using water. Then, 400 μl of the extraction solvent (dichloromethane (DCM)/methanol (MeOH) (2:1; v/v) containing 6.5 μg butylated hydroxytoluene (BHT)) was added. The solution was vortexed for 30 s, followed by 10 min of sonication, and centrifugation at 3000 rpm for 15 min at 4 °C. The bottom organic layer was collected (200 μl). And an additional 200 μl of DCM was added to the rest layer for re-extraction. The re-extraction was repeated twice. The pooled organic layer was evaporated using a vacuum concentrator at 4 °C (LABCONCO, USA).

The cell conditioned medium (200 µl) or CRC interstitial fluid (200 µl) was first mixed with 800 μl of the extraction solvent (DCM/MeOH (2:1; v/v) containing 6.5 μg BHT). The solution was vortexed for 30 s, followed by 10 min of sonication, and centrifugation at 3000 rpm at 4 °C for 15 min. The bottom organic layer was collected (400 μl). And an additional 400 μl of DCM was added to the rest layer for re-extraction. The re-extraction was repeated twice. The pooled organic layer was evaporated as described above.

For cell samples, 10^6^ cells per sample were suspended with 250 μl of H_2_O, and then frozen with liquid nitrogen and thaw at room temperature. The freeze-thaw cycle was repeated for three times. Then, 200 μl of cell lysates was used to sterol extraction. The cell lysates (200 µl) were first mixed with 800 μl of the extraction solvent DCM/MeOH (2:1; v/v) containing 6.5 μg BHT. The solution was vortexed for 30 s, followed by 10 min of sonication, and centrifugation at 3000 rpm at 4 °C for 15 min. The bottom organic layer was collected (400 μl). And an additional 400 μl of DCM was added to the rest layer for re-extraction. The re-extraction was repeated twice. The pooled organic layer was evaporated using a vacuum concentrator at 4 °C.

#### Sample saponification

The sample saponification was performed as the described method (Honda et al, 2009; Honda et al, 2008) with minor modifications. The dry extract was mixed with 500 µl of 1 M methanolic KOH, vortexed for 30 s, sonicated for 10 min, and incubated at 37 °C for 1 h. Then 1 ml hexane was added for extraction. The sample was vortexed for 30 s, and sonicated for 10 min in a 4 °C water bath, centrifuged for 15 min at 3000 rpm at 4 °C. The supernatant was collected (600 μl). The hexane extraction step was repeated once, and the supernatant was collected (1000 μl) and pooled with the previous supernatant. Finally, 1.6 ml of supernatant was evaporated to dryness using a vacuum concentrator at 4 °C.

#### Sample derivatization

Sterol derivatization to the picolinyl ester was performed according to the previous method (Honda et al, 2009; Honda et al, 2008) with minor modifications. The chemicals for derivatization including picolinic acid (53.3 mg), 2-methyl-6-nitrobenzoic anhydride (66.7 mg), and 4-dimethylaminopyridine (3 mg) were sequentially added to pyridine (1 ml). The freshly prepared derivatization reagent (200 μl) and 5.36 μl triethylamine were added to the dry extract. The reaction mixture was vortexed for 30 s, sonicated for 10 min, and incubated at 80 °C for 60 min. Then, 1 ml of hexane was added to samples for extraction. The samples were again vortexed for 30 s, sonicated for 10 min, and centrifuged for 15 min at 3000 rpm at 4 °C. Finally, supernatant was collected and evaporated to dryness using a vacuum concentrator at 4 °C.

#### Sample cleaning

The dry extract after derivatization was mixed with 400 μl DCM and 600 μl H_2_O for sample cleaning. Then the solution was vortexed for 30 s, sonicated for 10 min in a 4 °C water bath, and centrifuged for 15 min at 3000 rpm at 4 °C. Next, 400 μl of upper aqueous layer was removed. Then, another 400 μl H_2_O was added into the rest sample solution for cleaning. The cleaning step was repeated twice. The rest organic layer was collected and evaporated to dryness using a vacuum concentrator at 4 °C. The dry extract was kept at −80 °C and reconstituted using 100 μl ACN prior to LC–MS analysis. For cholesterol analysis, the samples were diluted 50 times using ACN before analysis.

### LC-MS analysis and data processing

The LC-MS analyses were performed using a UHPLC system (1290 series, Agilent Technologies) coupled to a quadrupole time-of-flight (Q-TOF) mass spectrometer (Agilent 6550 iFunnel Q-TOF, Agilent Technologies, USA). Chromatographic separations were performed on a Phenomenex Kinetex C18 column (particle size, 1.7 μm; 100 mm (length) × 2.1 mm (i.d.)) with a column temperature of 50 °C. The mobile phases (A, water with 0.1% acetic acid; B, methanol with 0.1% acetic acid) were used for separation. The linear gradient eluted from 0 to 25% B (0–3 min, 0.4 ml min^−1^), from 25 to 85% B (3–5 min, 0.4 ml min^−1^), 85% B (5–5.1 min, 0.4 to 0.6 ml min^−1^), from 85 to 93% B (5.1–14 min, 0.6 ml min^−1^), 93% B (14–17 min, 0.6 ml min^−1^), from 93 to 100% B (17–17.2 min, 0.6 ml min^−1^), 100% B (17.2–19 min, 0.6 ml min^−1^), from 100 to 0% B (19–19.1 min, 0.6 ml min^−1^), 0% B (19.1–19.2 min, 0.6 to 0.4 ml min^−1^), 0% B (19.2–20 min, 0.4 ml min^−1^). The first 3-min LC flow was set to waste. The MS parameters included as the follows: ESI positive mode; mass range, 124–1300 Da; dry gas temperature, 250 °C; dry gas flow, 16 l min^−1^; sheath gas temperature, 350 °C; sheath gas flow, 12 l min^−1^; nozzle voltage, 0 V; nebulizer pressure, 20 psig; capillary voltage, 3000 V. The all ion fragment (AIF) method was used for data acquisition. The acquisition cycle time was 500 ms, including 250 ms for MS1 scan, and 250 ms for MS2 scan. The collision energy (CE) was set as 0 V for MS1 scan and 20 V for MS2 scan.

The qualitative analyses were performed using Agilent MassHunter Qualitative Analysis Software (version B.07.00) and an in-house PCDL sterol library including a total of 34 sterol chemical standards. The sterol library included formula, exact mass, experimental retention time (RT), and experimental MS/MS spectrum for each sterol. The raw MS data files were processed using “Find Compounds by Formula” function in MassHunter. To do so, the in-house PCDL sterol library was first loaded. The “formula matching” parameters were set as: match tolerance: masses, 25 ppm; retention time, 0.5 min. The “Fragment confirmation” parameters were set as: RT difference, 0.1 min; S/N ratio ≥3.00; Coelution score ≥50; minimum number of qualified fragments, 1. After the data processing, identification results with overall score ≥60 were reserved. Manual verification was further performed to inspect the identification results. Finally, all sterol identifications were validated through the comparison with chemical standards.

The relative quantification analyses were performed by Skyline (version 4.2.0.19009, 64-bit). The transition list was imported into Skyline, including *m*/*z* of precursor and experimental RT obtained in qualitative analysis. The extracted ion chromatographic (EIC) peak of each sterol in the transition list was extracted. Manual integration of the peak area was performed and exported for the followed statistical analyses.

For sample preparation in the absolute quantification of desmosterol, 40 μl interstitial fluid or homogenized solution of whole tissues was spiked with deuterated internal standard (IS) d6-desmosterol (10 ng) before extraction. The mixture was diluted to 200 μl using water for extraction. The sample preparation and sterol identification followed the same procedures as described before. Chromatographic peak areas of desmosterol and d6-desmosterol in biological samples were exported. The calibration samples were prepared with different concentrations desmosterol and d6-desmosterol (10 ng/ml) and ran two times in the beginning and end of the analysis batch. Peak area ratios were calculated (desmosterol/IS) to generate the calibration curve. The concentrations of sterols in samples were interpolated from the calibration curve.

### Stable isotope tracing of sterol lipids

The CT26 cells were plated in 6 cm dishes at 100,000 cells/dish, and cultured in RPMI_1640_ medium containing LPDS (1%) and penicillin/streptomycin (1%). The MC38 cells were plated in 6 cm dishes at 100,000 cells/dish, and cultured in DMEM medium containing LPDS (1%) and penicillin/streptomycin (1%). Cells were first grown to 10–20% confluence in log-phase in 6 cm dishes. Then, the culture medium solution was changed to a fresh medium solution with 5 mM ^13^C_2_-acetate. The labeling experiments were performed for 72 hours. Then, the culture medium was quickly removed, and the cells were washed with the PBS. After trypsin digestion, the cells were transferred to a 2-ml Eppendorf tube. The samples were centrifuged for 10 min at 800 rpm and 4 °C. The cell pellet was collected and kept at −80 °C.

### TCGA data analysis

#### Differential gene expression analysis

We obtained normalized gene expression TCGA-COAD data (log2 RPKM from RNA-seq) from National Cancer Institute GDC Data Portal (portal.gdc.cancer.gov). Comparison between CRC MSS-tumor tissues and normal tissues (COAD total, 294 to 41; COAD Stage 1, 48 to 4; COAD Stage 2, 98 to 22; COAD Stage 3, 91 to 7; COAD Stage 4, 47 to 7; COAD female, 121 to 21; COAD male, 172 to 20; COAD MSS-paired, 25 to 25; COAD plus GTEx v8, 294 to 79), as well as MSI-H (89 samples) versus MSS (294 samples) in COAD, were tested using *t* test, and *p* values were adjusted by BH (Benjamini and Hochberg, 1995). Since TCGA and GTEx readouts are not in the same units and have batch effects interfering with data interpretation, we unified the TCGA-COAD and GTEx datasets before GSEA analysis as described previously (Wang et al, 2018).

#### Functional analysis

We used GSEA v3.0 (Broad Institute, PreRanked mode) for enrichment analysis. To be consistent with the way for identifying significant genes, we use the *t*-statistic output from *t* test as the metrics for ranking. 1000 gene set permutations were set as default, and gene sets was obtained through collecting pathways from KEGG with the CholMetab gene set combined.

#### ATAC-seq analysis

We adopted processed genome-wide chromatin accessibility profiles from the publication (Corces et al, 2018). The provided bigWig files of ATAC-seq were generated from 81 primary tumor samples in TCGA-COAD collection. According to the methods (Corces et al, 2018), the provided bigWig files have been normalized by the total insertions in peaks and then binned into 100-bp bins. Each 100-bp bin represents the normalized number of insertions that occurred within the corresponding 100 bp. Using deepTools 3.5.0, visualization of ATAC-seq promoter signals for each individual technical replicate was completed. We defined promoter signals as number of insertions occurring within a window of ±3000 bp flanking a transcriptional start site (TSS). The representative ATAC-seq tracks were indicated separately.

The ATAC-seq data for CT26 cell were obtained from the previous publication (GSE139476). Raw reads were mapped to the mouse genome assembly mm10 by BOWTIE2 (version 2.3.1) with --very-sensitive -X 2000 parameters. Unique mapped reads were deduplicated using Sambamba (version 0.7.1) markdup. Bam files were coverted to bigwig files with RPKM normalizing for visualization by deeptools (version3.5.0.).

#### Motif analysis

We search the motifs at the promoters of upregulated or downregulated cholesterol biosynthesis related genes using PSCAN tool (Zambelli et al, 2009). Briefly, to scan for candidate motifs enriched in the given DNA sequences, the program computes for each input sequences with a motif matching value, and calculate the mean of the matching value on all the input sequences. For each annotated motif, the whole genome promoter set is modeled as background and the mean of the matching score from the input promoter regions is be compared to the mean and the standard deviation of the matching score on background. The enrichment for each motif is finally assessed with a *z*-test, to test the probability of the profile obtaining the same score on a random sequence set.

### Single-cell RNA sequencing data analysis of human CRC tumors

We analyzed the transcription level of cholesterol biosynthesis pathway related genes in single cell RNA-seq data collected from human colorectal tumors (Zhang et al, 2020a). The expression data was downloaded from Gene Expression Omnibus (GEO) via the accession number GSE146771 We calculated the average expression of genes with log-transformed expression profiles.

### RNA sequencing, library generation, and bioinformatics analysis of mouse CRC samples

Mouse CRC paired tumor and colon tissues were isolated from AOM/DSS CRC bearing C57BL/6 mice as described above. Tissues were immediately frozen in liquid nitrogen after isolated and then stored at −80 °C refrigerator.

Total RNA from was isolated with RNeasy Mini Kit (Qiagen) according to the manufacturer’s instructions and RNA integrity was determined using an Agilent Bioanalyzer4200 (Agilent technologies).

VAHTS stranded mRNA-seq Library Prep Kit (NR612, Vazyme) was used to prepare the sequencing library according to the manufacturer’s protocol. The cDNA library was sequenced using the Illumina sequencing platform (Novaseq). The RNA isolation, library construction and sequencing were performed at Shanghai Biochip Co., Ltd. For gene-expression analysis, the fragments of genes were counted and subsequently normalized by FPKM according to the following formula.

$${{{{{{{\mathrm{FPKM}}}}}}}} = \frac{{{{{{{{{\mathrm{Total}}}}}}}}\,{{{{{{{\mathrm{exon}}}}}}}}\,{{{{{{{\mathrm{fragments}}}}}}}}}}{{{{{{{{{\mathrm{Mapped}}}}}}}}\,{{{{{{{\mathrm{reads}}}}}}}}\, \left( {{{{{{{{\mathrm{millions}}}}}}}}} \right) \times {{{{{\mathrm{exon}}}}}}\,{{{{{\mathrm{length}}}}}}\,({{{{{\mathrm{KB}}}}}})}}$$

### Single cell sorting, library preparation, and sequencing for mouse CRC tumors

Cyp51-KO or sg-Ctrl CT26 tumors were harvested at day 26 post orthotopic injection in BALB/c mice. Single cell suspensions of tumor tissues were prepared as described above. Cells from 9 (Cyp51-KO) and 12 (sg-Ctrl) tumors were stained with antibodies for surface proteins at 4 °C for 30 min and sorted into bulk CD45^+^ cells, CD45^+^ CD3^+^ T cells and CD45^-^ BFP^+^ tumor cells with MA900 Multi-Application Cell Sorter (SONY). Sorted cell subsets were combined at a ratio of 50% bulk CD45^+^ cells, 40% CD45^+^ CD3^+^ T cells and 10% tumor cells.

For the quality check and counting of single cell suspension, the cell survival rate is generally above 90%. The cells that have passed the test are washed and resuspended to prepare a suitable cell concentration of 700–1200 cells/μl. Then the cells were loaded ~18,000 cells/chip position using the 10× Chromium Next GEM Single Cell 5’ Kit v2. The system is operated on the machine. GEMs (Gel Bead in Emulsion) were constructed for single cell separation according to the number of cells to be harvested.

After GEMs were normally formed, GEMs were collected for reverse transcription in a PCR machine for labeling. All the subsequent steps were performed following the standard manufacturer’s protocols. The GEMs were oil-treated, and the amplified cDNA was purified by magnetic beads, and then subjected to cDNA amplification and quality inspection. The 5’ Gene Expression Library was constructed with the qualified cDNA. Purified libraries were sequenced on the Illumina nova-seq 6000 instrument using 150-bp paired-end reads. The library construction and sequencing were performed at Genergy Bio-Technology (Shanghai) Co., Ltd.

### Processing of scRNA-seq data and quality control

Cell Ranger (version 7.0) was applied to filter low quality reads, align reads to mouse reference genome (GRCm38), assign cell barcodes, and generate the UMI matrices. The output gene expression matrices were analyzed by R software (v4.2.0) with the Seurat package (version 4.2.0). All samples were merged into one Seurat object using the *merge* function in Seurat. Low quality cells with <200 or >7000 genes detected, <500 or >100,000 UMI counts detected or >5% mitochondrial UMI counts detected were removed.

### Dimension reduction, unsupervised clustering and cell-type annotation

Dimension reduction and unsupervised clustering were performed according to the standard workflow in Seurat. *SCTransform* function was applied to normalize and find highly variable genes (HVGs) within the single-cell gene expression data. Mitochondrial genes, dissociation-induced genes and HLA genes were removed from HVGs for downstream analyses. Then, the effect of the percentage of mitochondrial gene counts was regressed out by using *SCTransform* function with parameter “*vars.to.regress* = ‘*percent.mt*’”. A principal component analysis (PCA) matrix was calculated to reduce noise by using *RunPCA* with default parameters. After PCA analysis, we use *Findneighbors* and *FindClusters* (by Louvain algorithm) function provided by Seurat. Then UMAP and graph-based clustering were performed on the object for visualization and cell clustering by *RunUMAP* function. The main immune cell types were annotated based on the expression pattern of DEGs and the well-known cellular markers from the literature. In the first-round of unsupervised clustering of all cells, we found 5 spread small clusters (less than 10 cells) of WT group which might be caused by sequencing noise. Therefore, we removed these clusters for downstream analysis.

To identify subtypes within the myeloid cell cluster, we performed a second-round of unsupervised clustering on neutrophils and Macro/Mono like cell. The second-round clustering procedure was alike the first-round clustering, which started from the expression matrix of the neutrophils and Macro/Mono like cell subsets and then identified HVGs, calculated the PCA matrix, detected cell clusters by Louvain algorithm and performed dimensionality reduction for visualization. The number of principal components was determined by the *Elbowplot* function in Seurat. DEGs were detected using the *FindAllMarkers* function with default parameters. Finally, we got 20 clusters for further analysis under parameter resolution = 0.3 condition.

### Gene set scoring and graph plotting of scRNA-seq data

The gene set scoring was completed by *AddModuleScore* function of Seurat. All the used gene sets will be shown in Dataset EV2. Violin-Boxplot graphs, bar graphs and regression dot plot graphs were plotted by R package ggplot2. Gene expression Z-score heatmap was completed with function *pheatmap* which provided by R package pheatmap. The dotplot of gene expression and top10 DEGs heatmap over all clusters were plotted by function *Dotplot* and *Doheatmap* of package Seurat, respectively.

### Cell–cell interaction analysis

Cell-Cell interaction analysis was performed using CellChat software (version 1.5.0) (Jin et al, 2021) and only “Secreted Signaling” subsets of CellChat database were used. Width of the line represented the number of possibly activated protein couples among the linked cell types.

### Statistical analysis

Statistical analyses were performed using GraphPad Prism 9 (GraphPad Software). Statistical significance was determined as indicated in the figures. Significance was set to *P* < 0.05 and represented as *P < 0.05, **P < 0.01, ***P < 0.001, ****P < 0.0001.

For unpaired samples, the data distribution was first checked using a D’Agostino & Pearson normality test. If the data fitted a normal distribution, a two-tailed unpaired *t* test was used when variances were similar, whereas a two-tailed unpaired *t* test with Welch’s correction was used when variances were different. If the data did not fit a normal distribution, a Mann–Whitney test was used.

For paired samples, the data distribution was first checked using a D’Agostino and Pearson normality test. If the data fitted a normal distribution, a two-tailed paired *t* test was used to compare two groups. If the data did not fit a normal distribution, a two-tailed Wilcoxon matched-pairs signed-rank test was used to compare two groups. Measurements were taken from distinct samples. Data are mean ± s.e.m.

## For more information

Chenqi Xu Lab website: http://xulab.sibcb.ac.cn.

Zheng-Jiang Zhu Lab website: https://www.zhulab.cn.

## Supplementary information

Appendix

Dataset EV1

Dataset EV2

Dataset EV3

Source Data Fig. 1

Source Data Fig. 2

Source Data Fig. 3

Source Data Fig. 4

Source Data Fig. 5

Source Data Fig. 6

Source Data for Expanded View and Appendix

Expanded View Figures

## Data Availability

The datasets produced in this study are available in the following databases: RNA-Seq: Gene Expression Omnibus (GEO) database (accession: GSE249124) [URL: https://www.ncbi.nlm.nih.gov/geo/query/acc.cgi?acc=GSE249124]. RNA-Seq: Gene Expression Omnibus (GEO) database (accession: GSE248570) [URL: https://www.ncbi.nlm.nih.gov/geo/query/acc.cgi?acc=GSE248570]. All codes for data cleaning and analysis are available upon request.
