# Supplementary material for: Shaping immune landscape of colorectal cancer by cholesterol metabolites
Source: EMBO Mol Med. 2024 Jan 2;16(2):7. doi: 10.1038/s44321-023-00015-9 (PMC10897227; doi:10.1038/s44321-023-00015-9)
Supplement: Supplementary file 1 — Appendix [file 44321_2023_15_MOESM1_ESM.docx]

**Shaping immune landscape of colorectal cancer by cholesterol metabolites**

**Appendix**

**Contents:**

Appendix Figure S1. Validation of sterol identifications in human CRC tissue samples with chemical standards.

Appendix Figure S2. Cholesterol biosynthesis of different cell populations in the human CRC microenvironment.

Appendix Figure S3. Stratification of MSS CRC with clinical stages.

Appendix Figure S4. Analyses of MSS CRC data with other stratification rules.

Appendix Figure S5. Transcription factor (TF) binding sites or motifs differentially enriched in gene promoters of the major enzymes of cholesterol biosynthesis.

Appendix Figure S6. Ketoconazole treatment reduced secretion of distal cholesterol precursors of MSS-CRC CT26 and Caco2 cells.

Appendix Figure S7. Gene signatures of the defined cell clusters in Figure 6E.


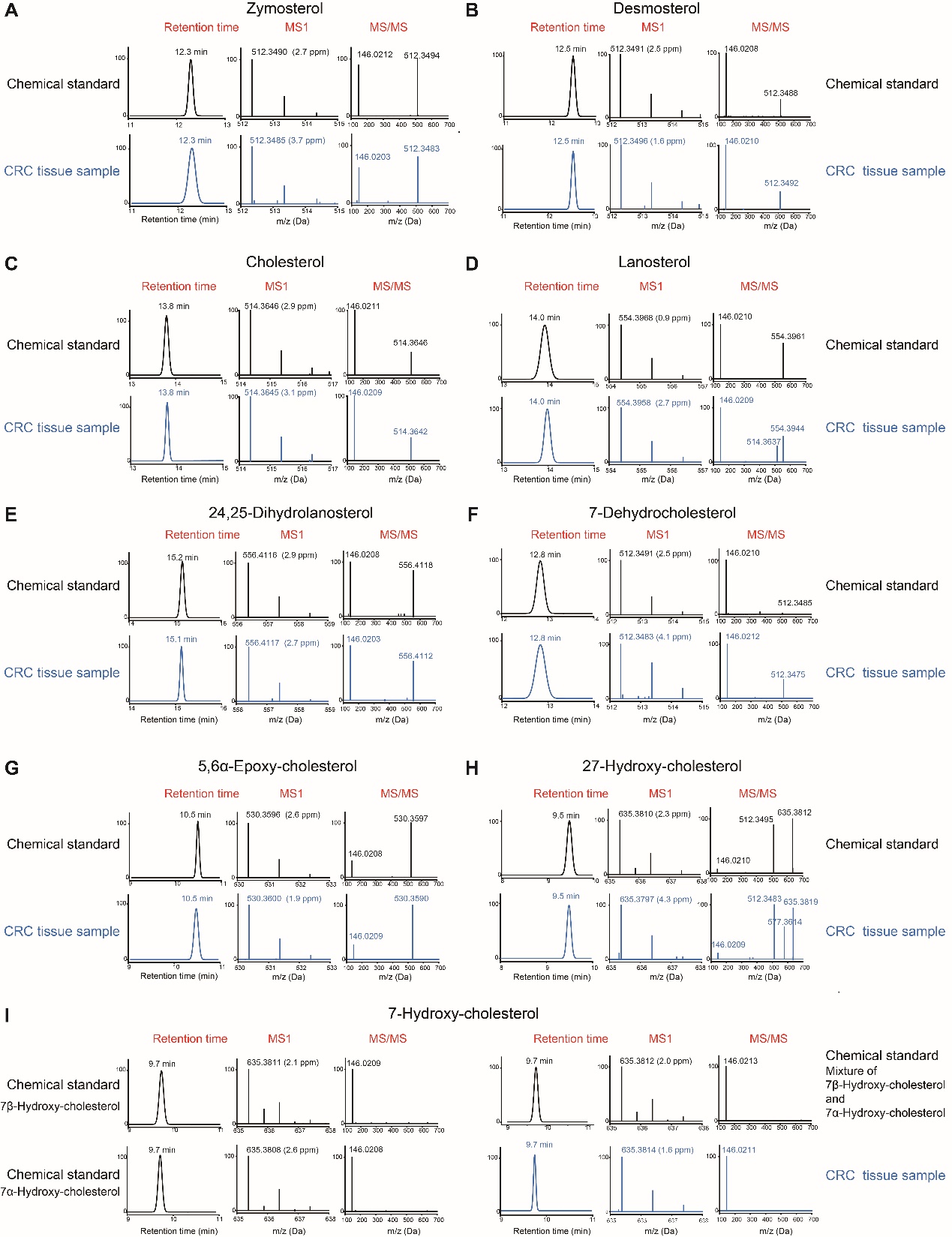


**Appendix Figure S1. Validation of sterol identifications in human CRC tissue samples with chemical standards.**

The validation of sterol identifications in the sterol biosynthesis pathway as described in Figure 2 using purchased chemical standards.


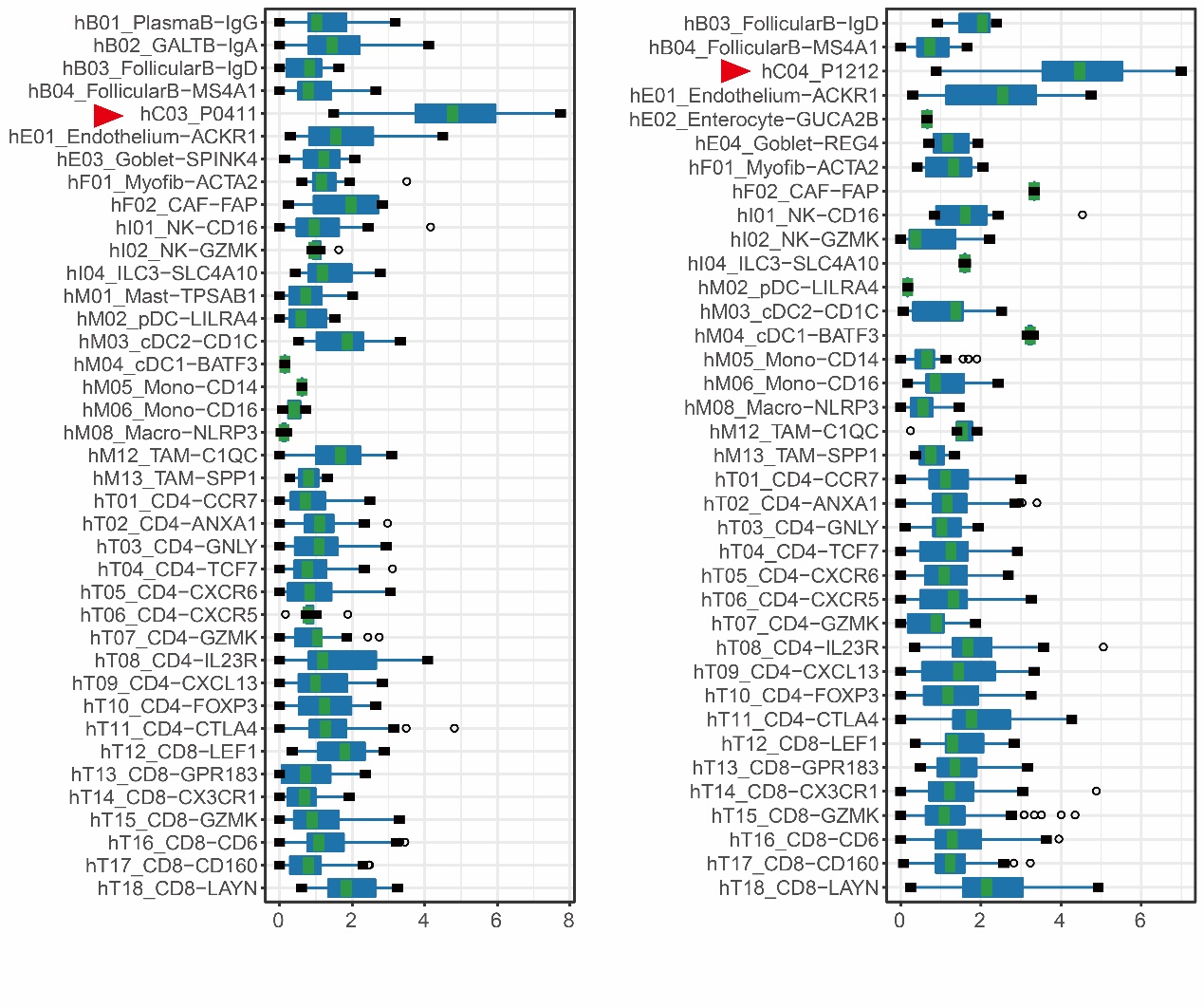


**Appendix Figure S2. Cholesterol biosynthesis of different cell populations in the human CRC microenvironment.**

The cholesterol biosynthesis scores of different cell populations in MSS CRC identified by the single-cell RNA sequencing (Zhang et al., 2020). Data from two MSS CRC patients were displayed. The tumor cell populations were labelled with red triangle. Box indicates the interquartile range (25% - 75%); center line indicates the median. Whiskers denote 1.5× interquartile range. N number of each cell population was listed in Dataset EV3.


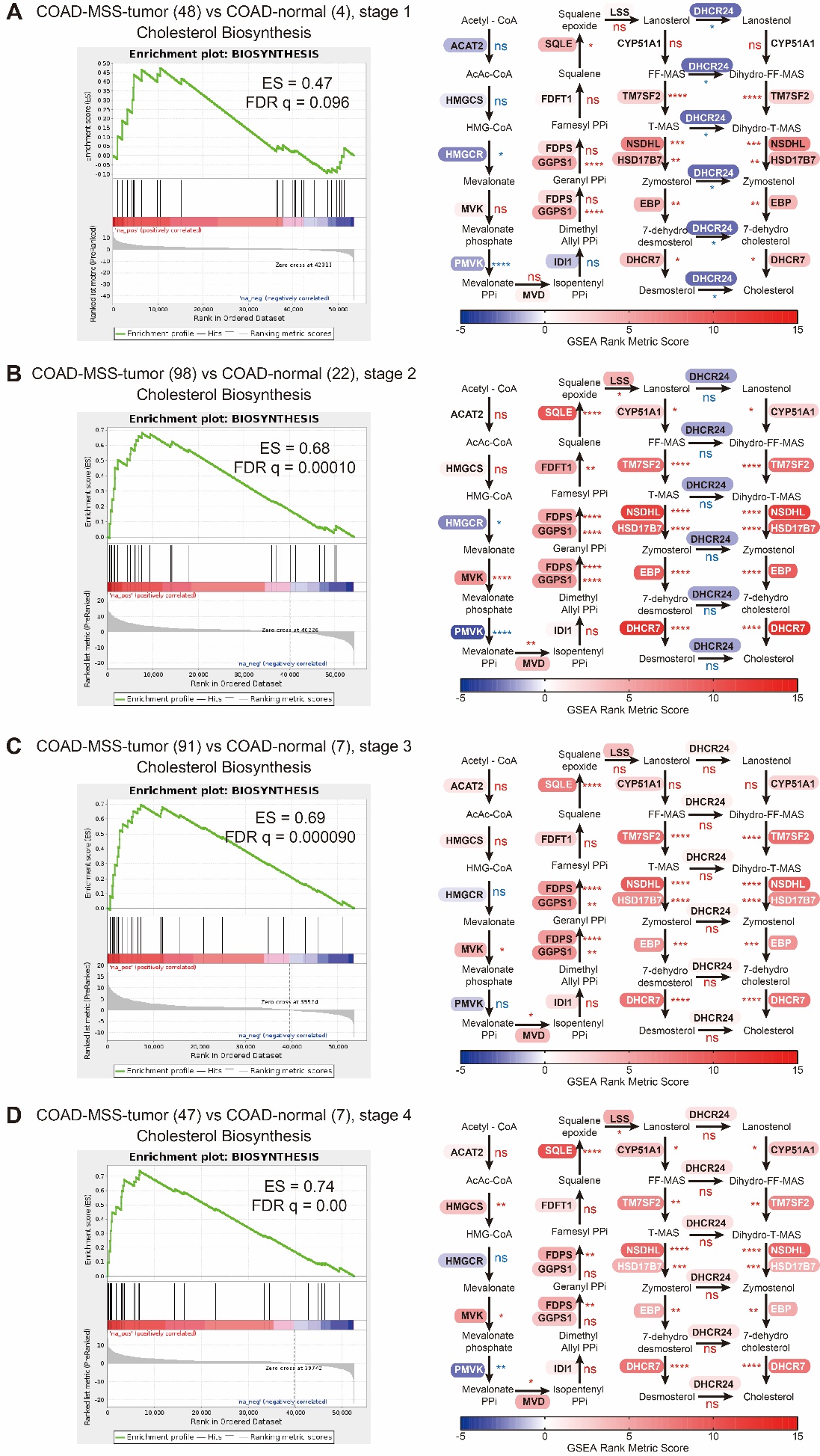


**Appendix Figure S3. Stratification of MSS CRC with clinical stages.**

Left panel: gene set enrichment analysis (GSEA) of the cholesterol biosynthesis pathway in MSS tumors and normal tissues from Stage 1-4 patients deposited in the TCGA colon cancer (COAD) database. Right panel: GSEA rank metric score and transcriptional levels of enzymes in the cholesterol biosynthesis pathway. Blue and red represents downregulation and upregulation in tumor. P value of gene expression between tumor and normal tissue (two tailed Mann-Whitney test) is labelled by the side of each gene. (ns P > 0.05, *P ≤ 0.05, **P < 0.01, ***P < 0.001, ****P < 0.0001)

**
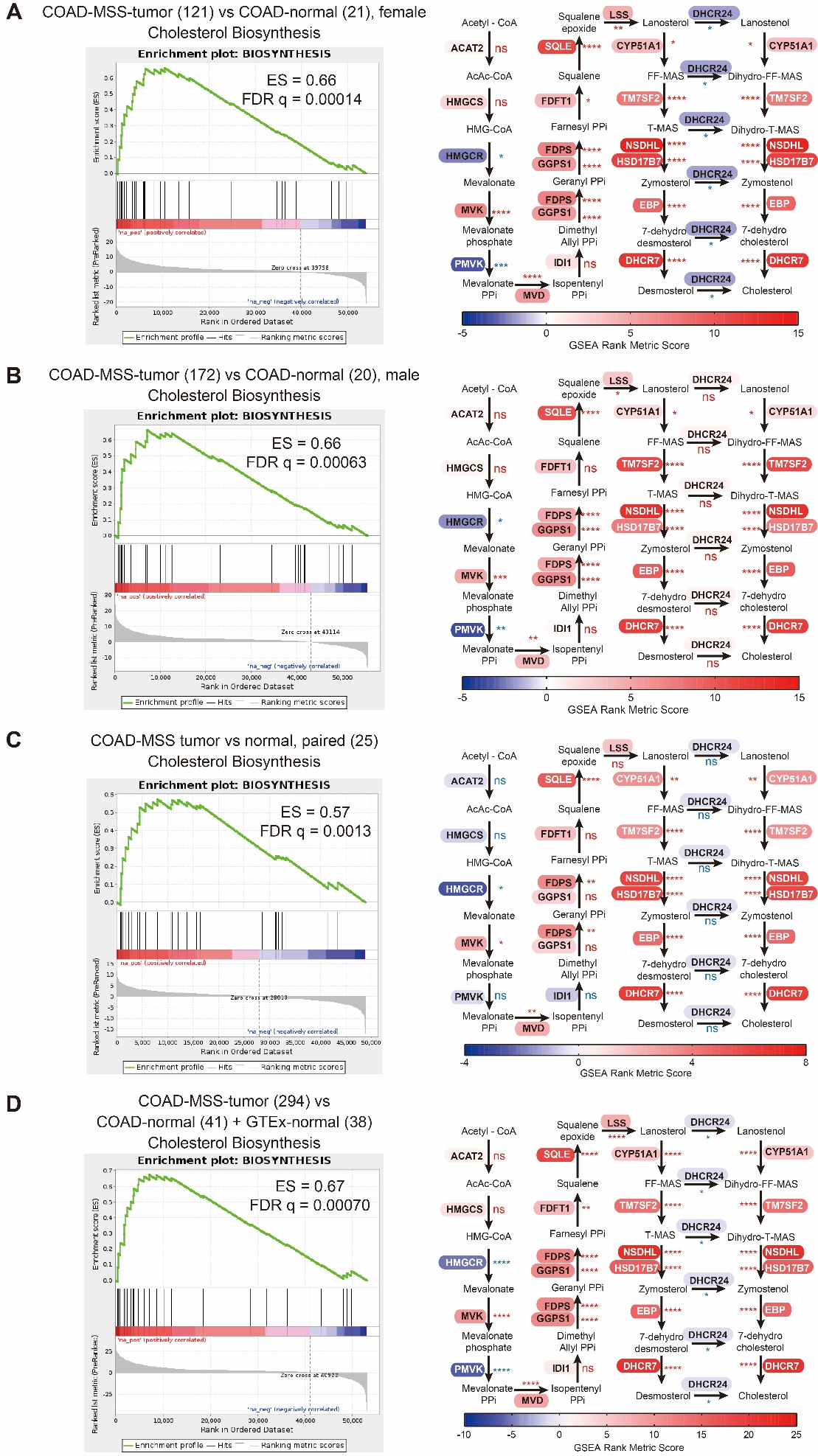
**

**Appendix Figure S4. Analyses of MSS CRC data with other stratification rules.**

**A, B,** Comparison of MSS CRC tumor tissue with normal tissue from patients with the same sex in COAD (**A**, female; **B**, male).

**C**, Paired comparison of MSS CRC tumor tissue with normal tissue isolated from the same patient in COAD.

**D**, Comparison of MSS CRC tumor tissue with normal tissue from COAD and GTEx. MSS tumors (n = 294 in COAD), normal tissues from two database (n = 41 in COAD (CRC patients) plus n = 38 in GTEx (non-CRC-patient people)).

Left panel: GSEA of cholesterol biosynthesis pathway. Right panel: GSEA rank metric score and transcriptional levels of enzymes in the cholesterol biosynthesis pathway. Blue and red represents downregulation and upregulation in tumor. P value of gene expression between tumor and normal tissue (two tailed Mann-Whitney test) is labelled by the side of each gene. Ns P > 0.05, *P ≤ 0.05, **P < 0.01, ***P < 0.001, ****P < 0.0001.


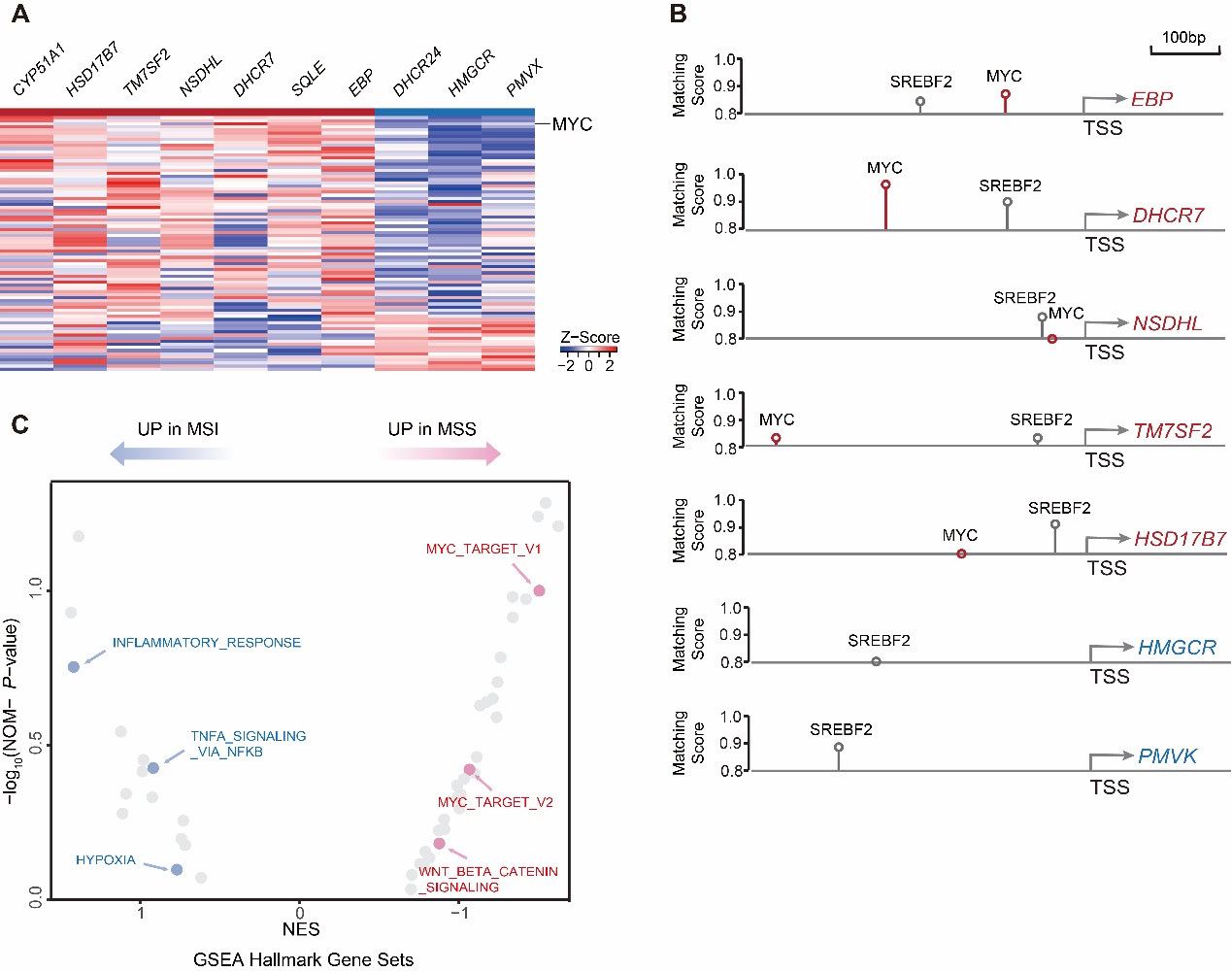


**Appendix Figure S5. Transcription factor (TF) binding sites or motifs differentially enriched in gene promoters of the major enzymes of cholesterol biosynthesis.**

**A**, Heatmap showing z-scores of TF binding motifs preferentially enriched at the promoters of upregulated and downregulated cholesterol biosynthesis enzymes. The Motifs enriched (z-score > 0) in 5 out of 7 upregulated or 2 out of 3 downregulated gene promoters are shown.

**B**, Lollipops plots indicating the location and matching score of MYC motif and SREBF2 motif in the gene promoters of the major enzymes in the pathway.

**C**, GSEA analysis of significantly up- or down-regulated gene signatures in MSS CRC relative to MSI CRC is shown. Representative enriched gene signatures are indicated by arrowheads. NOM-P-value, nominal P-value by an empirical phenotype-based permutation test; NES, normalized enrichment score.


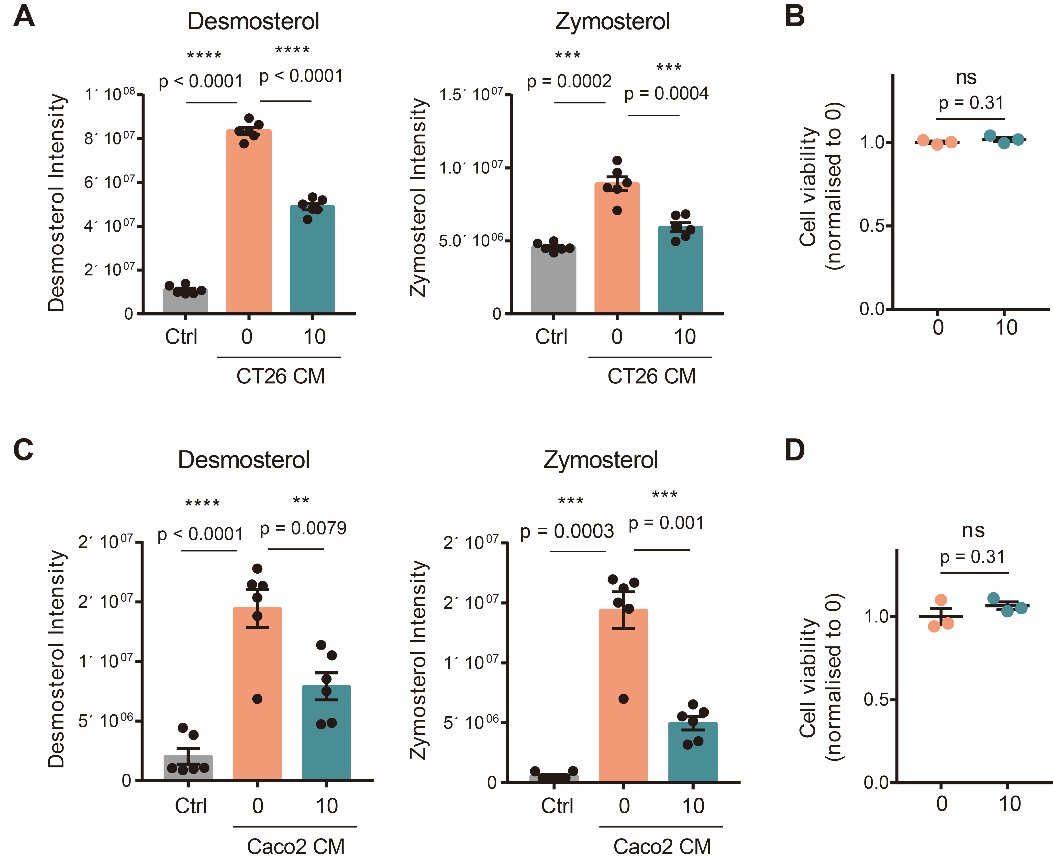


**Appendix Figure S6. Ketoconazole treatment reduced secretion of distal cholesterol precursors of MSS-CRC CT26 and Caco2 cells.**

**A,** Intensity of indicated distal cholesterol precursors in control blank medium (Ctrl), and conditioned media (CM) of CT26 cells treated with ketoconazole at indicated concentrations (μM), n = 6.

**B,** Cell viability of CT26 cells treated with ketoconazole at indicated concentrations (μM), n = 3.

**C,** Intensity of indicated distal cholesterol precursors in control blank medium (Ctrl), and conditioned media (CM) of Caco2 cells treated with ketoconazole at indicated concentration (μM), n = 6.

**D,** Cell viability of Caco2 cells treated with ketoconazole at indicated concentration (μM), n = 3.

Data information: in **(A-D),** data are presented as mean ± SEM. Two-tailed unpaired *t* test was used when variances were similar, whereas a two-tailed unpaired *t* test with Welch’s correction was used when variances were different. Data are representative of two independent experiments (**B, D**).


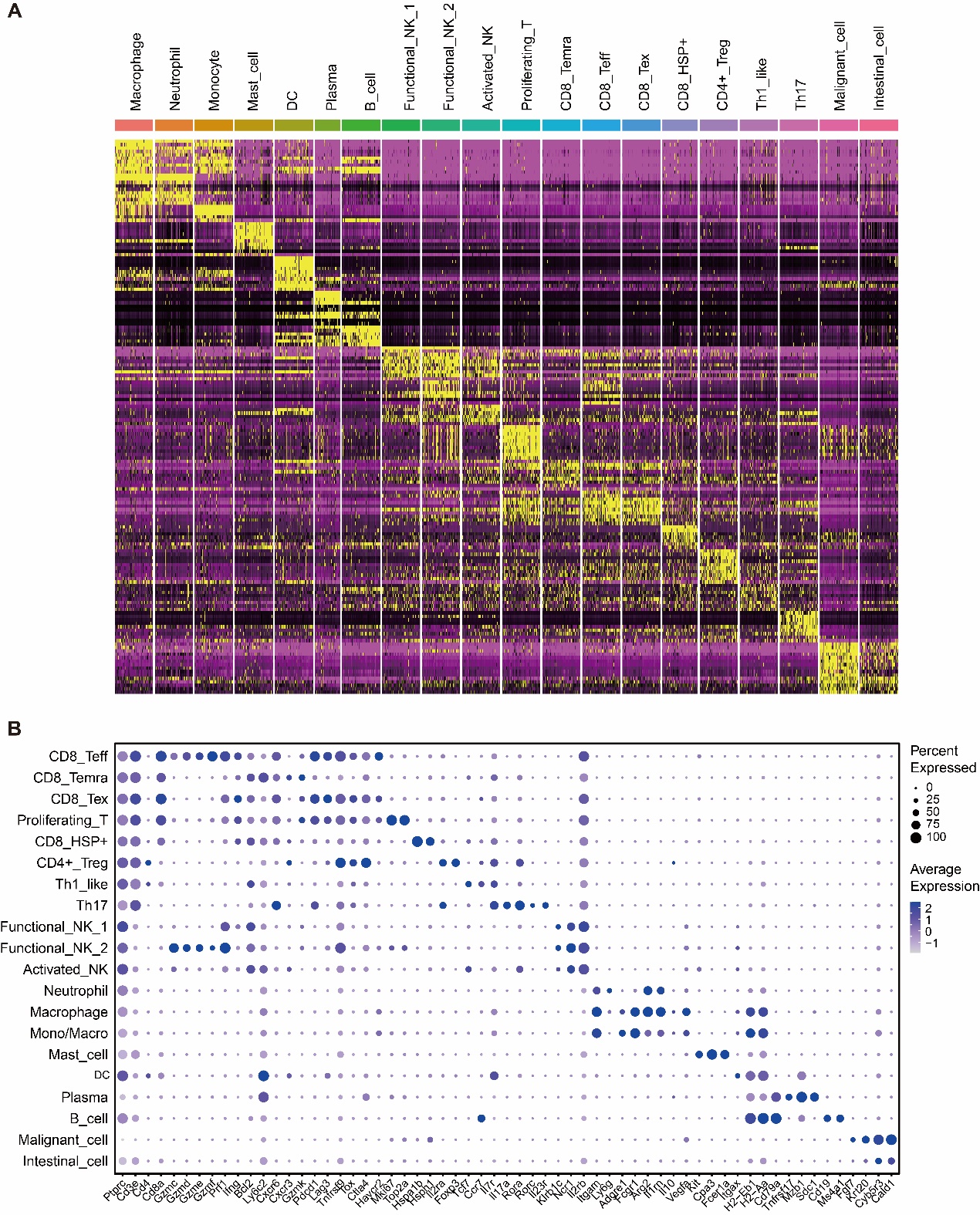


**Appendix Figure S7. Gene signatures of the defined cell clusters in Figure 6E.**

**A,** Gene expression heatmap of the defined clusters (top10 DEGs in Dataset EV1). If the cluster had more than 300 cells, randomly chosen 300 cells were displayed. The color reflects the row-wise-scaled CPM value.

**B**, Dotplot showing marker genes across all clusters. Dot size indicates percentage of expressing cells, and dot color indicates expression level.

**References:**

Zhang, L., Li, Z., Skrzypczynska, K.M., Fang, Q., Zhang, W., O'Brien, S.A., He, Y., Wang, L., Zhang, Q., Kim, A.*, et al.* (2020). Single-Cell Analyses Inform Mechanisms of Myeloid-Targeted Therapies in Colon Cancer. Cell *181*, 442-459 e429.
