# Supplementary material for: Shaping immune landscape of colorectal cancer by cholesterol metabolites
Source: EMBO Mol Med. 2024 Jan 2;16(2):7. doi: 10.1038/s44321-023-00015-9 (PMC10897227; doi:10.1038/s44321-023-00015-9)
Supplement: Supplementary file 8 — Source Data Fig. 4 [file 44321_2023_15_MOESM8_ESM.zip › 4C, 4G/Fig 4C, 4G_WesternBlot_cropping area.pptx]

## Slide 1
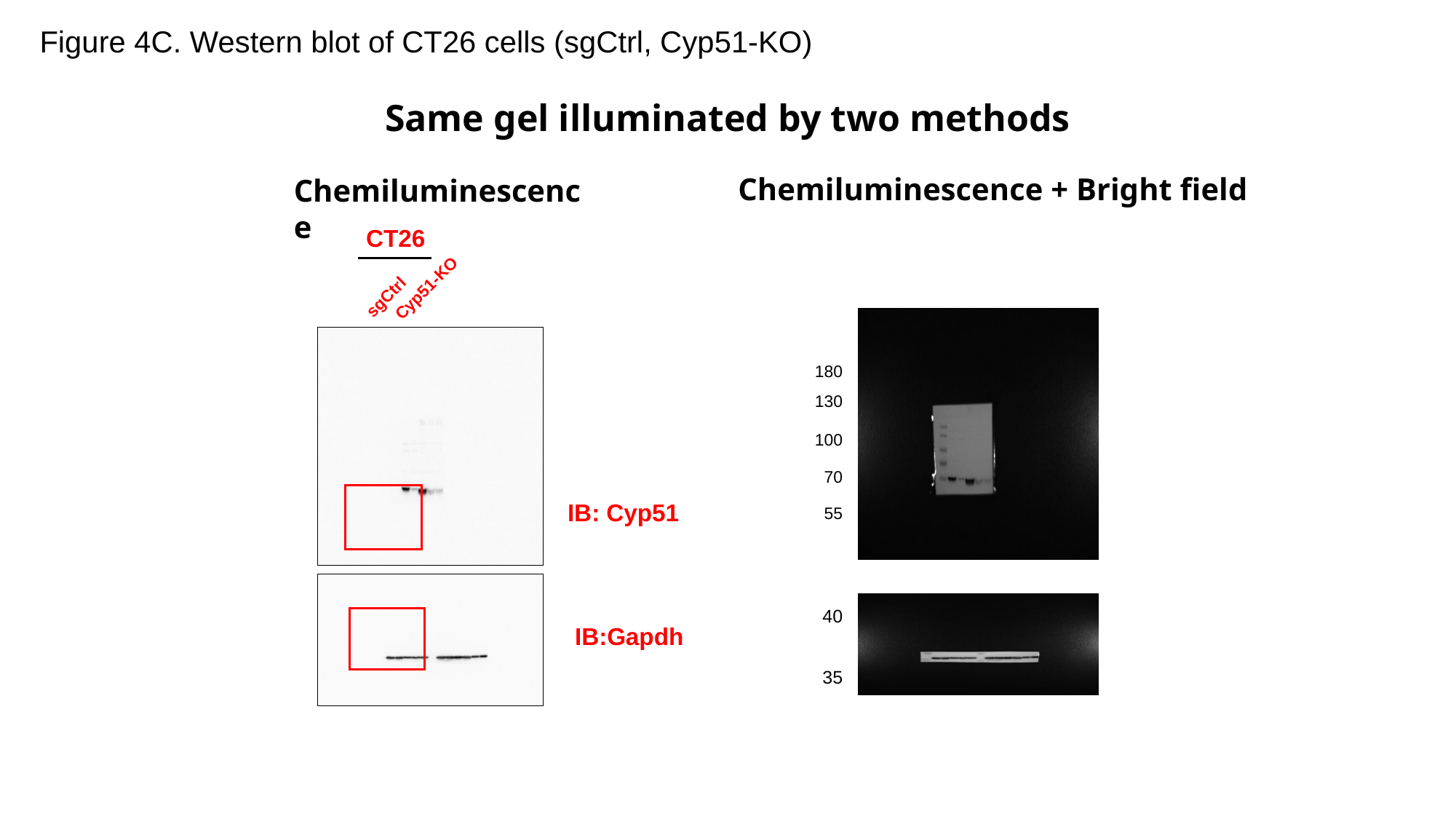

Figure 4C. Western blot of CT26 cells (sgCtrl, Cyp51-KO)
Same gel illuminated by two methods
Chemiluminescence + Bright field
Chemiluminescence
CT26
Cyp51-KO
sgCtrl
180
130
100
70
IB: Cyp51
55
40
IB:Gapdh
35

## Slide 2
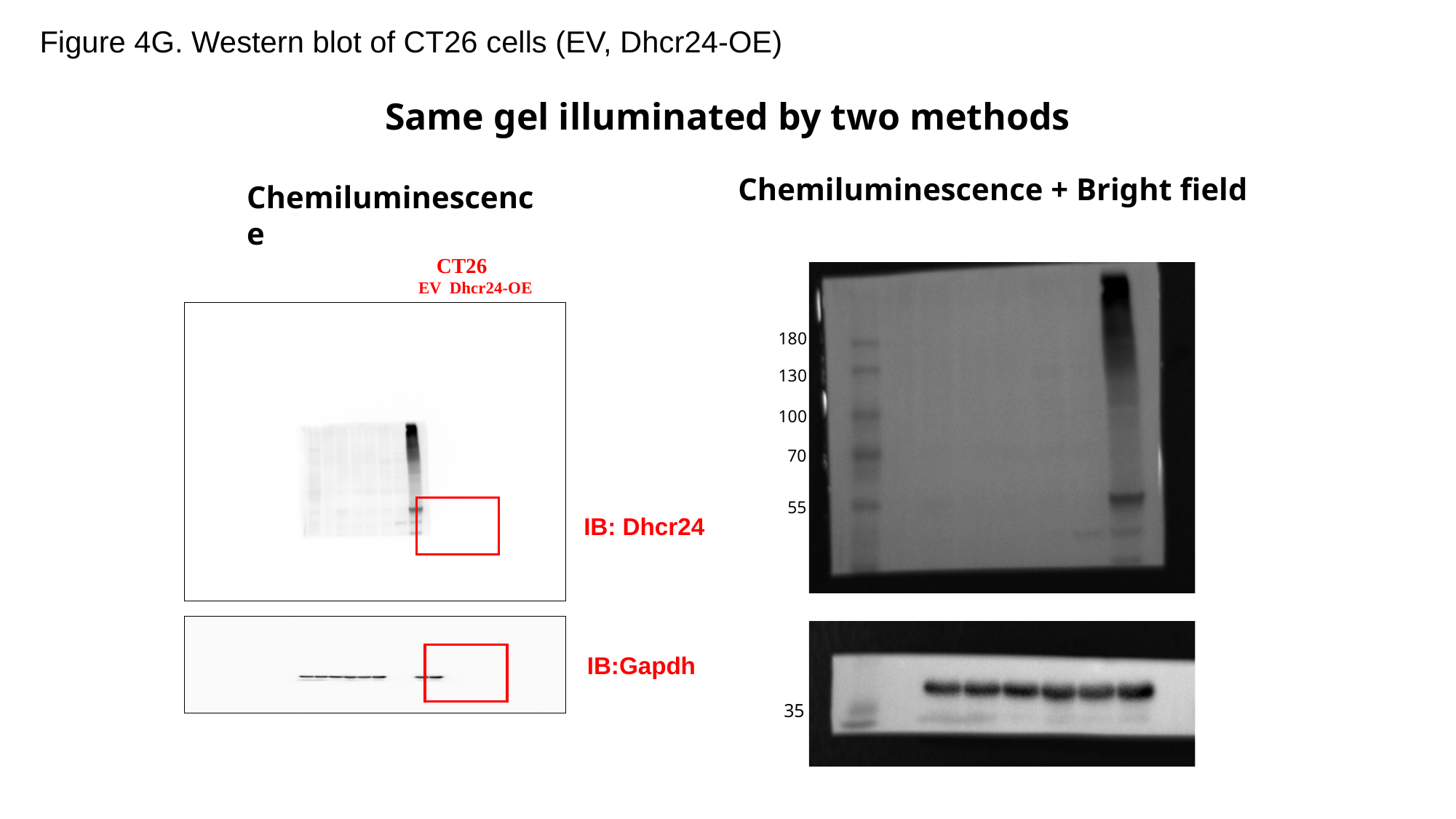

Figure 4G. Western blot of CT26 cells (EV, Dhcr24-OE)
Same gel illuminated by two methods
Chemiluminescence + Bright field
Chemiluminescence
 CT26
 EV Dhcr24-OE
180
130
100
70
55
IB: Dhcr24
IB:Gapdh
35
