# Supplementary figures and images for: Shaping immune landscape of colorectal cancer by cholesterol metabolites
Source: EMBO Mol Med. 2024 Jan 2;16(2):7. doi: 10.1038/s44321-023-00015-9 (PMC10897227; doi:10.1038/s44321-023-00015-9)

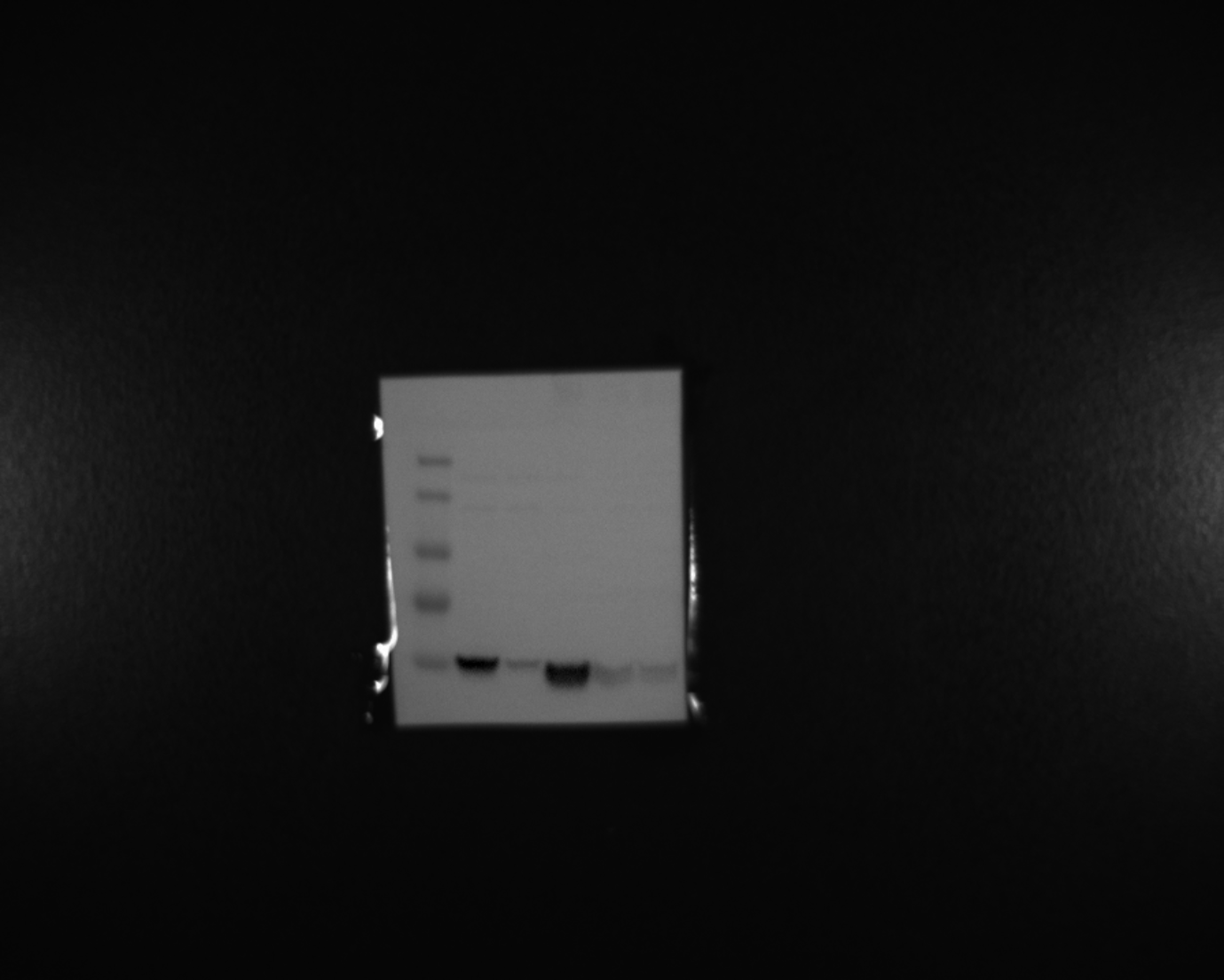

Supplement: Supplementary file 8 — Source Data Fig. 4 [file 44321_2023_15_MOESM8_ESM.zip › 4C, 4G/Fig 4C_Raw Data_ CT26_Cyp51-KO/Cyp51/Chemiluminescence + Bright field.tif]

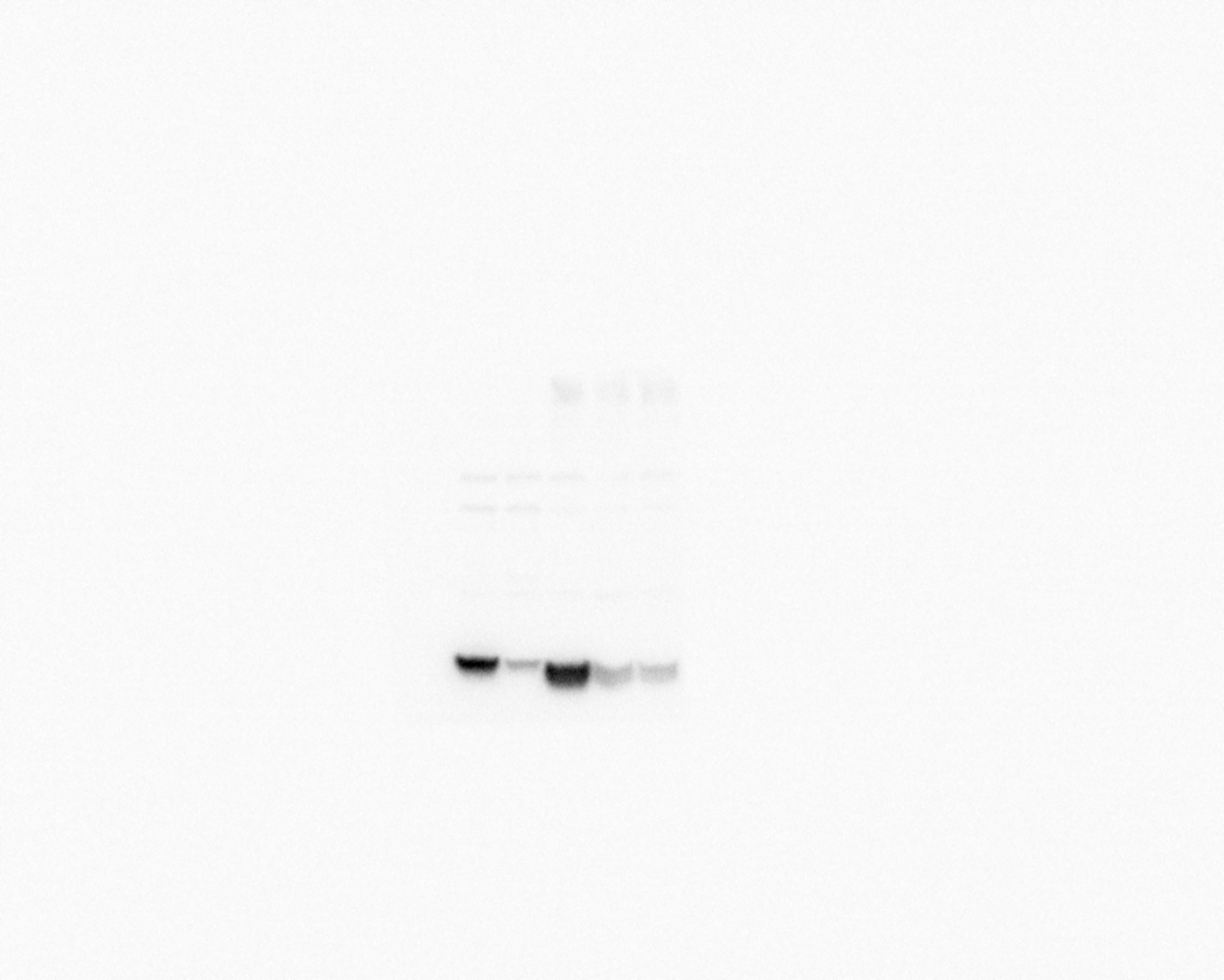

Supplement: Supplementary file 8 — Source Data Fig. 4 [file 44321_2023_15_MOESM8_ESM.zip › 4C, 4G/Fig 4C_Raw Data_ CT26_Cyp51-KO/Cyp51/Chemiluminescence.tif]

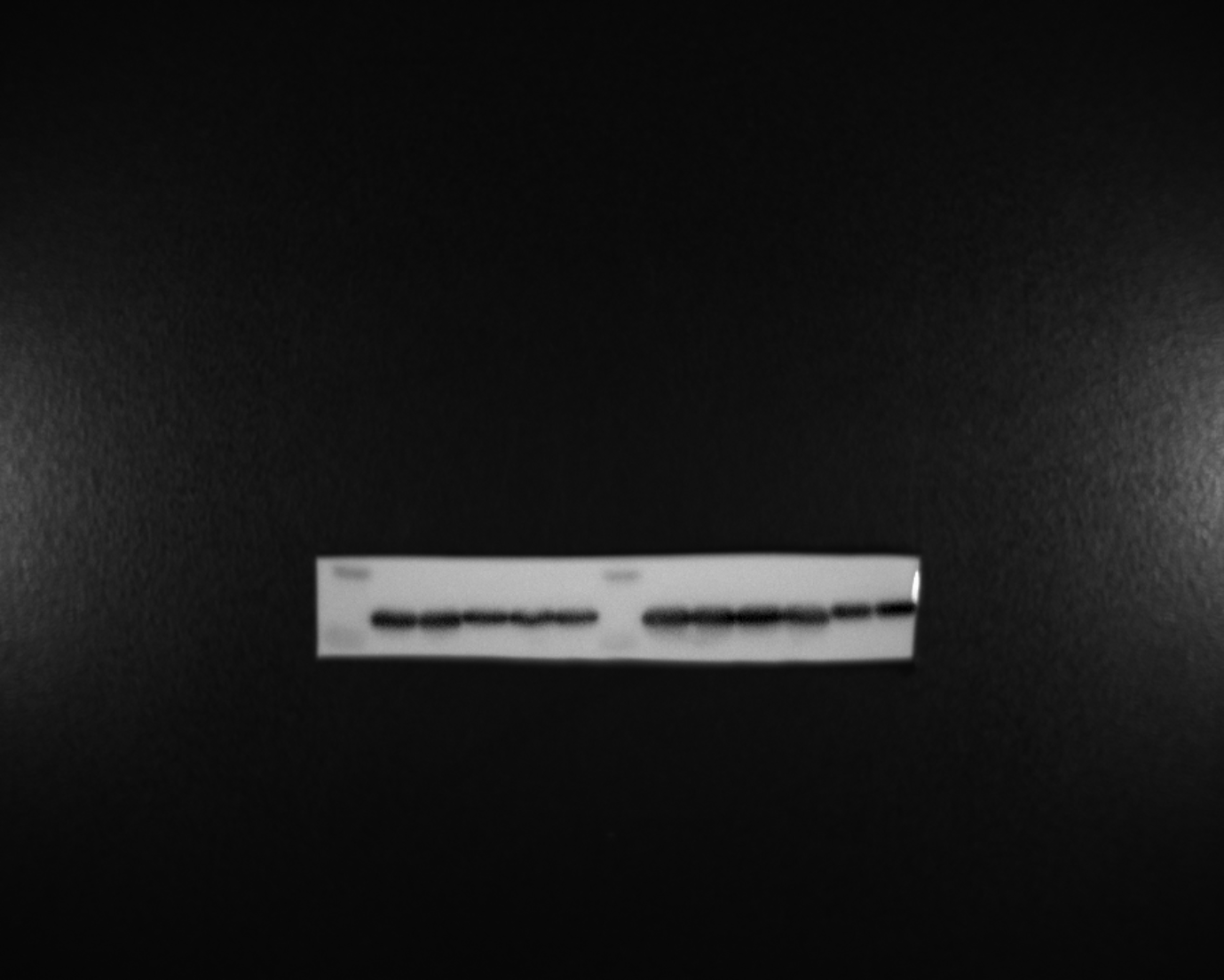

Supplement: Supplementary file 8 — Source Data Fig. 4 [file 44321_2023_15_MOESM8_ESM.zip › 4C, 4G/Fig 4C_Raw Data_ CT26_Cyp51-KO/Gapdh/Chemiluminescence + Bright field.tif]

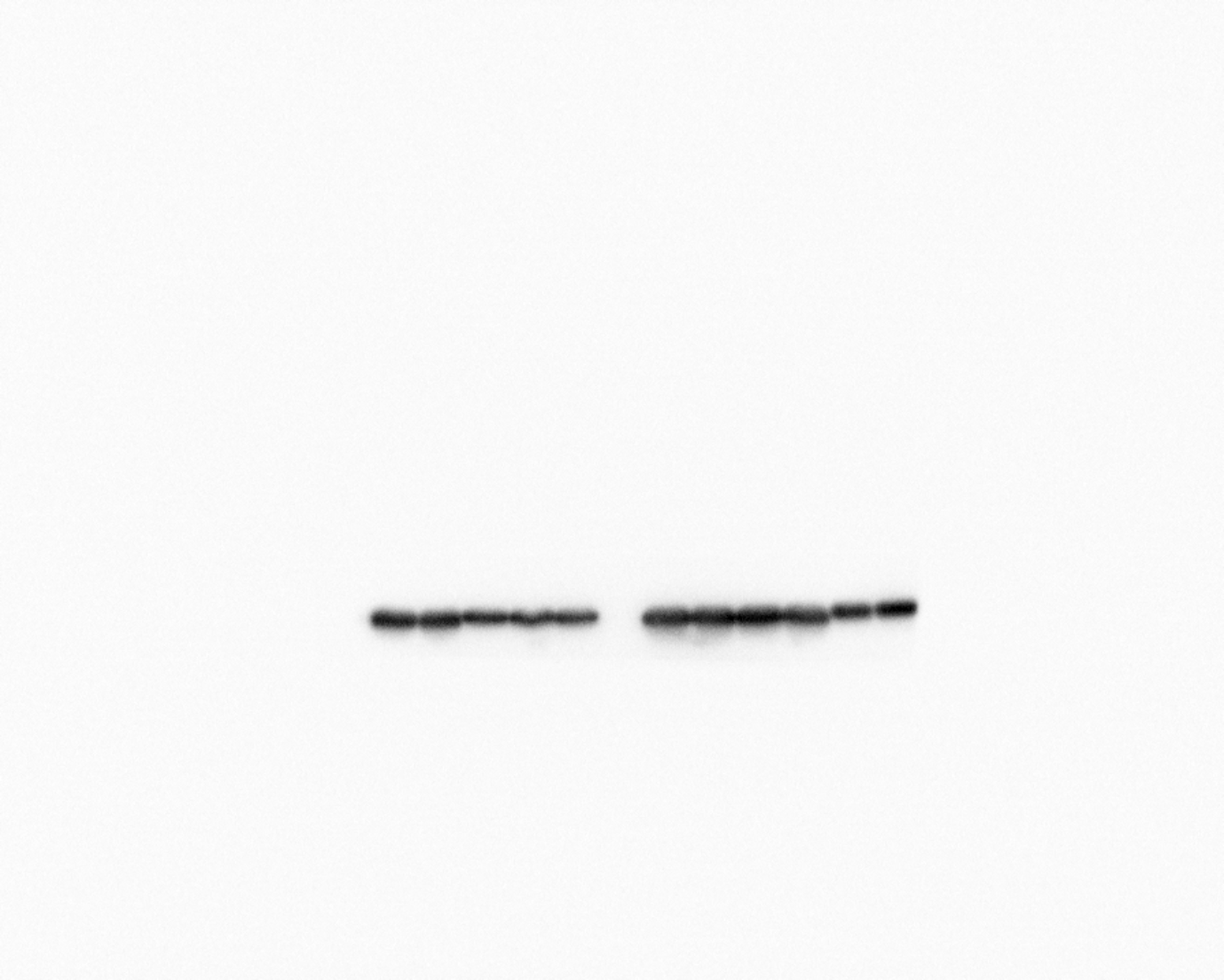

Supplement: Supplementary file 8 — Source Data Fig. 4 [file 44321_2023_15_MOESM8_ESM.zip › 4C, 4G/Fig 4C_Raw Data_ CT26_Cyp51-KO/Gapdh/Chemiluminescence.tif]

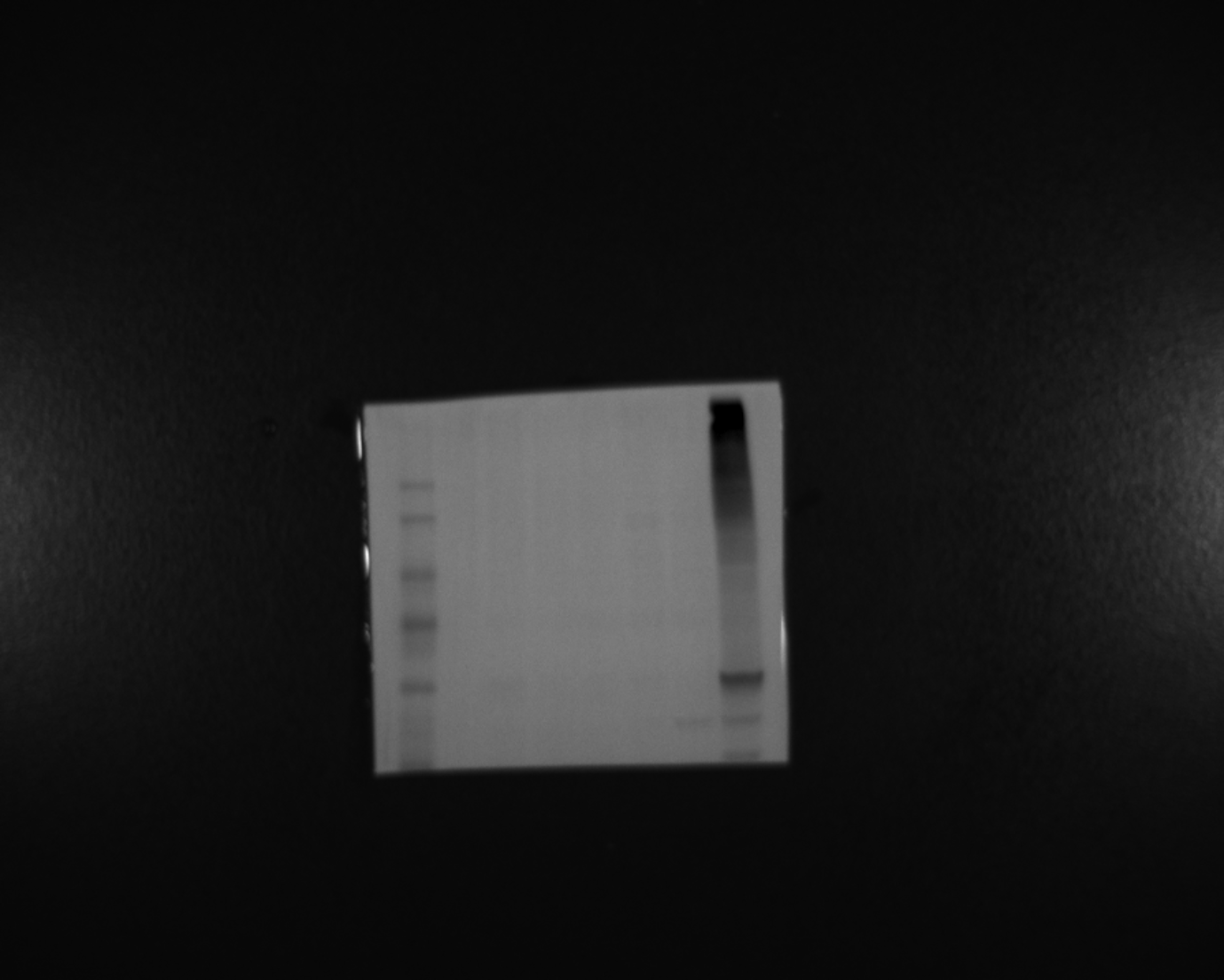

Supplement: Supplementary file 8 — Source Data Fig. 4 [file 44321_2023_15_MOESM8_ESM.zip › 4C, 4G/Fig 4G_Raw Data_CT26_Dhcr24-OE/Dhcr24/Chemiluminescence + Bright field.tif]

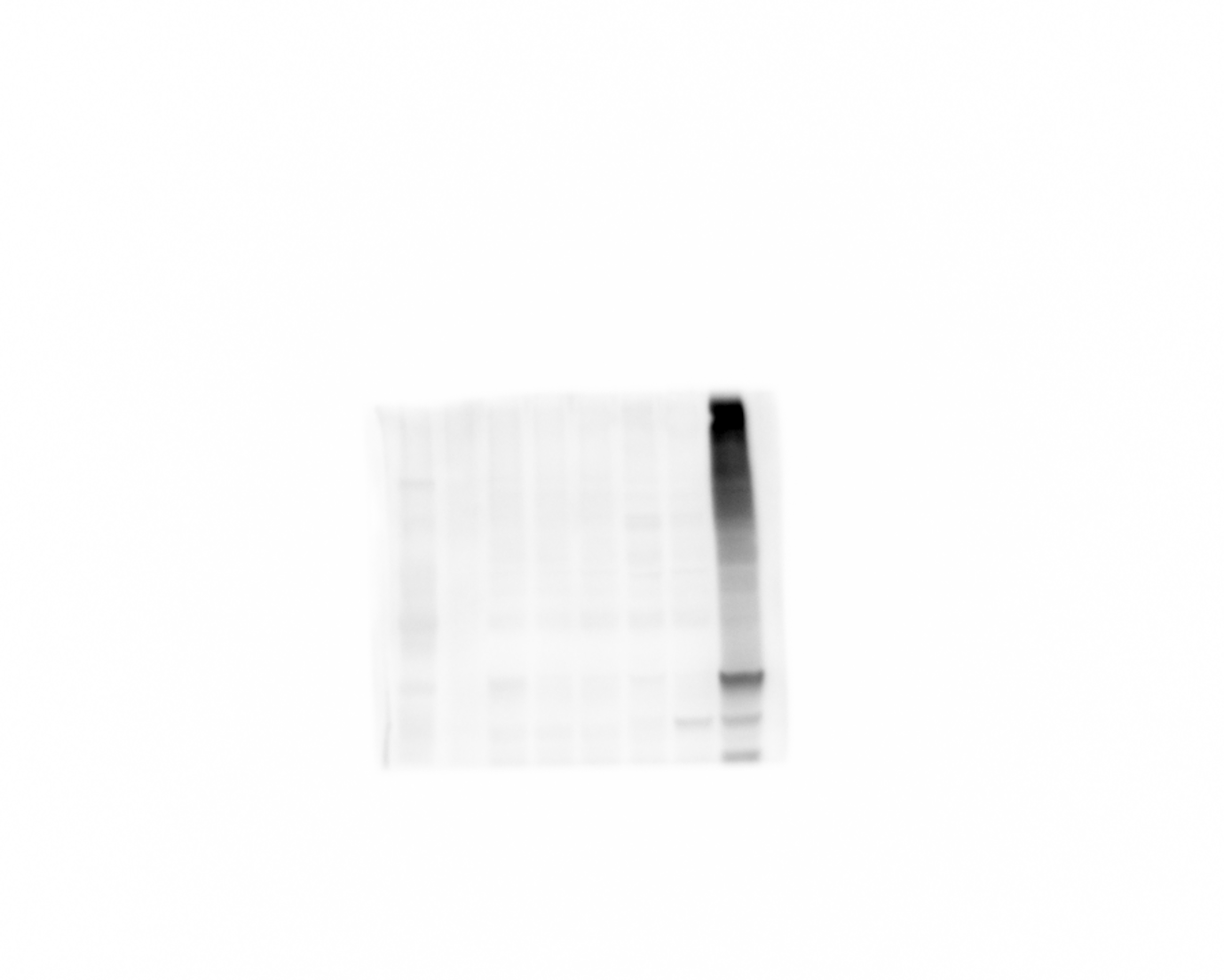

Supplement: Supplementary file 8 — Source Data Fig. 4 [file 44321_2023_15_MOESM8_ESM.zip › 4C, 4G/Fig 4G_Raw Data_CT26_Dhcr24-OE/Dhcr24/Chemiluminescence.tif]

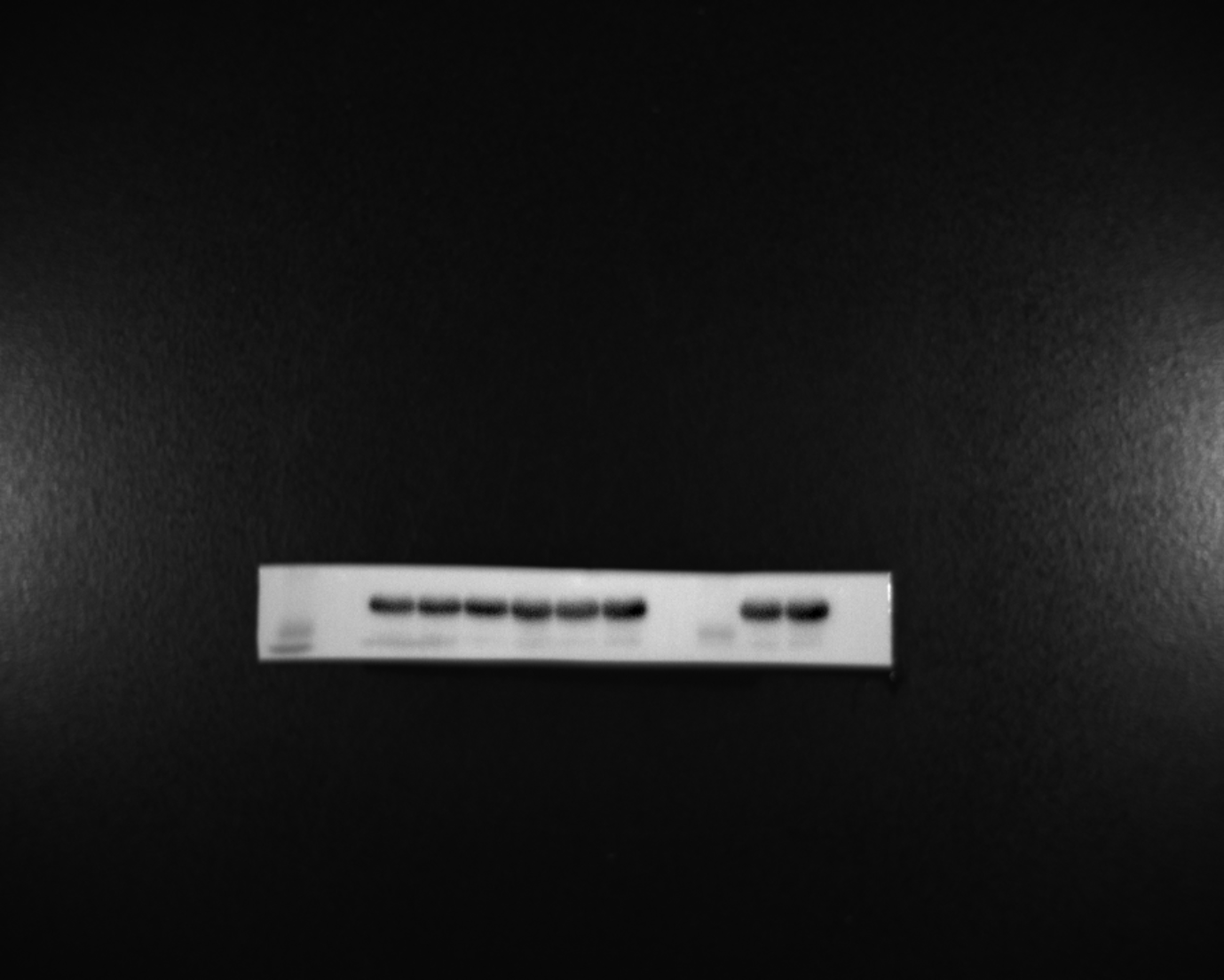

Supplement: Supplementary file 8 — Source Data Fig. 4 [file 44321_2023_15_MOESM8_ESM.zip › 4C, 4G/Fig 4G_Raw Data_CT26_Dhcr24-OE/Gapdh/Chemiluminescence + Bright field.tif]

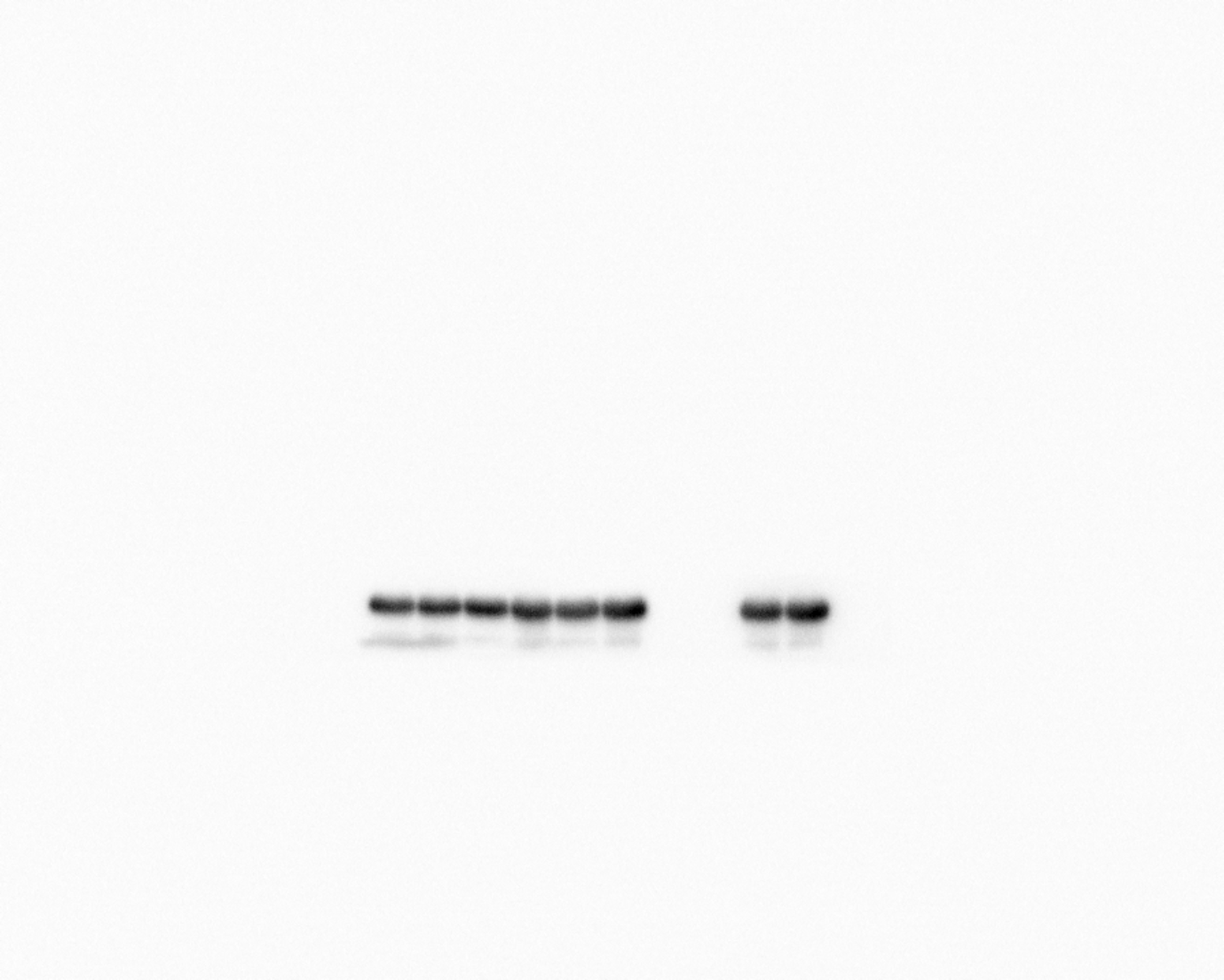

Supplement: Supplementary file 8 — Source Data Fig. 4 [file 44321_2023_15_MOESM8_ESM.zip › 4C, 4G/Fig 4G_Raw Data_CT26_Dhcr24-OE/Gapdh/Chemiluminescencefield.tif]
