# Supplementary material for: Shaping immune landscape of colorectal cancer by cholesterol metabolites
Source: EMBO Mol Med. 2024 Jan 2;16(2):7. doi: 10.1038/s44321-023-00015-9 (PMC10897227; doi:10.1038/s44321-023-00015-9)
Supplement: Supplementary file 12 — Expanded View Figures [file 44321_2023_15_MOESM12_ESM.pdf]

Expanded View Figures

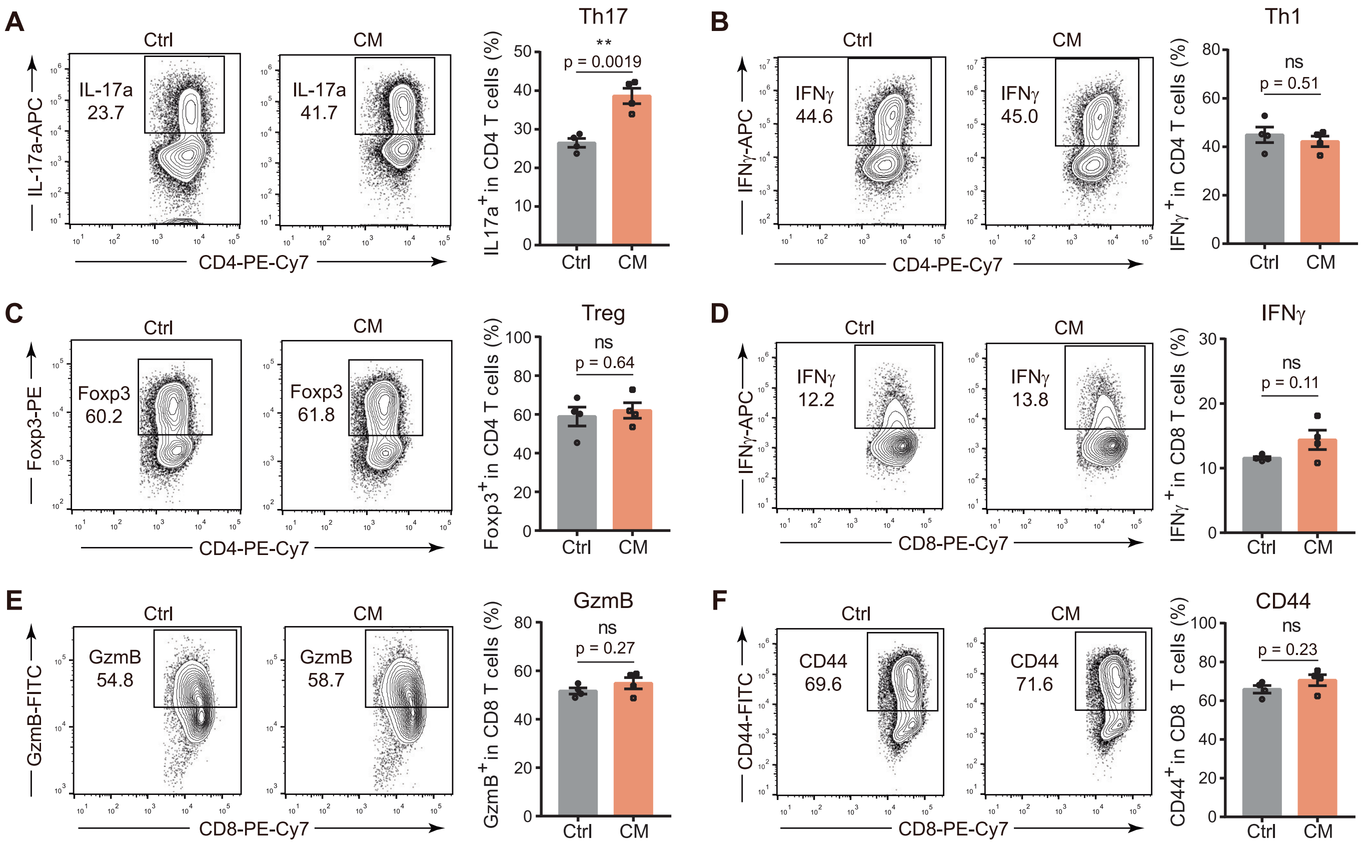

Figure EV1. Caco2 cells polarized Th17 cells without affecting other T cell subtypes.

(**A–C**) Th17 (**A**), Th1 (**B**), and Treg (**C**) induction in the presence of the conditioned medium of Caco2 cells. Naive $CD4^+$ T cells were stimulated with plate-bound anti-CD3/CD28 and cytokines to induce Th17, Th1 or Treg differentiation. Concentrated conditioned medium (CM) or control blank medium (Ctrl) (2.5% v/v) was added in on Day 2. Th17 (IL-17a^+ gated on $CD4^+$), Th1 ($IFN\gamma^+$ gated on $CD4^+$), and Treg ($Foxp3^+$ gated on $CD4^+$) percentages were analyzed on Day 4 ($n = 4$). (**D**, **E**) $CD8^+$ T cell effector functions in the presence of the conditioned medium of Caco2 cells. Naive $CD8^+$ T cells were stimulated with plate-bound anti-CD3/CD28. Concentrated conditioned medium (CM) or control blank medium (Ctrl) (2.5% v/v) was added in on Day 1. $CD8^+$ T cell function (**D**) $IFN\gamma^+$ gated on $CD8^+$; (**E**) $GzmB^+$ gated on $CD8^+$ was analyzed on Day 2 ($n = 4$). (**F**) $CD8^+$ T cell activation in the presence of the conditioned medium of Caco2 cells. Naive $CD8^+$ T cells were stimulated with plate-bound anti-CD3/CD28, in the presence of concentrated conditioned medium (CM) or control blank medium (Ctrl) (2.5% v/v). $CD8^+$ T cell activation ($CD44^+$ gated on $CD8^+$) was analyzed on Day 1. ($n = 4$). Data information: in (**A–F**), data are presented as mean ± SEM. Representative flow cytometry plots are shown in the left and corresponding quantified data in the right; two-tailed unpaired Student's *t* test. Data are representative of two independent experiments. *P* level < 0.01**. Source data are available online for this figure.

© The Author(s)

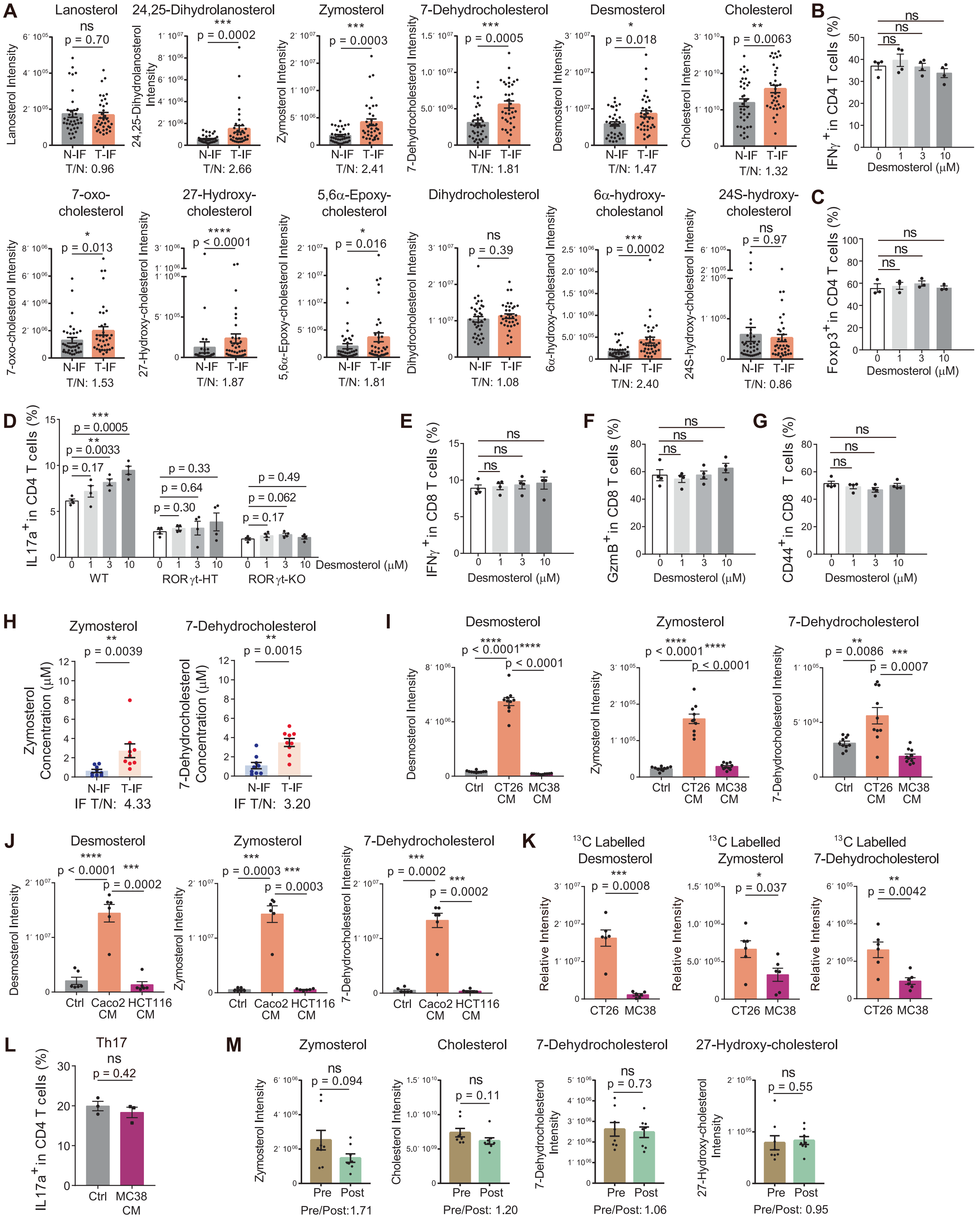
A
Lanosterol
24,25-Dihydrolanosterol
Zymosterol
7-Dehydrocholesterol
Desmosterol
Cholesterol
N-IF T-IF
T/N: 0.96
T/N: 2.66
T/N: 2.41
T/N: 1.81
T/N: 1.47
T/N: 1.32
7-oxo-cholesterol
27-Hydroxy-cholesterol
5,6α-Epoxy-cholesterol
Dihydrocholesterol
6α-hydroxy-cholestanol
24S-hydroxy-cholesterol
T/N: 1.53
T/N: 1.87
T/N: 1.81
T/N: 1.08
T/N: 2.40
T/N: 0.86
B
IFNγ+ in CD4 T cells (%)
Desmosterol (μM)
C
Foxp3+ in CD4 T cells (%)
D
IL17a+ in CD4 T cells (%)
WT
RORγt-HT
RORγt-KO
E
IFNγ+ in CD8 T cells (%)
F
GzmB+ in CD8 T cells (%)
G
CD44+ in CD8 T cells (%)
H
Zymosterol Concentration (μM)
7-Dehydrocholesterol Concentration (μM)
IF T/N: 4.33
IF T/N: 3.20
I
Ctrl
CT26 CM
MC38 CM
J
Caco2 CM
HCT116 CM
K
13C Labelled Desmosterol
13C Labelled Zymosterol
13C Labelled 7-Dehydrocholesterol
Relative Intensity
L
Th17
M
Pre Post
Pre/Post:1.71
Pre/Post: 1.20
Pre/Post: 1.06
Pre/Post: 0.95

© The Author(s)

Figure EV2. Accumulation of distal cholesterol precursors in the tumor microenvironment of MSS CRC.

(**A**) Intensity of sterols in the interstitial fluid of human MSS CRC tumors (T-IF) and paired adjacent normal tissues (N-IF). Fold change of the mean level of each sterol in tumor interstitial fluid vs normal tissue interstitial fluid (T/N) is labeled under each graph ($n = 36$). (**B**) Th1 induction in the presence of desmosterol at different concentrations. Naive CD4$^+$ T cells were stimulated with plate-bound anti-CD3/CD28 and cytokines to induce Th1 differentiation, in the presence of desmosterol or vehicle control. Th1 (IFNγ$^+$ gated on CD4$^+$) polarization were analyzed on Day 4 ($n = 4$). (**C**) Treg induction in the presence of desmosterol at different concentrations. Naive CD4$^+$ T cells were stimulated with plate-bound anti-CD3/CD28 and cytokines to induce Treg differentiation, in the presence of desmosterol or vehicle control. Treg (Foxp3$^+$ gated on CD4$^+$) polarization were analyzed on Day 4 ($n = 3$). (**D**) Th17 induction in the presence of desmosterol at different concentrations. Naive CD4$^+$ T cells from *Rorc*$^{-/-}$ (RORγt-KO), *Rorc*$^{+/-}$ (RORγt-HT) or *Rorc*$^{+/+}$ (WT) mice were stimulated plate-bound anti-CD3/CD28 and cytokines to induce Th17, in the presence of desmosterol or vehicle control. Th17 (IL-17a$^+$ gated on CD4$^+$) percentage was analyzed on Day 4 ($n = 4$). (**E**, **F**) CD8$^+$ T cell function in the presence of desmosterol at different concentrations. Naive CD8$^+$ T cells were stimulated with plate-bound anti-CD3/CD28, in the presence of desmosterol or vehicle control. CD8$^+$ T cell function (**E**) IFNγ$^+$ gated on CD8$^+$; (**F**) GzmB$^+$ gated on CD8$^+$ were analyzed on Day 2 ($n = 4$). (**G**) CD8$^+$ T cell activation in the presence of desmosterol at different concentrations. Naive CD8$^+$ T cells were stimulated with plate-bound anti-CD3/CD28, in the presence of desmosterol or vehicle control. CD8$^+$ T cell activation (CD44$^+$ gated on CD8$^+$) were analyzed on Day 1 ($n = 4$). (**H**) Absolute quantification of zymosterol and 7-dehydrocholesterol in the interstitial fluid of human MSS CRC tumors (T-IF) and paired adjacent normal tissues (N-IF). Fold change of the mean level of indicated sterol in tumors interstitial fluid vs normal tissue interstitial fluid (IF T/N) is labeled under graph ($n = 9$). (**I**, **J**) Intensity of indicated distal cholesterol precursors in the control blank medium (Ctrl) and the conditioned media of mouse MSS CRC cell CT26 ($n = 10$), mouse MSI CRC cell MC38 ($n = 10$), human MSS CRC cell Caco2 ($n = 6$), and human MSI CRC cell HCT116 ($n = 6$). Two-tailed unpaired *t* test was used when variances were similar, whereas a two-tailed unpaired *t* test with Welch's correction was used when variances were different. (**K**) Intensities of the ^{13}C-labeled sterols in CT26 and MC38 cells ($n = 6$). Two-tailed unpaired *t* test was used when variances were similar, whereas a two-tailed unpaired *t* test with Welch's correction was used when variances were different. (**L**) Th17 induction in the presence of the conditioned medium of MC38 cells ($n = 3$). (**M**) Intensity of the sterols in the plasma samples of human MSS CRC patients before (Pre) and after (Post) surgical resection of tumors. Fold change of the mean level of each sterol in plasma before vs after surgical resection (Pre/Post) is labeled under each graph ($n = 8$). Data information: in (**A–M**), data are presented as mean ± SEM. In (**B–G**, **L**) two-tailed unpaired Student's *t* test; data are representative of two independent experiments. In (**A**, **H**, **M**), two-tailed Wilcoxon matched-pairs signed-rank test. *P* levels < 0.05*, < 0.01**, < 0.001***, < 0.0001****.
Source data are available online for this figure.

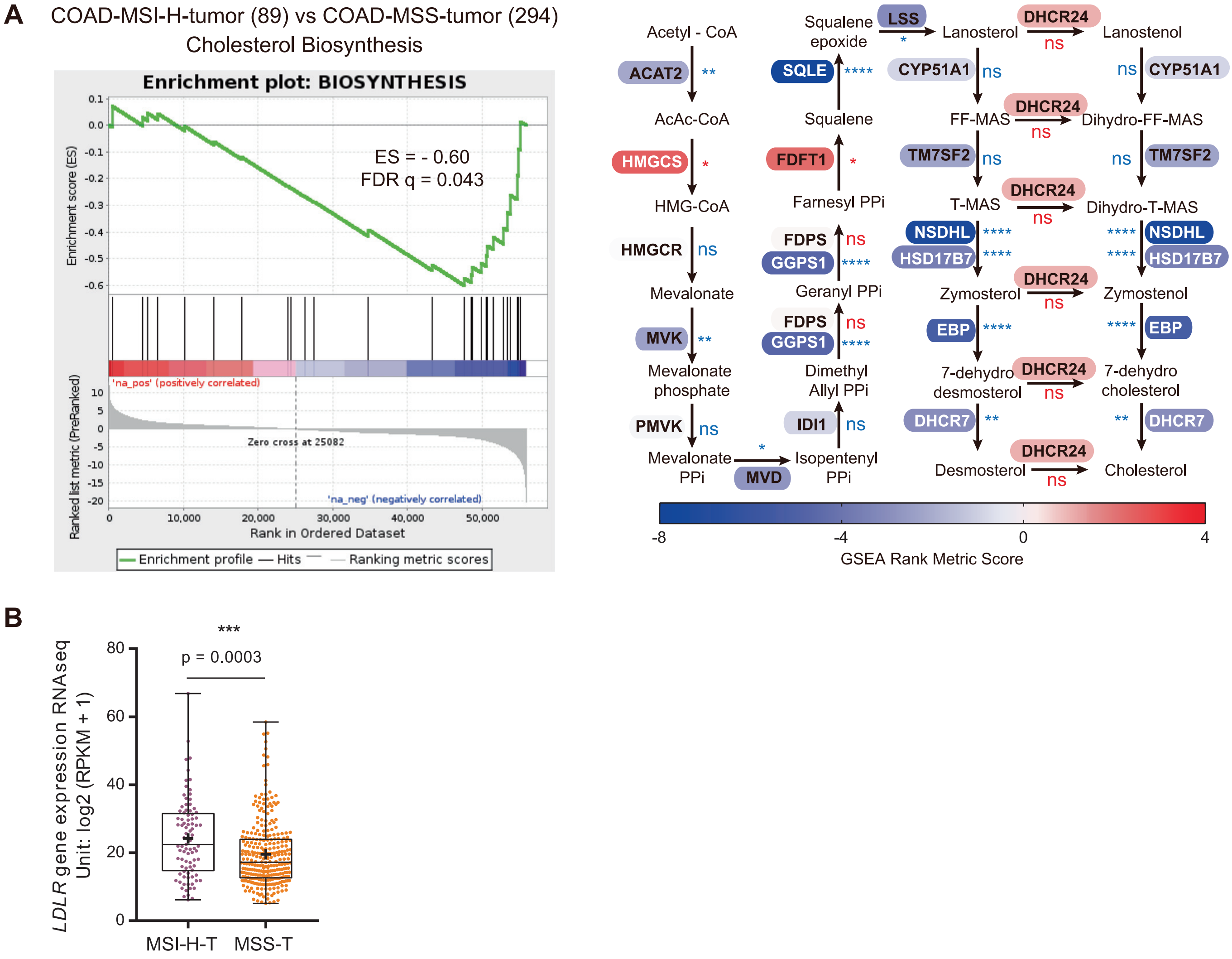

Figure EV3. MSS CRC and MSI CRC had different programs of cholesterol metabolism.

(**A**) Left: GSEA of cholesterol biosynthesis pathway in MSI-H CRC tumors ($n = 89$) and MSS CRC tumors ($n = 294$) in COAD. Right: GSEA rank metric score and transcriptional levels of enzymes in the cholesterol biosynthesis pathway. Blue and red represents downregulation and upregulation in tumor. *P* value of gene expression between tumor and normal tissue (two tailed Mann–Whitney test) is labeled by the side of each gene. (ns $P > 0.05$, $*P \le 0.05$, $**P < 0.01$, $***P < 0.001$, $****P < 0.0001$). (**B**) Transcriptional levels of *LDLR* in MSI tumors (MSI-H-T, $n = 89$) and MSS tumors (MSS-T, $n = 294$) from the COAD database. Whiskers denote minimum to maximum. Box indicates the interquartile range (25–75%); center line indicates the median. "+" indicates mean. Two tailed Mann–Whitney test. Source data are available online for this figure.

 © The Author(s)

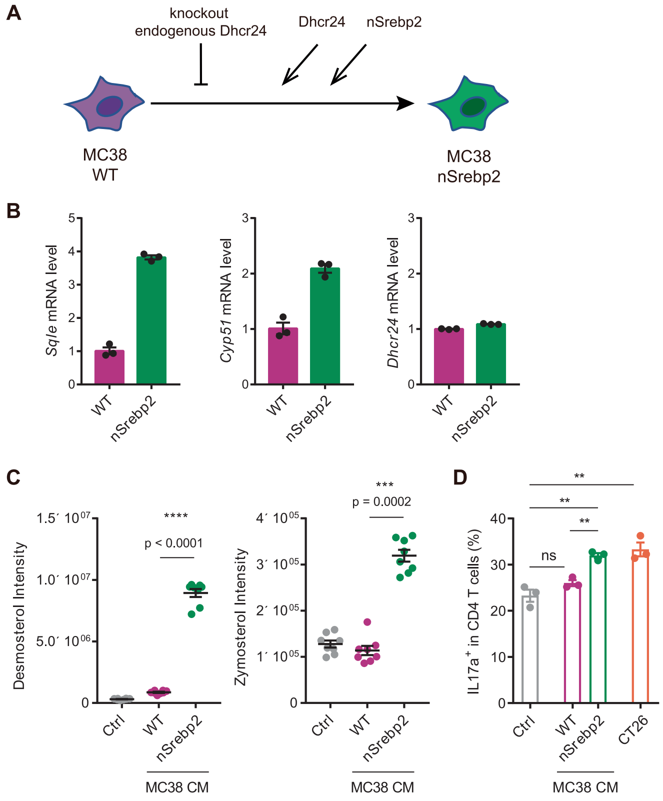

Figure EV4. Recapitulation of the asynchronous upregulation pattern of the cholesterol biosynthesis pathway in MSI CRC MC38 cells.

(**A**) Schematic illustration of the experimental procedure. (**B**) Transcriptional levels of *Sqle*, *Cyp51* and *Dhcr24* in wildtype (WT) and the genetic manipulated MC38 cells as described in panel A (nSrebp2), $n = 3$. (**C**) Intensity of indicated distal cholesterol precursors in the control blank medium (Ctrl), and the conditioned media of MC38 cells, $n = 8$. Two-tailed unpaired Student's *t* test if data fitted a normal distribution, Mann–Whitney test if data did not fit a normal distribution. (**D**) Th17 induction with CM of above-mentioned cells or CT26-CM as positive control, $n = 3$, two-tailed unpaired Student's *t* test. Data information: in (**B–D**), data are presented as mean ± SEM. *P* levels < 0.01**, < 0.001***, < 0.0001****. Source data are available online for this figure.

© The Author(s)

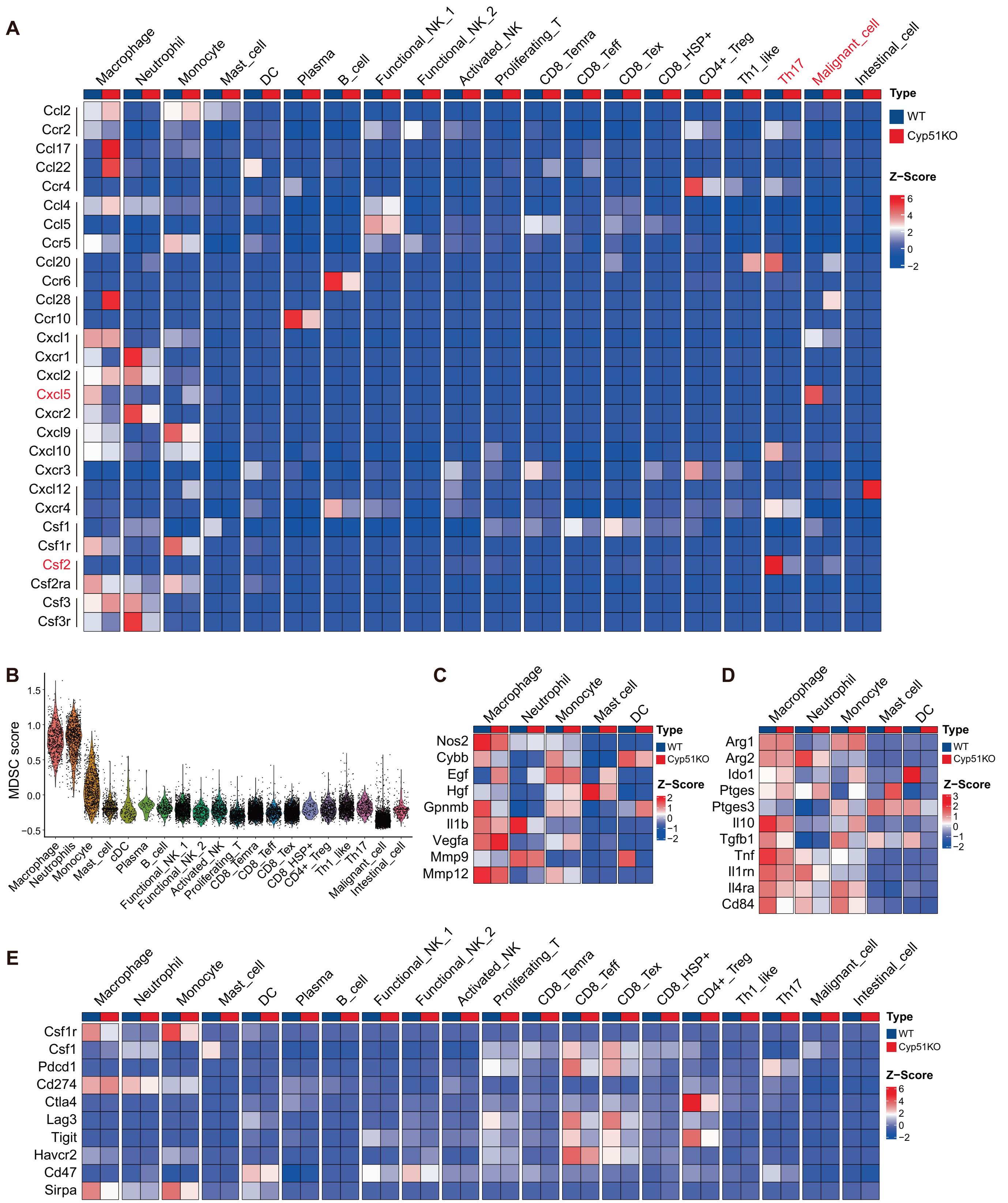
A
Macrophage
Neutrophil
Monocyte
Mast_cell
DC
Plasma
B_cell
Functional_NK_1
Functional_NK_2
Activated_NK
Proliferating_T
CD8_Temra
CD8_Teff
CD8_Tex
CD8_HSP+
CD4+_Treg
Th1_like
Th17
Malignant_cell
Intestinal_cell
Type
WT
Cyp51KO
Z-Score
6
4
2
0
−2
Ccl2
Ccr2
Ccl17
Ccl22
Ccr4
Ccl4
Ccl5
Ccr5
Ccl20
Ccr6
Ccl28
Ccr10
Cxcl1
Cxcr1
Cxcl2
Cxcl5
Cxcr2
Cxcl9
Cxcl10
Cxcr3
Cxcl12
Cxcr4
Csf1
Csf1r
Csf2
Csf2ra
Csf3
Csf3r
B
MDSC score
1.5
1.0
0.5
0.0
−0.5
Macrophage
Neutrophils
Monocyte
Mast_cell
cDC
Plasma
B_cell
Functional_NK_1
Functional_NK_2
Activated_NK
Proliferating_T
CD8_Temra
CD8_Teff
CD8_Tex
CD8_HSP+
CD4+_Treg
Th1_like
Th17
Malignant_cell
Intestinal_cell
C
Macrophage
Neutrophil
Monocyte
Mast_cell
DC
Type
WT
Cyp51KO
Z-Score
2
1
0
−1
−2
Nos2
Cybb
Egf
Hgf
Gpnmb
Il1b
Vegfa
Mmp9
Mmp12
D
Macrophage
Neutrophil
Monocyte
Mast_cell
DC
Type
WT
Cyp51KO
Z-Score
3
2
1
0
−1
−2
Arg1
Arg2
Ido1
Ptges
Ptges3
Il10
Tgfb1
Tnf
Il1rn
Il4ra
Cd84
E
Macrophage
Neutrophil
Monocyte
Mast_cell
DC
Plasma
B_cell
Functional_NK_1
Functional_NK_2
Activated_NK
Proliferating_T
CD8_Temra
CD8_Teff
CD8_Tex
CD8_HSP+
CD4+_Treg
Th1_like
Th17
Malignant_cell
Intestinal_cell
Type
WT
Cyp51KO
Z-Score
6
4
2
0
−2
Csf1r
Csf1
Pdcd1
Cd274
Ctla4
Lag3
Tigit
Havcr2
Cd47
Sirpa

 © The Author(s)

Figure EV5. Single-cell analysis of CT26 tumors (WT or Cyp51KO) in BALB/c mice as described in Fig. 6.

(**A**) Heatmap showing the gene expression levels of chemokines/growth factors and related receptors across all cell clusters. *Z*-score was calculated by mean expression level and indicated by color. (**B**) Violin plot showing the MDSC score in all cell clusters. The neutrophils and macrophages are major contributor of the suppressive function of myeloid derived suppressive cells (MDSC) (Dysthe and Parihar, 2020) in tumor. (**C**) Heatmap showing the expression levels of tumor growth/metastasis related genes across myeloid cell populations. *Z*-score was calculated by mean expression level and indicated by color. (**D**) Heatmap showing the expression levels of immunosuppression related genes across myeloid cell populations. *Z*-score was calculated by mean expression level and indicated by color. (**E**) Heatmap showing the expression levels of immune checkpoint genes across all cell populations. *Z*-score was calculated by mean expression level and indicated by color.
